# Supplementary material for: High-quality genome and methylomes illustrate features underlying evolutionary success of oaks
Source: Nat Commun. 2022 Apr 19;13:2047. doi: 10.1038/s41467-022-29584-y (PMC9018854; doi:10.1038/s41467-022-29584-y)
Supplement: Supplementary file 1 — Supplementary Information [file 41467_2022_29584_MOESM1_ESM.pdf]

Supplementary Information

High-quality genome and methylomes illustrate features underlying evolutionary success of oaks

Victoria L. Sork[1,2*, **], Shawn J. Cokus[3**], Sorel T. Fitz-Gibbon[1**], Aleksey V. Zimin[4,5], Daniela Puiu[4], Jesse A. Garcia[1], Paul F. Gugger[6], Claudia L. Henriquez[1], Ying Zhen[1], Kirk E. Lohmueller[1,7], Matteo Pellegrini[3], and Steven L. Salzberg[4,8]

Affiliations:

[1] Department of Ecology and Evolutionary Biology, University of California, Los Angeles, CA 90095-1438

[2] Institute of the Environment and Sustainability, University of California, Los Angeles, CA 90095

[3] Department of Molecular, Cell, and Developmental Biology, University of California, Los Angeles, CA 90095-7239

[4] Center for Computational Biology, Whiting School of Engineering, Johns Hopkins University, Baltimore, Maryland 21218

[5] Department of Biomedical Engineering, Johns Hopkins University, Baltimore, Maryland 21218

[6] University of Maryland Center for Environmental Science, Appalachian Laboratory, Frostburg, MD 21532

[7] Department of Human Genetics, David Geffen School of Medicine, University of California, Los Angeles, CA 90095

[8] Departments of Biomedical Engineering, Computer Science, and Biostatistics, Johns Hopkins University, Baltimore, Maryland 21218

*Corresponding author: Victoria L. Sork, vlsork@ucla.edu
**Authors contributed equally

TABLE OF CONTENTS FOR SUPPLEMENTARY INFORMATION

Supplementary Note 1. Sample collection, library preparation, sequencing, and initial data processing 3
A. Valley oak reference genome 3
B. Valley oak resequenced genomes 4
Supplementary Table 1. Localities of resequenced *Q. lobata* adults 5
C. Transcriptomes 5
D. Methylomes 6

Supplementary Note 2. Validation and orientation of chromosomes 7
Supplementary Figure 1. *Q. lobata* and *Q. robur* assemblies vs. *Q. robur* x *Q. petraea* linkage map: 1-D view 8
Supplementary Figure 2. *Q. lobata* and *Q. robur* assemblies vs. *Q. robur* x *Q. petraea* linkage map: 2-D view 9
Supplementary Table 2. Statistics of *Q. lobata* and *Q. robur* assemblies vs. *Q. robur* x *Q. petraea* linkage map 10
Supplementary Figure 3. Misassembled mitochondrial sequence in pre-final *Q. lobata* version 3.0 chromosome 1 11

Supplementary Note 3. Analysis of heterozygosity 12
Supplementary Figure 4. Distribution of Tajima's π across the *Q. lobata* genome 12
Supplementary Figure 5. Distribution of heterozygosity rate (Heterozygosity per bp) across the *Q. lobata* genome 13

Supplementary Note 4. Demographic analysis 14
Supplementary Figure 6. PSMC' inference on the *Q. lobata* reference genome using different generation times and mutation rates 16
Supplementary Figure 7. Full demographic models inferred by PSMC' 17
Supplementary Figure 8. Predicted heterozygosity for 1 Mbp regions for all trim possibilities for each genome type 18
Supplementary Figure 9. Predicted heterozygosity for 120 simulated 1 Mbp regions of the best fitting models for each genome type 19
Supplementary Figure 10. Predicted heterozygosity of full untrimmed PSMC' models compared to that of best-fitting trimmed ancestral models 20

Supplementary Note 5. Assessment of amino acid diversity in the large DUF247 block on chromosome 4 21
Supplementary Figure 11. DUF247 PCG amino acid diversity 21
Supplementary Figure 12. Phylogeny of *Q. lobata* and *Q. robur* DUF247 PCGs 22

Supplementary Note 6. Repetitive sequences 23
Additional findings and methods 23
Supplementary Figure 13. Unsupervised clustering indicates that the dominant chromosome-scale distributional features of repeats in *Q. lobata* are correlated with distance to the centromeres 25
Supplementary Figure 14. Average centromeric distance summarizes repeat per-SF distribution of distances well 26

Supplementary Note 7. Gene model statistics and possible R-genes in *Q. lobata*, *Q. robur*, and *Q. suber* 27
Supplementary Table 3. Statistics of protein-coding gene (PCG) models for *Q. lobata*, *Q. robur*, and *Q. suber* 27
Supplementary Figure 15. Log–log frequency versus size of tandem-like duplicated gene blocks 28
Methods for identification of possible R-genes 28
Supplementary Table 4. R-gene domain/motif analysis partitioning all *Q. lobata*, *Q. robur*, and *Q. suber* PCGs 29

Supplementary Note 8. Methylomes and analysis of methylation patterns 30
Supplementary Figure 16. Genome methylation levels for three tissues and three methylation contexts 30
Supplementary Figure 17. CHH methylation levels in bud tissue across repeats 31
Supplementary Figure 18. Gene methylation metaplots, with and without introns 32
Supplementary Figure 19. Subcontext methylation for *Q. lobata* chromosomes 1 to 12 in 1 Mbp windows 33
Supplementary Figure 20. Intergenic subcontext mCHH by size of region 36
Supplementary Figure 21. Genic region methylation of oak in comparison with 34 angiosperms 37
Supplementary Figure 22. Chromosomal overviews of methylation and PCGs in oak compared with 24 angiosperms 38
Supplementary Figure 23. Subcontext methylation for *Populus trichocarpa* chromosomes in 1 Mbp windows 40

Supplementary Note 9. Additional Tables 41
Supplementary Table 5. Top Pfam accessions enriched in the most heavily tandemly duplicated PCG families 41
Supplementary Table 6. Within 23,174 non-tandemly duplicated genes, top hypergeometrically-enriched accessions for those genes participating in at least two SSB-supporting gene pairs 42
Supplementary Table 7. Most abundant Pfam accessions in *Q. lobata*, and their frequency in selected other plant species 43

Supplementary References 44

Supplementary Note 1. Sample collection, library preparation, sequencing, and initial data processing

A. Valley oak reference genome

All tissues collected from either *Quercus lobata* SW786 at Sedgewick Reserve in Santa Barbara, CA, or other *Q. lobata* trees throughout the California species range (**Supplementary Table 1**) were placed immediately on dry ice. Plant tissue was stored at –80 °C until the day of extraction. The voucher specimen for tree SW786, collected March 2017, is D. O. Burge 2309, deposited at UC Davis (DAV). This healthy and prolific acorn producing adult has been included in several quantitative genetic and genomic studies [1, 2, 3, 4, 5].

Illumina paired end and mate pair libraries. Leaf tissue for Illumina libraries was collected September 2014. Details for extraction of total genomic DNA, library preparation, and sequencing are described in Sork, Fitz-Gibbon [6]. Briefly, DNA extractions were by a CTAB protocol. 266M HiSeq 2500 read pairs of 250 nt (175x coverage) were generated from two short insert paired end libraries, one with PCR enrichment and one without. 159M HiSeq 2500 read pairs of 150 nt (56x coverage) were generated from nine mate pair libraries of length 2.9 kb to 12 kb.

Pacific Biosciences whole genome SMRTbell libraries. Leaf tissue for the PacBio DNA libraries was collected April 2016. High molecular weight (HMW) DNA was obtained through a nuclei isolation protocol based on "Preparing *Arabidopsis* Genomic DNA for Size-Selected ~20 kb SMRTbell™ Libraries" (Pacific Biosciences of California, Inc., 2013) and the Sean Gordon protocol [7]. Ten grams of fresh plant tissue was flash frozen with liquid nitrogen and ground with a mortar and pestle three times to obtain a fine powder, and transferred to a chilled Erlenmeyer flask. 300 mL of fresh sucrose-based extraction buffer (SBE) was prepared (2% w/v PVP, 10% v/v TKE, 500 mM sucrose, 4 mM spermidine trihydrochloride, 1 mM spermine tetrahydrochloride, 0.1% w/v ascorbic acid, and 0.13% w/v sodium diethyldithiocarbamate, and adjusted to a pH of 9.0–9.1 with 1M KOH) with 600 µL of ß-mercaptoethanol (BME). 185 mL of SBE+BME was added to the ground tissue and placed on ice for 12–20 minutes with continuous swirling until the powder dissolved. The homogenate was filtered through two layers of Grade 50 cheesecloth (Lions Services, North Carolina, USA) into a clean 500 mL beaker, using an extra 15 mL of SBE+BME solution to ensure all particulates passed through the cheesecloth. Then, 10 mL of cold 10% Triton was added to the beaker, slowly along the side over the course of two minutes, while gently stirring with a magnetic bar, and kept on ice for eight minutes with intermittent gentle swirling. The mixture was transferred into 4x 50 mL polypropylene Falcon tubes, and spun in a centrifuge at 650 x *g* (1,970 rpm) for 15 min at 4 °C. The supernatant was discarded and the pellet was gently resuspended in 10 mL of cold SBE+BME. The mixture was transferred into 2x 50 mL polypropylene Falcon tubes and SBE+BME was added until each tube had a final volume of 30 mL. These were centrifuged at 650 x *g* (1,970 rpm) for 15 min at 4 °C. The supernatants were discarded and the pellets were resuspended in 1.44 mL of TE. The mixture was divided into 4x 2 mL tubes and 95 µL of cold 1M NaCl and 240 µL of cold 10 mg/ml RNase A was added to each tube and incubated at 65 °C for 30 min to digest RNA. Then 24 µl of cold 10 mg/ml Proteinase K was added to each tube and inverted gently 2x. Then 95 µl of room temperature 10% SDS was added to each tube, inverted gently 2x, incubated at 45 °C for 60 min to digest proteins, and brought to room temperature. Samples from 2 mL tubes were combined into 2x 15 mL Falcon tubes and 2.178 mL (or 1 volume) of phenol:chloroform:isoamyl alcohol was added and tubes were inverted gently, vortexed for two seconds, and centrifuged for five minutes at room temperature at 1,500 x *g* (2,300 rpm). The aqueous layer was transferred to new 15 mL Falcon tubes and the extraction with 1 volume of phenol:chloroform:isoamyl alcohol was repeated until the interface was clear. The clear extraction was then divided into 6x 2 mL tubes (~670 µL in each tube) and 70 µL (or ~0.1 volume) of 3M NaOAc (pH 5.2), and 750 µL (or ~1 volume) cold isopropanol was added and placed in a –20°C freezer for 30–60 minutes or at 4 °C overnight. The tubes were centrifuged for 30 minutes at 13,000 rpm at 4 °C and the supernatants discarded. The pellets were then washed 2x with 500 µl 70% ethanol and centrifuged for >10 min at 13,000 rpm at 4 °C. Pellets were spun for two minutes at 13,000 rpm at 4 °C and the ethanol was decanted with a pipette. The pellets were air dried at room temperature for 10 min and resuspended in 30–50 µL TE per tube and allowed to rest in a 2–8 °C fridge overnight to elute DNA. DNA was analyzed using a Nanodrop ND-1000 Spectrophotometer (Thermo Fisher Scientific, Waltham, MA), run on a 0.8% agarose gel with 1 kb plus ladder, and quantified using a Qubit 3.0 Fluorometer (Life Technologies, Carlsbad, CA).

The HMW DNA samples were sent to the DNA Technologies & Expression Analysis core Laboratory at the University of California, Davis. The samples were purified using the "Guidelines for Using a Salt:Chloroform Wash

to Clean up gDNA" protocol (Pacific Biosciences of California, Inc., 2014), then prepared into libraries using the "Procedure & Checklist – Preparing > 30 kb SMRTbell™ Libraries Using the Megaruptor® Shearing and BluePippin™ Size-Selection System" Protocol (Pacific Biosciences of California, Inc., 2016). Three libraries were prepared with 8 kb lower cut-off, 10 kb lower cut-off, and 20 kb lower cut-off size selections by BluePippin™ (Sage Science, Beverly, MA, USA). Seventeen v3 SMRT cells were run for the 8 kb cut-off, 11 cells for the 10 kb cut-off, and eight cells for the 20 kb cut-off. Sequencing polymerase was version 6 and chemistry was version 4 (P6C4). SMRT cells were sequenced on a RS II sequencer yielding 80x genome coverage.

Dovetail whole genome HiC library. Leaf tissue was collected March 2017, of which 1 gram was sent to Dovetail Genomics, Scotts Valley, CA, USA. A Dovetail HiC library was prepared in a similar manner as described previously [8]. Briefly, for each library, chromatin was fixed in place with formaldehyde in the nucleus and then extracted. Fixed chromatin was digested with DpnII, the 5' overhangs filled in with biotinylated nucleotides, and then free blunt ends were ligated. After ligation, crosslinks were reversed and the DNA purified from protein. Purified DNA was treated to remove biotin that was not internal to ligated fragments. The DNA was then sheared to ~350 bp mean fragment size and sequencing libraries were generated using NEBNext Ultra enzymes and Illumina-compatible adapters. Biotin-containing fragments were isolated using streptavidin beads before PCR enrichment of each library. The libraries were sequenced on an Illumina HiSeq X to produce 454M 151+151 bp paired end reads, which provided 6,875x physical coverage of the genome (10–10,000 kb pairs).

B. Valley oak resequenced genomes

Leaf tissue samples for whole genome sequencing used in the demography studies (described below in **Supplementary Note 4. Demographic analysis**) were collected from 19 *Q. lobata* adults (**Supplementary Table 1**). Total genomic DNA was extracted from frozen leaf tissue using a prewash method [9], followed by a modified CTAB protocol [10] or the Plant Dneasy Kit protocol (Qiagen, Germany). Plants were frozen in liquid nitrogen and ground using a Mixer Mill MM301 (Retsch, Germany). The prewash method was repeated up to 3x until a clear supernatant was achieved. The resultant pellet was used in a modified CTAB protocol in which the chloroform:isoamyl (24:1) step was repeated twice. DNA was analyzed using a Nanodrop ND-1000 Spectrophotometer (Thermo Fisher Scientific, Waltham, MA) and quantified using a Qubit 3.0 Fluorometer (Life Technologies, Carlsbad, CA).

Libraries were prepared following the Nextera XT DNA Library Prep Kit guidelines (Illumina, San Diego, CA). Dual index combinations for each sample were chosen based on the Nextera Low Plex Pooling Guidelines (Illumina, San Diego, CA). Samples were multiplexed in the following layout: eight lanes of six libraries per lane on 2016–12–09; three lanes of eight libraries per lane on 2017–10–11; seven lanes of 3–4 libraries per lane on 2018–09–06 (based on coverage needs), and 11 lanes of 2 libraries per lane on 2019–04–01 (based on coverage needs). Libraries were analyzed on an Agilent D1000 Screen Tape System on an Agilent 2200 TapeStation (Agilent Technologies, Santa Clara, CA, USA), and sequenced using an Illumina HiSeq 4000 at the UCLA Stem Cell Center Core facility with 100 bp paired end reads to coverage 17x–32x (mean 24x) as assessed by GATK 3.7-0-gcfedb67 `DepthOfCoverage –countType COUNT_FRAGMENTS –minMappingQuality 20 –minBaseQuality 10`.

Illumina reads were adapter trimmed and quality checked using Trim Galore! 0.4.4 https://www.bioinformatics.babraham.ac.uk/projects/trim_galore/ , calling Cutadapt 1.9.1 [11] with no quality trimming and minimum length 20 bp. Trimmed reads were aligned to the *Q. lobata* 3.0 reference genome using bwa mem 0.7.12-r1039 [12] and read duplicates were flagged with Picard tools MarkDuplicates 2.13.2-SNAPSHOT (http://broadinstitute.github.io/picard/). Variants and non-variants were called for all sites of each sample with GATK 3.7-0-gcfedb67 `HaplotypeCaller –heterozygosity 0.01 –indel_heterozygosity 0.001 -newQual –emitRefConfidence GVCF`, followed by genotyping of the whole population together with `GenotypeGVCFs --includeNonVariantSites –heterozygosity 0.01 –indel_heterozygosity 0.001`.

Supplementary Table 1. Localities of resequenced *Q. lobata* adults. Sample IDs and locations are given for the 19 *Q. lobata* individuals sampled throughout the species range and selected for whole genome resequencing for use in the demography study.

Sample ID	Locality Name	Latitude (°)	Longitude (°)
QL.CHE.100	Cheeseboro (CHE)	34.1636	–118.7241
QL.CHI.3	Chico (CHI)	39.7119	–121.7842
QL.CLO.4	Clearlake Oaks (CLO)	39.0219	–122.7135
QL.CVD.8	Cloverdale (CVD)	38.8544	–123.0319
QL.FHL.5	Fort Hunter Liggett (FHL)	35.9804	–121.2328
QL.GRV.2	Gravelly Valley (GRV)	39.4302	–122.9754
QL.GRV.7	Gravelly Valley (GRV)	39.4485	–122.9640
QL.JAS.5	Jasper Ridge (JAS)	37.4032	–122.2436
QL.LAY.5	Laytonville (LAY)	39.7460	–123.5242
QL.LAY.6	Laytonville (LAY)	39.6722	–123.4807
QL.LYN.4	Lynch Canyon Road (LYN)	35.7878	–120.9391
QL.MAR.B	Mariposa (MAR)	37.4611	–119.8797
QL.MCK.5	Middle Creek CG (MCK)	39.2524	–122.9516
QL.MOH.3	Morgan Hill (MOH)	37.1649	–121.7148
QL.MTR.3	Mountain Ranch (MTR)	38.2750	–120.5058
QL.PEN.5	Penn Valley (PEN)	39.2034	–121.1902
QL.ROV.3	Round Valley (ROV)	39.7483	–123.2484
QL.SUN.5	Sunol (SUN)	37.5987	–121.8751
QL.UKI.5	Ukiah (UKL)	39.0924	–123.2197

C. Transcriptomes

Pacific Biosciences RNA long read (Iso-Seq) libraries for tree SW786 bud, leaf, and stem tissues. Bud, leaf, and stem tissue samples for Iso-Seq libraries were collected from tree SW786 in October 2017. RNA extractions were performed between November 6–8, 2017 using a modified version of the Conifer RNA prep protocol from the Cronn Lab (https://openwetware.org/wiki/Conifer_RNA_prep) and a Spectrum Plant Total RNA kit (Sigma, St. Louis, MO, USA). Plant tissues (100 mg each of leaves, buds, and stems) were flash frozen in liquid nitrogen and ground with a mortar and pestle to a fine powder. Powdered tissues were transferred to cold 2 mL tubes and 1.8 mL of cold RNA Extraction Buffer + DTT was added. RNA Extraction Buffer consisted of 8M Urea, 3M LiCl, 1% polyvinylpyrrolidone K-60, and 5 mM DTT (added just before use; 1M stock). Tubes were then vortexed for 30 seconds, incubated at 4 °C for 30 minutes, and centrifuged at 4 °C for 30 minutes at 20,000 rcf. The supernatant was discarded and the pellet was used as the starting material for Spectrum Plant Total RNA kit Protocol A, adding 750 µL of Binding Solution, and performing on-column Dnase I digestion. RNA quality and quantity were assessed using an Agilent RNA ScreenTape System on an Agilent 2200 TapeStation (Agilent Technologies, Santa Clara, CA, USA).

RNA was further prepared following the "Guidelines for Preparing cDNA Libraries for Isoform Sequencing (Iso-Seq™) User Bulletin" (Pacific Biosciences of California, Inc., 2014) and the "Procedure & Checklist – Iso-Seq™ Template Preparation for Sequel™ Systems" (Pacific Biosciences of California, Inc., 2017). PolyA positive RNA was extracted from total RNA using an Ambion® Poly(A) Purist™ MAG Kit (Invitrogen, Carlsbad, CA, USA) following the manufacturer's protocol. First strand cDNA synthesis was performed using a SMARTer® PCR cDNA Synthesis Kit (Takara Bio, Inc., Kusatsu, Shiga Prefecture, Japan), with three reactions using 3.5 µL of PolyA positive RNA per

tissue, for a total of nine first strand synthesis reactions. Each of nine reactions were diluted with 90 µL of EB buffer, then pooled according to tissue type. PCR cycle optimization resulted in the following PCR conditions, with 24x 50 µL reactions per tissue type: 95 °C for 2 minutes for initial denaturation; then *n* cycles (*n* = 10, 12, and 14 for leaf, bud, and stem) of 98 °C for 20 seconds, 65 °C for 15 seconds, and 72 °C for 4 minutes; then 72 °C for 5 minutes for the final extension. Twelve reactions per tissue were pooled for 1x AMPure XP (Beckman Coulter, Inc., Pasadena, CA, USA) bead purification, and 12 reactions per tissue type were pooled for 0.4x AMPure XP bead purification. Samples were sent to the DNA Technologies & Expression Analysis Core Laboratory at the University of California, Davis for size selection, enrichment, library preparation, and sequencing. A second bead size selection was performed, 1x and 0.4x (Fractions 1 and 2, respectively) for two size fractions and a size selection of 5–10 kb using the BluePippin Size Selection System. Six libraries were made from these different size selections following the "Procedure & Checklist – Iso-Seq™ Template Preparation for Sequel™ Systems" protocol: Libraries 1, 2, and 3 (leaf, bud, and stem) full length (Fractions 1 and 2), and Libraries 4, 5, and 6 (leaf, bud, and stem) 5–10 kb size selection (Fraction 3). For each of bud, leaf, and stem, libraries were pooled for sequencing (5:1, full length: Fraction 3) for a total of three libraries that were each sequenced on a single cell. The cells were loaded and sequenced on a Sequel using Magbead / v2 SMRT cells / P2.1C2.1 (polymerase version 2.1, chemistry version 2.1).

Raw reads (from subreads BAM files) for each of the three tissues were processed using PacBio's Iso-Seq 'classify' bioinformatics pipeline [7], although clustering was skipped and replaced with filtering of the Minimap2 read alignments (described in main text **Methods**). Specifically, the two Iso-Seq 'classify' bioinformatics pipeline steps were (first) `ccs –minLength=50 –maxLength=12000 –minPasses=1 –minPredictedAccuracy=0.8 --minZScore=-999 –maxDropFraction=0.8`, and (second) `pbtranscript classify –min_seq_len 100`. The resulting put–ively full length non-chimeric reads were aligned to the genome assembly with intron-enabled Minimap2 [13] `-ax splice -uf –secondary=no`. Final aligned reads and raw subread bam files are available as GEO accession GSE174827.

Illumina RNA short read (RNA-Seq) libraries for tree SW786 bud, leaf, and stem tissues. Bud, leaf, and stem tissue samples for RNA-Seq libraries were collected from tree SW786 in October 2017. RNA was extracted February 16, 2018. Total RNA was depleted of rRNA using a Ribo-Zero rRNA Removal Kit (Plant Leaf) (Illumina, San Diego, CA, USA). Total RNA input amounts were 4.2 µg for bud, 5 µg for leaf, and 4.1 µg for stem. The Individual Washing option was used for washing the magnetic beads, and the 500 ng–to–1.25 µg input RNA recipe was used for hybridizing the probes. RNA samples depleted of rRNA were cleaned with ethanol precipitation, incubated with Elute, Prime Fragment High Mix at 85 °C for 6 minutes, and quantified using t Qubit® RNA HS Assay Kit with a Qubit 3.0 Fluorometer (Life Technologies, Carlsbad, CA). Libraries were constructed using the TruSeq Stranded Total RNA protocol with a positive control (Illumina, San Diego, CA, USA). First strand and second strand cDNA synthesis, dA-tailing, ligation, purification, and enrichment steps were performed following the manufacturer's instructions (Illumina, San Diego, CA, USA). Libraries were analyzed using an Agilent D1000 Screen Tape System on an Agilent 2200 TapeStation (Agilent Technologies, Santa Clara, CA, USA). Fragments were found to be too small (~275 bp), so an extra size selection step was performed with AMPure XP beads at a concentration of 0.65x, yielding fragments in the 400–700 bp range. Libraries were quantified using a Qubit® dsDNA BR Assay Kit on a Qubit® 3.0 Fluorometer (Life Technologies, Carlsbad, CA). Libraries were pooled and sequenced on an Illumina HiSeq 4000 at the UCLA Broad Stem Cell Center Core facility.

D. Methylomes

Whole genome bisulfite libraries for tree SW786 bud, catkin, and young leaf tissues. Tissue samples for assaying methylation were collected from tree SW786 on three different months of 2017: February (bud), March (catkin), and April (young leaves). Total genomic DNA was extracted from frozen leaf tissue on August 24, 2017 using a prewash method (Li et al., 2007) followed by a modified CTAB protocol (Doyle and Doyle, 1987). Plants were frozen in liquid nitrogen and ground using a Mixer Mill MM301 (Retsch, Germany). The prewash method was repeated up to 3x until a clear supernatant was achieved. The resultant pellet was then used in a modified CTAB protocol in which the chloroform:isoamyl (24:1) step was repeated twice. Total genomic DNA at a concentration of 500 ng in 60 µL was sonicated using an S2 Focused-ultrasonicator (Covaris, Woburn, MA, USA) for 60 seconds to obtain fragments in the 200–300 bp range (duty cycle: 10%, intensity: 5, cycles/burst: 200, mode: frequency sweeping).

Using reagents from the TruSeq Nano DNA Library Prep Kit (Illumina, San Diego, CA, USA), sheared DNA samples were end repaired as in the TruSeq protocol, then purified with AMPure beads at a concentration of 1.6x. Fragments were adenylated and adapters ligated as in a TruSeq protocol, except that 1 µL of Illumina TruSeq Adapters were used in the final reactions. The ligation reactions were purified with AMPure beads at a concentration of 1.2x, then purified with beads again at a concentration of 1x. Samples were treated with bisulfite using an EpiTect kit (Qiagen, Hilden, Germany) according to the manufacturer's protocol, except the bisulfite DNA conversion was performed twice for a total of 10 hours of incubation. Two amplification reactions were performed for each sample (20 µL of bisulfite converted DNA, 2.5 µL Illumina TruSeq primer cocktail, 25 µL MyTaq Mix (Bioline, Taunton, MA), and 2.5 µL H_2O per PCR reaction) under the following conditions: initial denaturation at 98 °C for 30 s; 12 cycles of 98 °C for 15 s, 60 °C for 30 s, and 72 °C for 30 s; and final extension at 72 °C for 5 min. The final PCR products were purified using AMPure XP beads. Libraries were analyzed on the Agilent D1000 Screen Tape System on an Agilent 2200 TapeStation (Agilent Technologies, Santa Clara, CA, USA). All samples were sequenced once on a single Illumina HiSeq 4000 lane with 100 bp single end reads at the UCLA Broad Stem Cell Core facility, yielding median genomic coverage of 18x–19x.

Reads were trimmed and inspected with Trim Galore! 0.4.4 [14], which calls Cutadapt [11] and FastQC [15], with quality score cutoff 20 and minimum length 80 bp. Trimmed reads were aligned to the *Q. lobata* 3.0 reference assembly using Methylpy 1.4.6 [16], which converts the reference genome for alignment of BS-Seq data, aligns with Bowtie_2 [17], estimates the bisulfite non-conversion rate from an unmethylated control (in our case, the *Q. lobata* chloroplast), performs binomial tests to distinguish methylated sites above the estimated non-conversion noise level, and outputs counts of covering methylated and unmethylated reads for each genomic cytosine site. Parameters for the Methylpy single end pipeline command were `–remove-clonal True –min-mapq 30 –min-base-quality 1 --trim-reads False –unmethylated-control chrC –binom-test True –min-cov 3`. Aligned reads were inspected for methylation bias by read position using MethylDackel 0.4.0 mbias [18] and sequencing depth was assessed with DeepTools 3.1.2 plotCoverage [19].

Supplementary Note 2. Validation and orientation of chromosomes

To confirm the correspondence of the twelve longest *Q. lobata* 3.0 assembly scaffolds with chromosomes, we used an existing moderate density linkage map of *Q. robur* x *Q. petraea* ("2015 composite") [20] consisting of 4,217 SNP markers in twelve linkage groups (LGs), after dropping 22 named SNPs associated to two LGs each. *Q. robur* is also in section *Quercus* and is probably separated from *Q. lobata* by 30M years [21]. These SNPs are a subset of 7,913 [22] identified by typically 100 nt of context on both sides. We aligned marker sequences to our assembly with BLASTN 2.2.30+ ($E < 10^{-15}$), retaining all hits for each query with ≥ 97% bitscore of the top hit. Approximately 82% of the 7,913 were genetically mapped to *Q. lobata* uniquely, 14% to exactly two locations, 3% to more than two, and 1% were unmapped; all hits had nt identity > 69% and aligned ≥ 57 nt, and 90%+ variously had nt identity > 93%, aligned ≥ 105 nt, covered > 52% of the query, and had $E \leq 10^{-42}$. Of the 4,217 SNPs on an LG, we dropped 1% that were genetically unmapped to *Q. lobata*, kept 86% uniquely mapped, dropped 5% mapped to multiple scaffolds, kept 8% that were multiply mapped but to a single scaffold with span of all hits ≤ 2 Mbp wide, and dropped 0.5% that were multiply mapped with wider spans. Analysis with the *Q. robur* assembly was with the same procedure and parameters. We found a predominantly monotonic one-to-one correspondence between LGs and the twelve largest scaffolds of our assembly (**Supplementary Figure 1**, **Supplementary Figure 2**, and **Supplementary Table 2**), and thus renamed our scaffolds as chromosomes, adopting the LG (and *Q. robur*) numbering (but not necessarily the LG orientation, where we instead follow the *Q. robur* assembly — hence, we essentially adopt both the numbering and orientation of *Q. robur*).

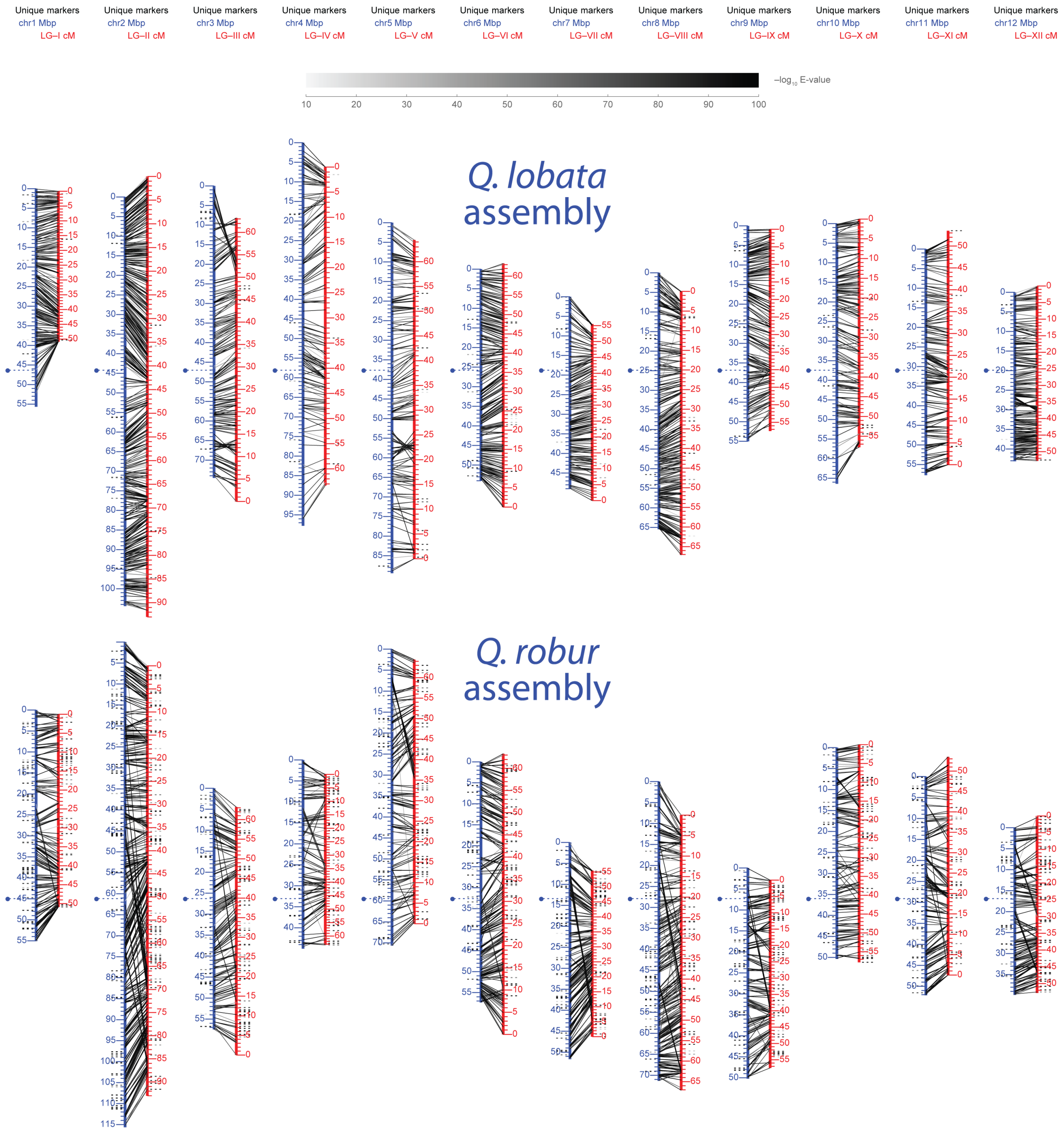

Supplementary Figure 1. *Q. lobata* and *Q. robur* assemblies vs. *Q. robur* x *Q. petraea* linkage map: 1-D view. Lines connect sequence context-defined SNPs in the linkage map (blue centimorgan scales) to assembly locations (red Mbp scales) via sequence alignment of the typically ±100 nt of sequence context for the SNP.

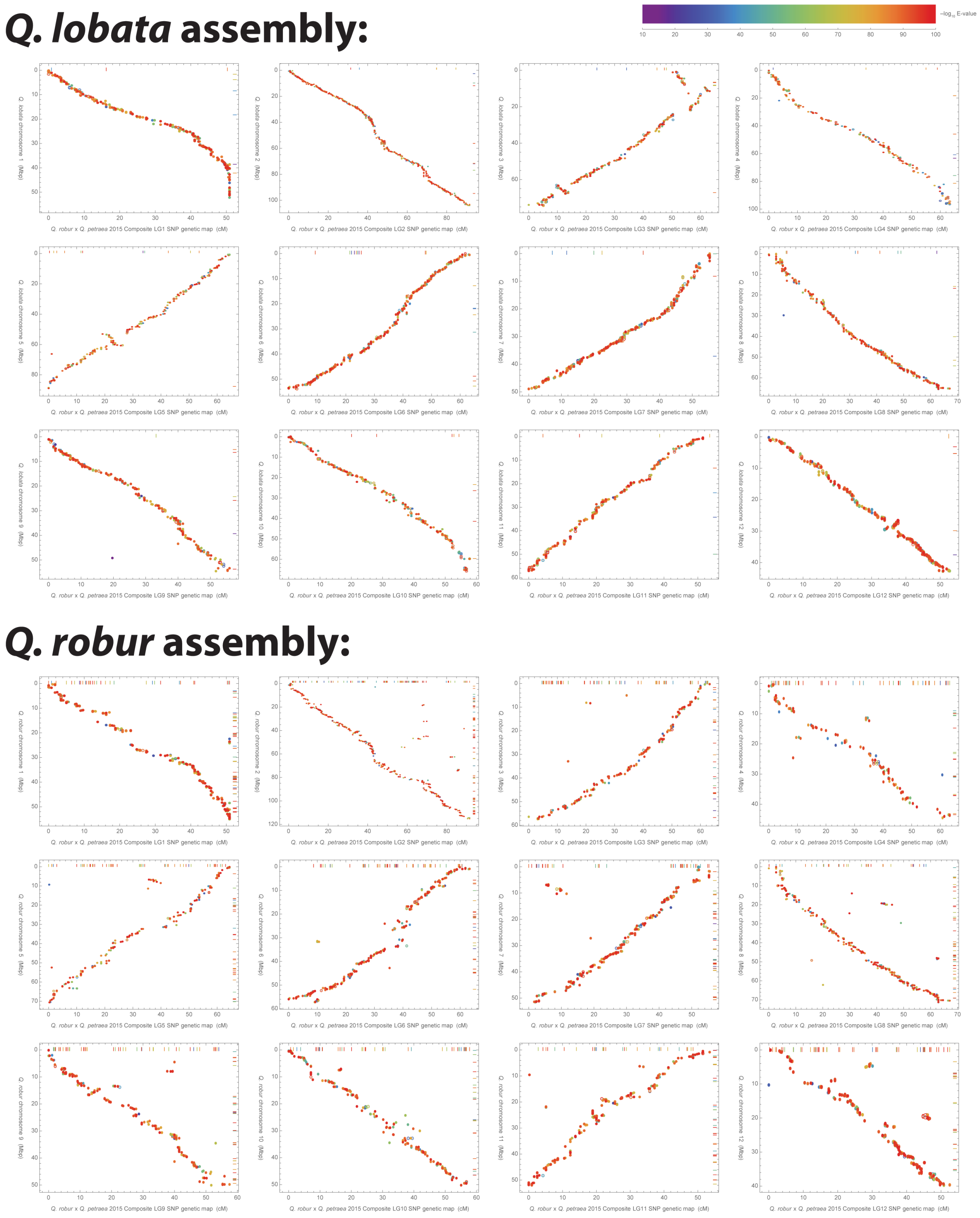

Supplementary Figure 2. *Q. lobata* and *Q. robur* assemblies vs. *Q. robur* x *Q. petraea* linkage map: 2-D view. While both assemblies have each scaffold that is declared chromosomal stand predominantly in a one-to-one monotonic relationship with the linkage map LG of the same number, the *Q. lobata* assembly (which did not use the linkage map for sequence construction) shows many fewer anomalies (despite being more distant to the map's cross). Points along very top and right edges of plots are those of pairings where chromosome and LG number disagree.

Supplementary Table 2. Statistics of *Q. lobata* and *Q. robur* assemblies vs. *Q. robur* x *Q. petraea* linkage map.

Q. lobata:

[A]	[B]	[C]	[D]	[E]	[F]	[G]	[H]	[I]
			uniquely				multiply	
**Linkage map linkage group**	# SNPs linkage map assigns to just this LG	filter [B] to those with ≥1 *Q. lobata* alignment	filter [C] to those with all alignments to same *Q. lobata* chrom./scaffold	filter [D] to those aligning to chr1..12	filter [E] to those aligning to same chr# as LG#	*[F] as % of [C]*	filter [C] to those with ≥1 alignment to same chr# as LG#	*[H] as % of [C]*
**LG I**	308	308	300	300	297	*96.4%*	303	*98.4%*
**LG II**	706	703	668	668	663	*94.3%*	697	*99.1%*
**LG III**	299	294	289	289	284	*96.6%*	289	*98.3%*
**LG IV**	227	227	211	210	207	*91.2%*	223	*98.2%*
**LG V**	298	297	271	271	259	*87.2%*	283	*95.3%*
**LG VI**	404	392	374	374	359	*91.6%*	376	*95.9%*
**LG VII**	325	322	301	301	296	*91.9%*	317	*98.4%*
**LG VIII**	433	423	405	405	395	*93.4%*	412	*97.4%*
**LG IX**	289	286	272	272	270	*94.4%*	284	*99.3%*
**LG X**	288	288	271	271	266	*92.4%*	283	*98.3%*
**LG XI**	294	293	280	280	275	*93.9%*	286	*97.6%*
**LG XII**	346	341	336	336	335	*98.2%*	340	*99.7%*
total	***4,217***	***4,174***	***3,978***	***3,977***	***3,906***	***93.6%***	***4,093***	***98.1%***

Q. robur:

[A]	[B]	[C]	[D]	[E]	[F]	[G]	[H]	[I]
			uniquely				multiply	
**Linkage map linkage group**	# SNPs linkage map assigns to just this LG	filter [B] to those with ≥1 *Q. robur* alignment	filter [C] to those with all alignments to same *Q. robur* chrom./scaffold	filter [D] to those aligning to chr1..12	filter [E] to those aligning to same chr# as LG#	*[F] as % of [C]*	filter [C] to those with ≥1 alignment to same chr# as LG#	*[H] as % of [C]*
**LG I**	308	305	299	289	242	*79.3%*	245	*80.3%*
**LG II**	706	696	688	662	569	*81.8%*	574	*82.5%*
**LG III**	299	297	287	273	208	*70.0%*	217	*73.1%*
**LG IV**	227	224	204	181	140	*62.5%*	149	*66.5%*
**LG V**	298	292	286	267	216	*74.0%*	218	*74.7%*
**LG VI**	404	400	390	374	312	*78.0%*	320	*80.0%*
**LG VII**	325	321	314	294	261	*81.3%*	267	*83.2%*
**LG VIII**	433	426	410	389	362	*85.0%*	375	*88.0%*
**LG IX**	289	286	275	256	210	*73.4%*	217	*75.9%*
**LG X**	288	284	272	264	220	*77.5%*	228	*80.3%*
**LG XI**	294	292	283	277	242	*82.9%*	247	*84.6%*
**LG XII**	346	342	339	322	253	*74.0%*	256	*74.9%*
total	***4,217***	***4,165***	***4,047***	***3,848***	***3,235***	***77.7%***	***3,313***	***79.5%***

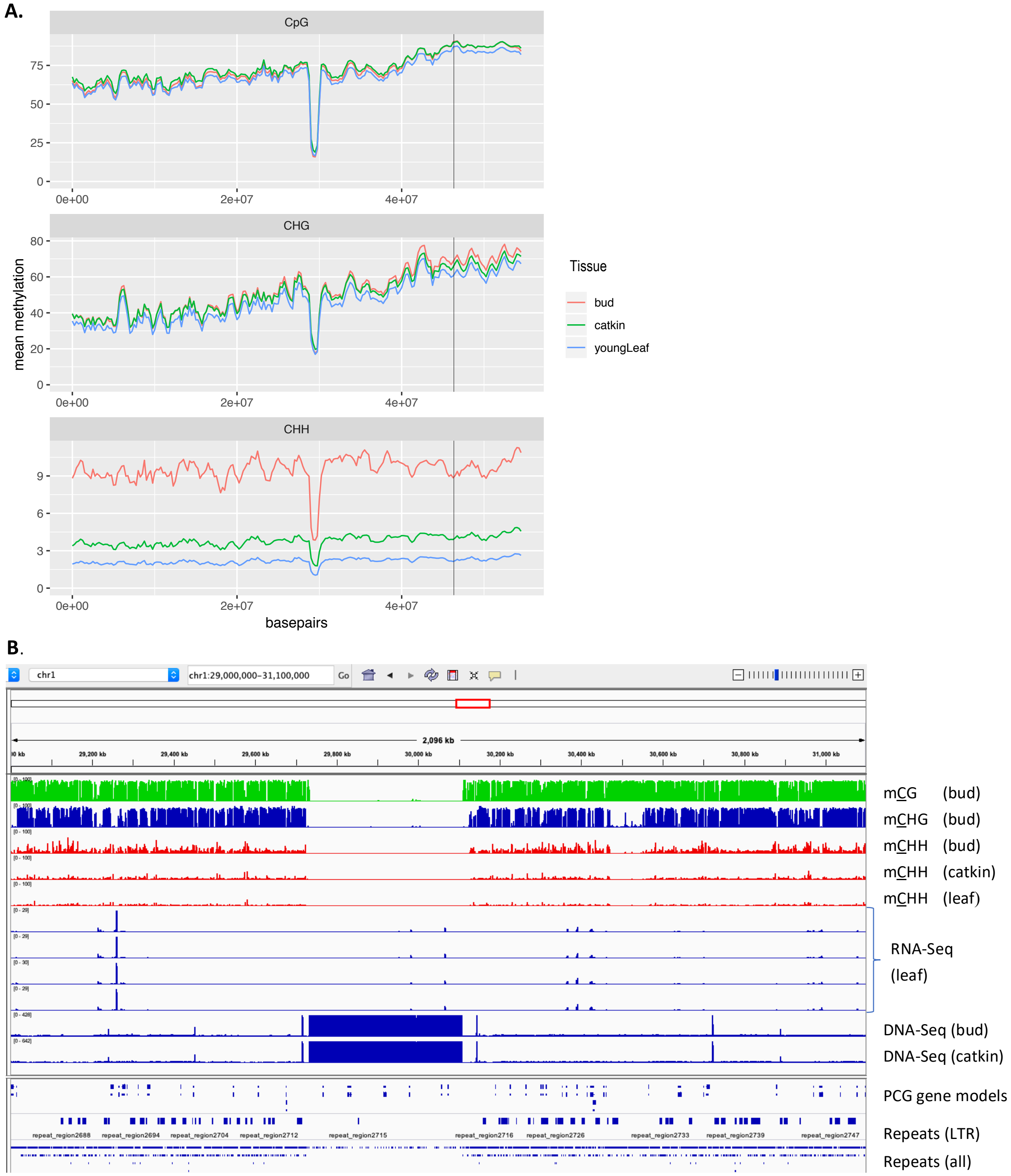

Supplementary Figure 3. Misassembled mitochondrial sequence in pre-final *Q. lobata* version 3.0 chromosome 1. **(A)** Mean methylation level (CG top, CHG middle, CHH bottom) for 1 Mbp windows every 250 kbp. **(B)** IGV genome browser screenshot showing selected methylation levels (top five tracks), Illumina RNA-Seq read coverage (next four tracks), coverage by Illumina genomic reads (next two tracks), gene annotations (next track), and repeats (bottom two tracks). The strong dip in methylation levels and large increase in genomic read coverage are coincident with a misassembly that placed a region of the mitochondrion sequence into near-final chromosome 1 at one-based inclusive–inclusive coordinate span 29,726,880 to 30,108,053 bp (on the '+' strand). In the final 3.0 assembly release, this coordinate span has been replaced with a gap (to not shift coordinates at this late stage).

Supplementary Note 3. Analysis of heterozygosity

For comparison with the *Q. robur* genome [23], we analyzed the heterozygosity of our Q. *lobata* genomes in two different ways. The first way was to compute Tajima's π [24] in non-overlapping 500 kbp windows across our 19 individuals. (For the second way, see **Supplementary Figure 5**.) To do this, we used Python function `windowed_diversity()` from the 'scikit-allel' 1.2.1 package [25] with `window_size` set to 500 kbp. This function computes Tajima's π by using allele frequencies
of SNPs to compute the total number of pairwise differences across all samples. The number of total differences is then divided by the total number of callable sites in each window. (Callable sites refers to the number of sites that passed
our filters in each window; see **Supplementary Note 4. Demographic analysis — Input to PSMC'** below.)

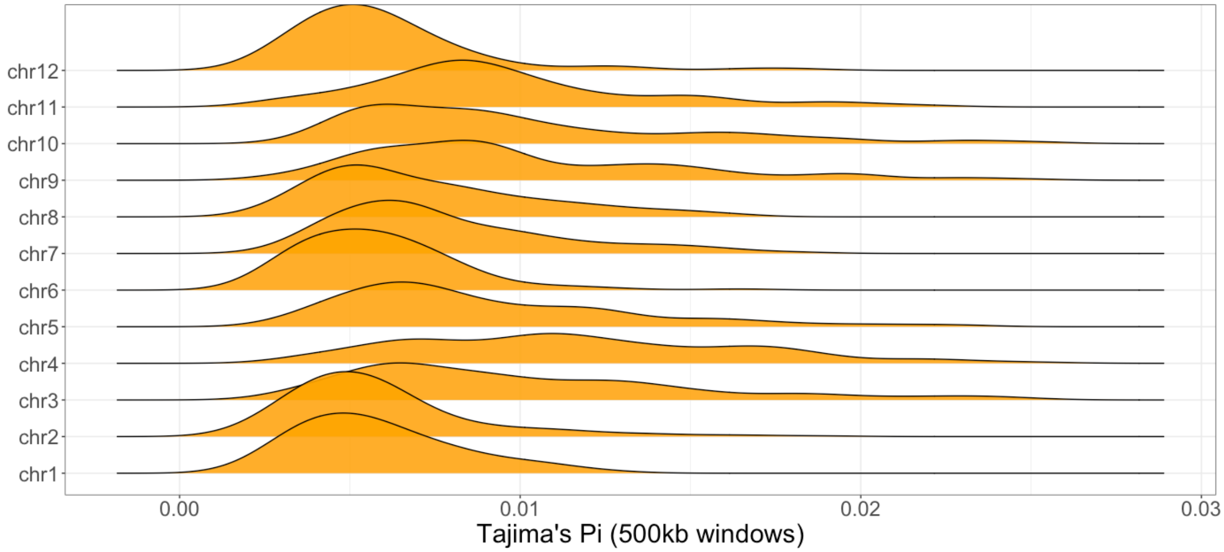

Chromosome	Number Of 500kb Windows	Total Callable Sites	Total Number of Variants	Mean Tajima's Pi
1	112	22,005,804	1,024,226	0.00580
2	209	41,297,702	1,746,992	0.00588
3	150	26,943,781	1,918,986	0.01004
4	196	30,807,490	2,422,621	0.01165
5	179	32,089,293	2,011,761	0.00895
6	109	22,882,763	1,074,710	0.00580
7	99	19,481,942	1,132,154	0.00780
8	131	26,525,174	1,307,858	0.00724
9	111	21,226,684	1,411,928	0.01000
10	133	23,245,880	1,600,529	0.00991
11	116	21,556,495	1,353,750	0.00954
12	87	19,006,813	847,498	0.00600

Supplementary Figure 4. Distribution of Tajima's π across the *Q. lobata* reference genome.
Top: per-chromosome distribution of Tajima's π [24] across our 19 individual diploid *Q. lobata* genomes. **Bottom:** per chromosome total number of 500 kbp windows, number of callable sites, and number of heterozygous positions for our samples.

The second way we summarized heterozygosity was by computing a heterozygosity rate. (For the first way, see **Supplementary Figure 4**.) In this analysis, we examined each of the 19 diploid genomes independently. For each of the 19 genomes, we considered non-overlapping windows of 500 kbp. In each window, we counted the total number of heterozygous sites divided by the number of callable sites to obtain the average number of heterozygous positions per callable base pair.

Both approaches gave similar results. Across chromosomes, both the heterozygosity rate and Tajima's π had similar magnitudes, ranging from ~0.005 to ~0.01. Likewise, both Tajima's π and the heterozygosity rate have similar distributions within a single chromosome.

For our PSMC' analysis, we computed the heterozygosity rate of the *Q. robur* reference genome, the *Q. lobata* reference genome, and one selected resequenced *Q. lobata* genome (QL.LAY.5.00F). For the *Q. lobata* reference genome, we found 1,716,263 heterozygous positions out of 349,858,917 sites (~0.50%), and for the *Q. robur* reference genome, we found 2,268,413 heterozygous positions out of 309,542,806 (~0.73%). For the *Q. lobata* resequenced genome, we limited our analysis to filtered sites in QL.LAY.5.00F shared by both QL.LAY.5.00F and the reference genome, and found 2,025,194 heterozygous positions out of 307,071,743 (~0.66%).

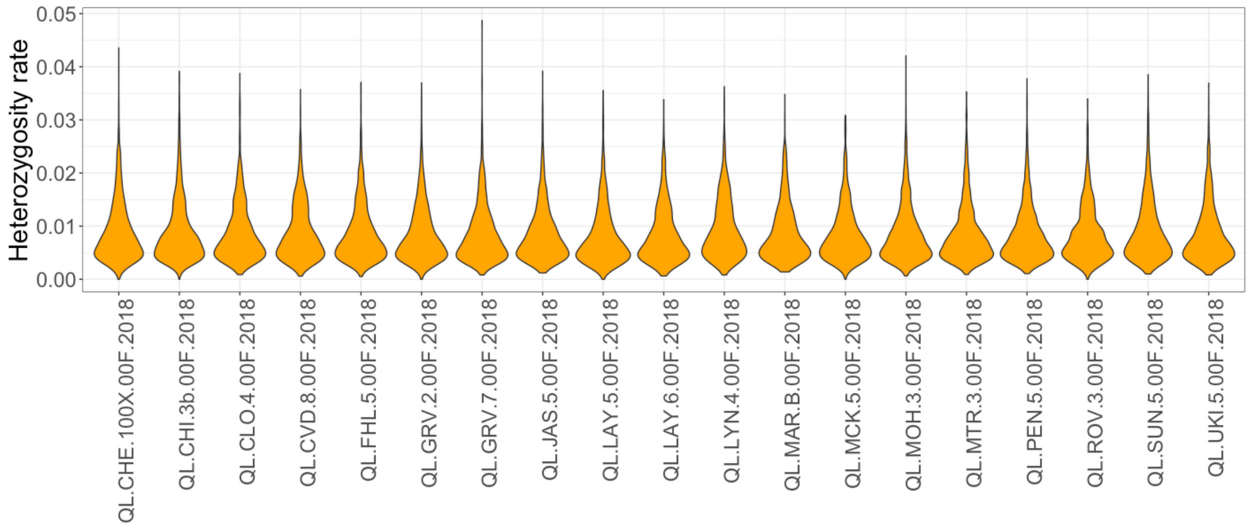

Chromosome	Number Of Windows	Total Callable Sites	Number of Heterozygous Positions	Heterozygosity
chr1	2,128.00	317,717,076.00	1,925,442.00	0.006060
chr2	3,971.00	585,250,875.00	3,355,901.00	0.005734
chr3	2,850.00	345,332,020.00	3,149,517.00	0.009120
chr4	3,724.00	381,396,603.00	4,295,624.00	0.011263
chr5	3,401.00	418,965,586.00	3,617,755.00	0.008635
chr6	2,071.00	331,233,288.00	1,925,282.00	0.005812
chr7	1,881.00	270,412,151.00	2,120,958.00	0.007843
chr8	2,489.00	373,392,027.00	2,652,599.00	0.007104
chr9	2,102.00	285,985,560.00	2,695,443.00	0.009425
chr10	2,527.00	303,504,786.00	2,694,611.00	0.008878
chr11	2,204.00	290,439,119.00	2,611,644.00	0.008992
chr12	1,653.00	272,784,844.00	1,602,538.00	0.005875

Supplementary Figure 5. Distribution of heterozygosity rate (heterozygosity per bp) across the *Q. lobata* genome. Top: distribution of heterozygosity rate across our 19 samples. **Bottom:** per chromosome total number of 500 kbp windows, callable sites, and number of heterozygous positions in our samples.

Supplementary Note 4. Demographic analysis

Inference of demographic history. We used the Pairwise Sequentially Markovian Coalescent (PSMC′) model to infer changes in effective population size in *Q. lobata* and *Q. robur* over time [26]. With a single diploid genome, PSMC′ utilizes the spatial distribution of heterozygous sites to first infer a distribution of times to the most recent common ancestor (TMRCA) across a whole genome. With this distribution of TMRCAs, PSMC′ can then estimate the effective population size N_e across the evolutionary history of a population using the inverse relationship between the coalescence rate and the effective population size [26]. Although the PSMC model was first developed to study the demographic history of humans [26], it has been used in the study of animals with distinct phylogenetic histories [27, 28, 29] as well as a variety of plants [30, 31, 32, 33, 34].

Input to PSMC′. For our analysis with PSMC′**,** we first masked out all genome gaps and repeats from the *Q. lobata* reference and resequenced genomes, and the *Q. robur* reference genome. Additionally, insertions and deletions were masked out. The mean depth DP for *Q. lobata* variants was 110 reads and the standard deviation was 60. The sequencing depth for *Q. robur* was slightly higher at 116 reads with a standard deviation of 53. Because the sequencing depth was similar between the two reference genomes, we used the same depth DP filters. We set the maximum DP filter for the non-variant sites in the reference genomes to be mean DP + 4·(standard deviation) = 350, and the minimum DP was set to be mean DP / 3 = 37. Variant sites in the reference genomes that satisfied any of our filter conditions (DP > 350, FS > 60, MQ < 40, QD < 2, SOR > 3, RPRS < –8, or MQRankSum < –12.5) were also excluded from analysis.

To ensure that the demographic history obtained for *Q. lobata* was not biased by mapping its sequencing reads back to its own assembled genome, we also ran PSMC′ on 19 additional resequenced *Q. lobata* genomes (**Supplementary Table 1**). We generated PSMC′ input using only callable sites, which we define as those having a minimum depth DP > 12 reads, mapping quality MQ > 20, and quality score QUAL > 10. The mean coverage of callable sites for all 19 resequenced samples was greater than the recommended mean genome coverage of ≥ 18x [35] in all but five samples. These five samples had mean coverages 14.9x–17.6x. Additionally, we removed all indels, variant sites immediately upstream and downstream of insertions and deletions, multiallelic sites, and repetitive sequences. 56.7% of the genome was removed due to repeat masking, which is greater than the ≤ 25% missing data threshold recommended by Nadachowska-Brzyska, Burri [35]. However, because of the overwhelming presence of transposable elements and repetitive sequences in *Q. lobata*, we masked out these sequences to avoid incorporating incorrectly called SNPs that may arise from alignment ambiguities. PSMC′ was run with default settings except for the maximum number of iterations set to 200. Because PSMC′ was designed to be used on human genomes, it begins its expectation maximization algorithm to infer the ratio of recombination and mutation rates at a value of 0.25. Although starting at this ratio may be appropriate for humans, it is currently unclear how the coupling of long lifespan [36] and non-human reproductive biology (for example, possible somatic generation of diversity being passed onto the next generation [23]) in oaks contributes to this ratio in *Q. lobata*. By allowing for more iterations of the expectation maximization algorithm, we allow for a larger space of recombination to mutation rate ratios to be explored. Qualitatively, we did not see large differences in the demographic trajectory depending on the maximum iteration limit except in the ancient time steps.

Estimation of neutral mutation rate. Neutral mutation rates for *Q. lobata* and *Q. robur* are needed to scale PSMC′ output into units of effective population size and years–ago. Unfortunately, published estimates of these quantities are not available. Thus, we estimated the neutral mutation rate from sequence divergence. Assuming the divergence between *Q. lobata* and *Q. robur* is much greater than the expected levels of polymorphism in the ancestral species, we estimated a mutation rate using the relationship between divergence and split time [37]. To compute a mutation rate, we used MUMMER [38] to align the *Q. lobata* version 3.0 reference genome and the *Q. robur* reference genome to each other. We calculated divergence by counting the number of positions that differ between the aligned reference genomes that have a 1–to–1 mapping, and divided this by the total number of aligned nucleotides. In this computation, we masked out repeats and genome gaps in both genomes and found 241,827,461 matching nucleotides between *Q. robur* and *Q. lobata* and 4,555,467 mismatching nucleotides. Then, using an estimated split time of 35 million years and a generation time for *Q. robur* of 30 years and for *Q. lobata* of 50 years, we estimated a mutation rate of $1.01 \cdot x\ 10^{-8}$ bp per generation. The generation time for *Q. robur* was based on estimates of other temperate tree species, such as walnut [34], and the generation time for *Q. lobata* was

set at 50 years because of *Q. lobata* life history traits vs. *Q. robur* (maximum life span ≈1,000 years vs. 600–800, larger acorn crop sizes, and older ages of standing tree populations).

Accurate estimates of mutation rates are difficult to experimentally measure in woody plants [39]. Additionally, it is difficult to estimate accurate neutral mutation rates for these organisms with sequence divergence. It is possible that our neutral mutation estimates are inaccurate due to factors that are not constant over time, such as differences in DNA-repair mechanisms, generation times, metabolic rates, inability to incorporate uncertainty in fossil identification, uncertainty in estimates of fossil ages, and the large variance around the substitution rate for any given time period [40]. However, while different estimates of the mutation rate and generation time scale axes, they do not change the overall shape and pattern of the inferred effective population size trajectory (e.g., see **Supplementary Figure 6**). Therefore, our qualitative conclusions about the demographic history of *Q. lobata* should be relatively unaffected by these possible biases.

Simulations in 'msprime'. To qualitatively assess whether PSMC′ can accurately infer population size changes similar to those for oak trees, we used coalescent simulations implemented in 'msprime' to simulate data under our inferred demographic models for each of the three types of genomes. For the *Q. lobata* reference genome, *Q. robur* reference genome, and the chosen *Q. lobata* resequenced genome, the inferred demographic history from PSMC′ is defined by 40 points. We scaled these 40 points into effective population size (N_e) and number of generations before the present (γ) by adopting the mutation rate μ = 1.01 x 10^{-8} bp per generation we estimated earlier above and applying the formulas $N_e = (1/\lambda)/(2\mu)$ and $\gamma = \psi/\mu$, where λ = PSMC′-inferred `Lambda_00` and ψ = PSMC′-inferred left time boundary.

With 40 pairs of N_e and γ, we generated a corresponding 'msprime' function. Each change in N_e was done instantaneously with a growth rate of zero. To generate one replicate of a simulated genome, we simulated twelve independent replicates of chromosomes of fixed length 29 Mbp. Recombination was taken as a uniform rate of 2 x 10^{-8} bp per generation over each simulated chromosome, and mutation was also taken uniform at 1.01 x 10^{-8} bp per generation. After each simulation completed, we used 'msprime' to output each simulated diploid chromosome in VCF file format. We then generated the input to PSMC′ (a "multihetsep" file) from such a VCF custom script (https://github.com/jessegarcia562/psmc2msprime) [41]. With twelve simulated chromosomes and their corresponding multihetsep files, we then utilized PSMC′ with default settings except 200 iterations to infer the demographic history of one simulated genome.

For each genome type (*Q. lobata* reference, *Q. lobata* resequenced, and *Q. robur* reference), we performed the above-described simulation and PSMC′ inference 10 times. These analyses provided 10 simulated genomes for each genome type, and therefore 10 PSMC′-inferred demographic histories for each genome type. For each of these 30 total simulated genomes, we computed heterozygosity by dividing the total number of heterozygous sites in the simulated genome by the total simulated genome length (12 x 29 Mbp = 348 Mbp).

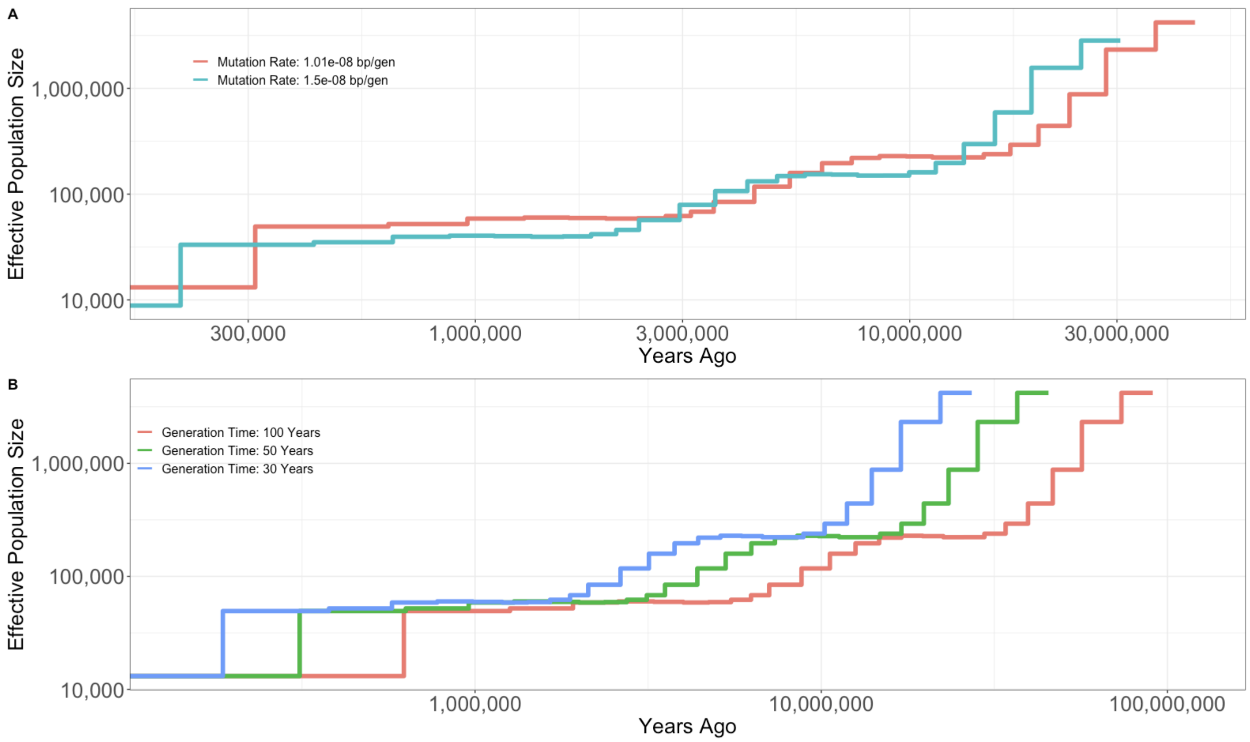

Supplementary Figure 6. PSMC′ inference on the *Q. lobata* reference genome when using different generation times and mutation rates. (A) Assuming a generation time of 50 years, different estimates of the mutation rate would move the demographic trajectory along both the *y*-axis and *x*-axis. However, different estimates would not change the overall shape of the curve. The mutation rate 1.01 x 10^{-8} bp per generation was estimated from the divergence between the *Q. lobata* and *Q. robur* reference genomes (see **Estimation of neutral mutation rate** earlier). The 1.5 x 10^{-8} bp per generation mutation rate illustrated here was chosen arbitrarily to illustrate the effect changing mutation rate has on effective population size. (**B**) Assuming a mutation rate of 1.01 x 10^{-8} bp per generation, different estimates of generation time would only move the demographic trajectory along the *x*-axis because larger generation times would push the estimates farther into the past.

Identifying a trim point. While PSMC′ can infer complex population size-change models, these models may not accurately predict simple empirical summary statistics, such as the genome-wide distribution of heterozygosity [42]. It is unclear precisely why this problem occurs, but one hypothesis is that methods such as PSMC′ might overestimate the ancestral size of a population[42]. In order to present a demographic history that accurately predicts both the empirical genome-wide rate of heterozygosity and the empirical genome-wide distribution of TMRCA, we attempted to correct for the possible overestimation of the ancestral size from the initial full model (**Supplementary Figure 7**). As the demographic trajectories for each genome type (when moving forward in time) all appeared to be monotonically decreasing in our ancient time steps, we decided to use each predicted time step as a possible ancient ancestral population size. Specifically, we had in total 40 inferred pairs of N_e and γ that defined the demographic trajectory for each genome type. From the original 40 pairs of points PSMC′ inferred, we created 39 new demographic trajectories by iteratively removing the most ancient (largest in magnitude γ) remaining time step. For example, while 40 points describe the full PSMC′ demographic model, after removing the most ancient time step, we can generate a new demographic trajectory that is instead defined by only 39 points. This iterative process of generating new demographic trajectories results in one full model (with all 40 N_e and γ pairs) inferred by PSMC′, and 39 trimmed models (of 39, 38, …, 1 point[s]). Importantly, the population size remains at the same size as the last point defining the demographic history for an infinite amount of time going

back into the past. Thus, this trimming strategy resulted in changing the ancestral population sizes of the PSMC'-inferred demographic model.

Following the methods described earlier under **Simulations in 'msprime'**, we simulated 1 Mb of sequence under each of our 40 models for each genome type, and computed the predicted heterozygosity of each model (**Supplementary Figure 8**). We then visually compared the fit of the simulated distribution of heterozygosity to the values observed empirically. Our best models for the *Q. lobata* reference genome, *Q. robur* reference genome, and *Q. lobata* resequenced genome were defined by 32, 32, and 28 points, respectively, although none of our 40 models could precisely predict the exact genome-wide heterozygosity for the respective genome type. This result suggests that the true demographic history is likely more complex and is not entirely captured with these size change models. Nevertheless, trimming allows the demographic models presented in publication **Figure 2** to more closely match the heterozygosity in the observed data than what the untrimmed model predicted (**Supplementary Figures 9** and **10**).

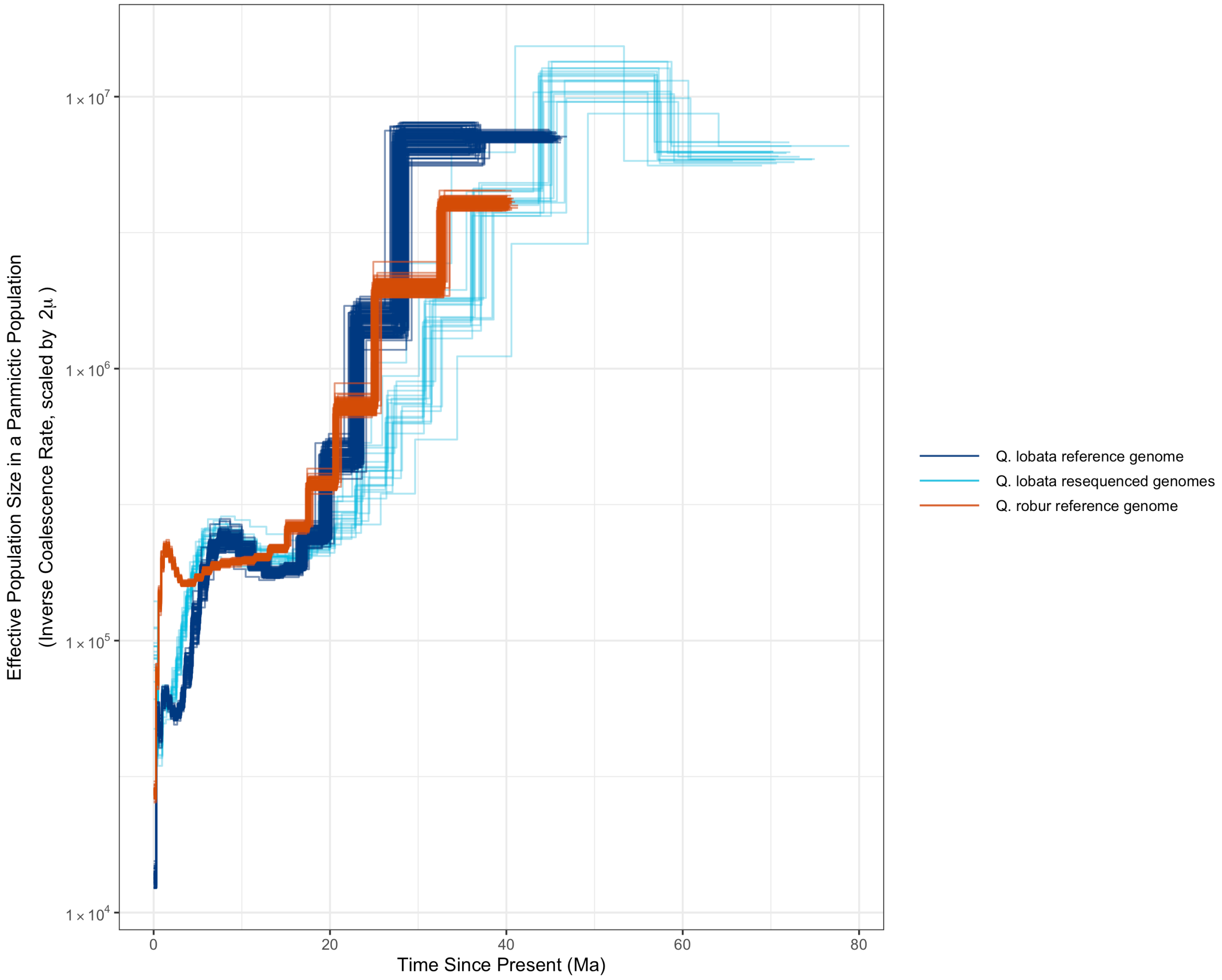

Supplementary Figure 7. Full demographic models inferred by PSMC'. This figure differs from publication **Figure 2C** in that none of these models have their ancestral population sizes trimmed to fit the empirical rate of heterozygosity observed; visualized here is the unprocessed raw output from PSMC' scaled by the estimated mutation rate and generation time for each species.

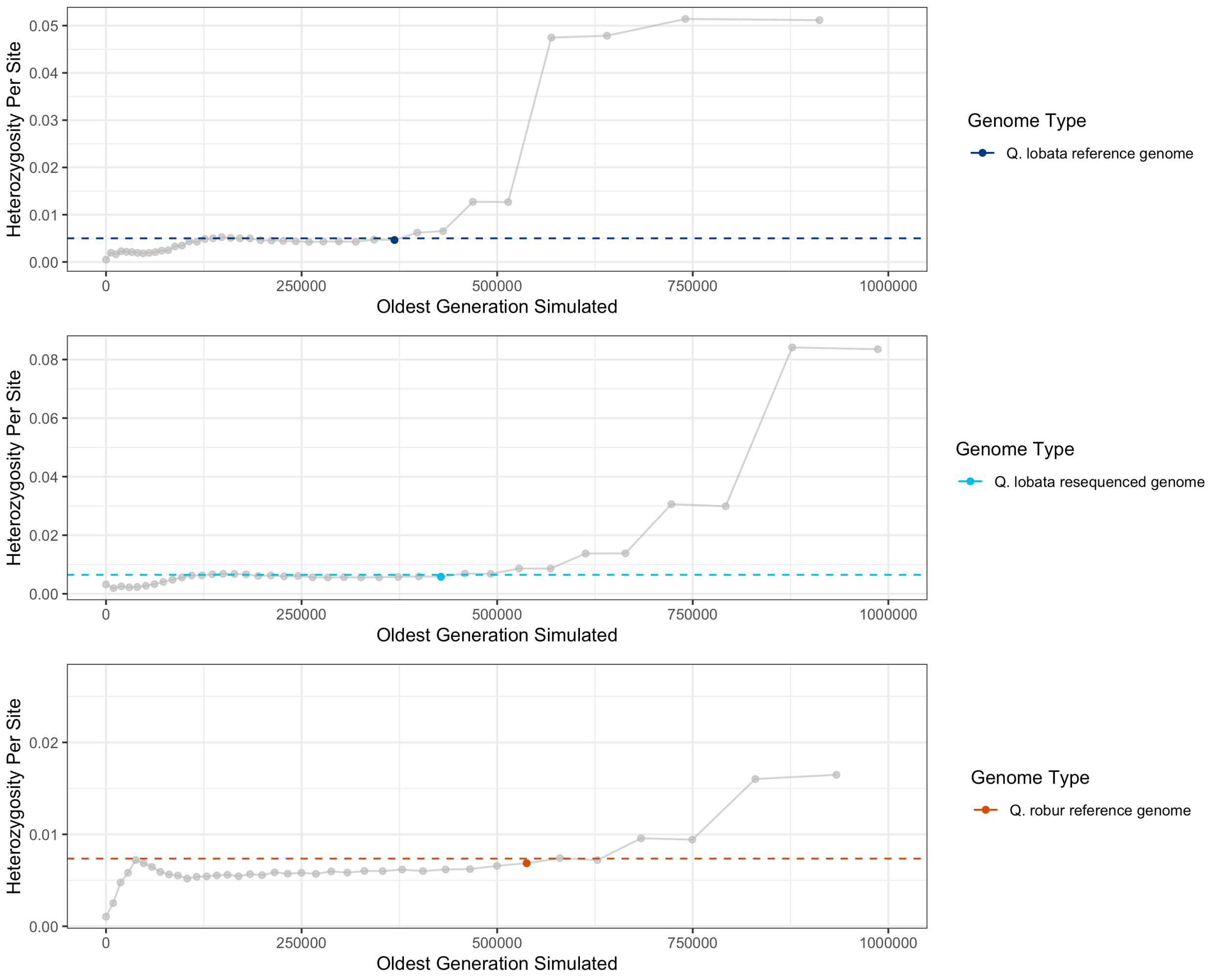

Supplementary Figure 8. Predicted heterozygosity for 1 Mbp regions for all trim possibilities for each genome type. Dashed lines are the respective empirical heterozygosity rate for each type. Highlighted points represent the models that we chose to represent each genome: that with 32 points for both the *Q. lobata* and *Q. robur* reference genomes, and that with 28 points for the *Q. lobata* resequenced genome.

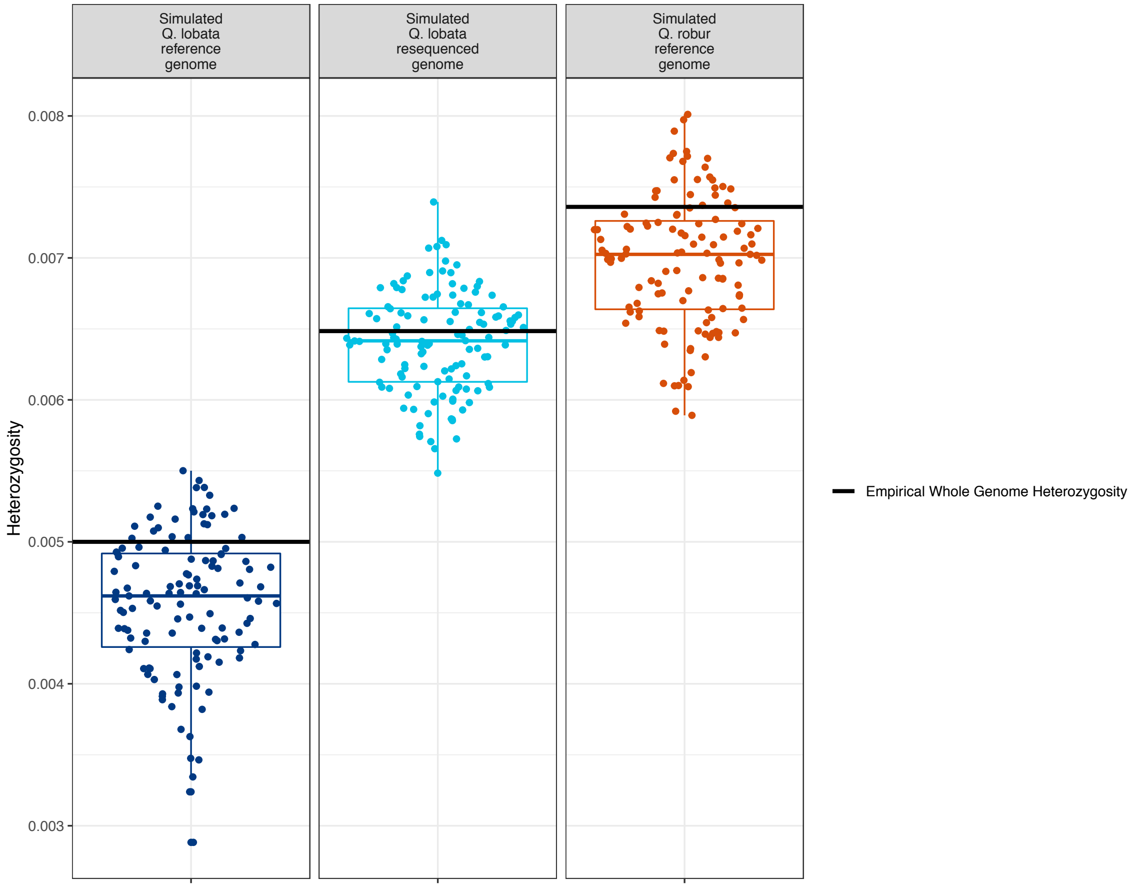

Supplementary Figure 9. Predicted heterozygosity for 120 simulated 1 Mbp regions of the best fitting models for each genome type. Each point is the heterozygosity of a simulation replicate. Each box shows interquartile range (IQR), from first (Q1) to third (Q3) quartile; box middle bar is at the median. Lower whisker extends to smallest point ≥ Q1 – 1.5·IQR, upper whisker to largest ≤ Q3 + 1.5·IQR, Black horizontal lines show empirical observed whole genome heterozygosity for each genome type.

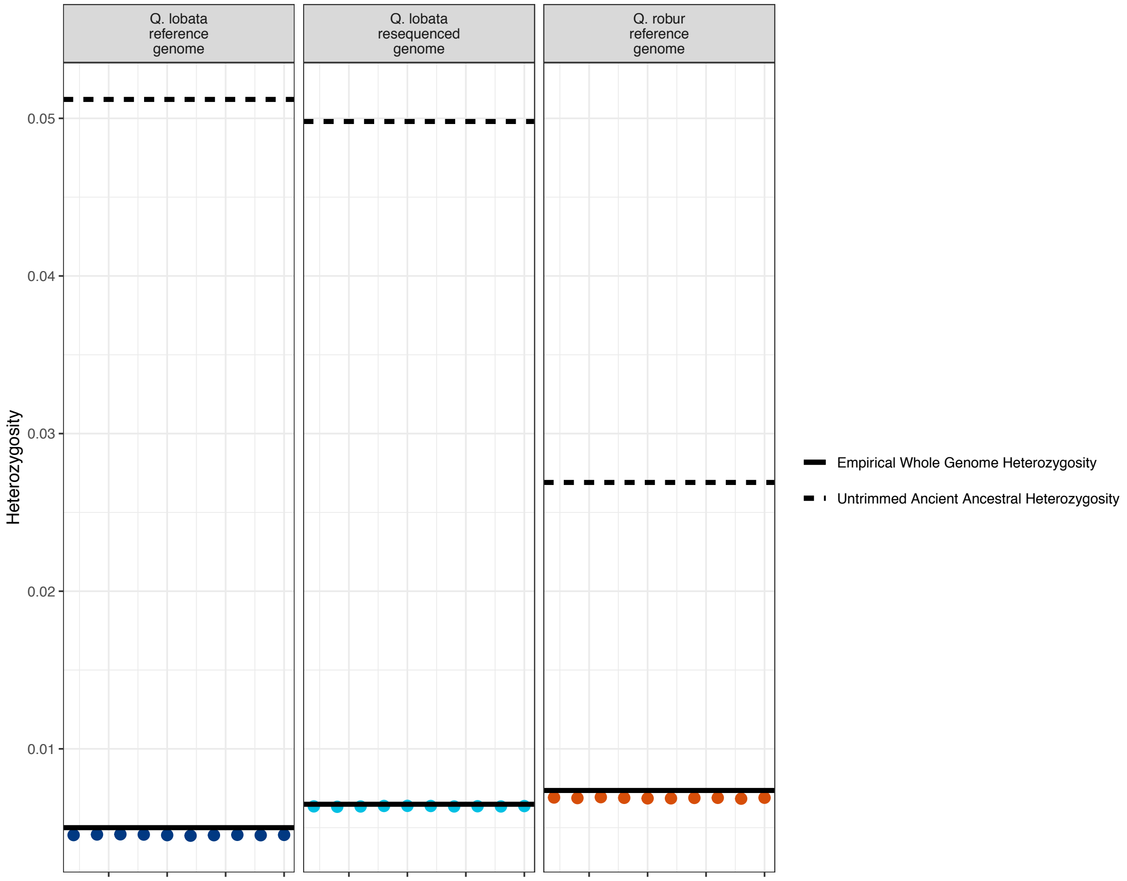

Supplementary Figure 10. Predicted heterozygosity of full untrimmed PSMC' models compared to that of best-fitting trimmed ancestral models. Best fitting trimmed models are models which have reduced ancestral population sizes (relative to the full untrimmed PSMC' models) that — when simulated with 'msprime' — fit empirical whole genome heterozygosity. The trimmed models with 32, 28, and 32 points were chosen for the *Q. lobata* reference genome, the *Q. lobata* resequenced genome, and the *Q. robur* reference genome, respectively. Plotted dots show heterozygosity of 10 simulated genomes under the chosen trimmed ancestral model.

Supplementary Note 5. Assessment of amino acid diversity in the large DUF247 block on chromosome 4

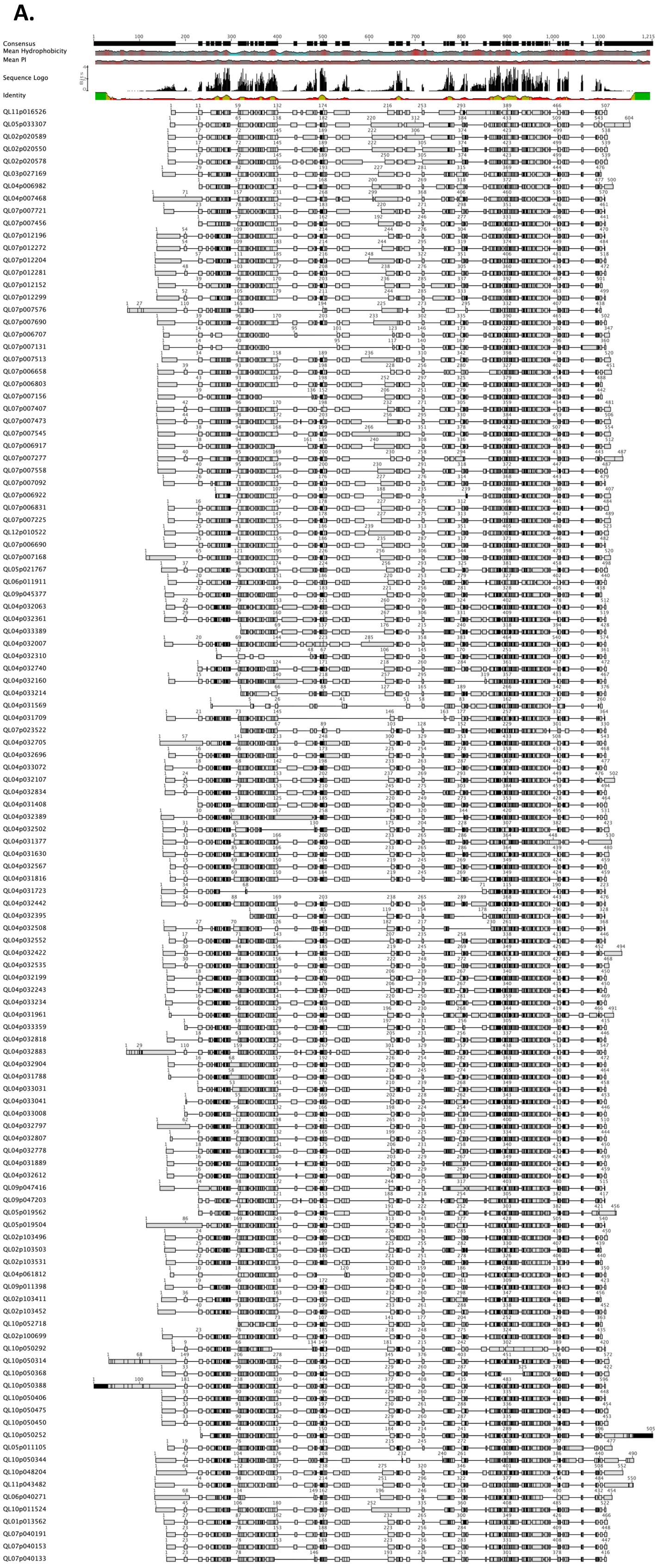

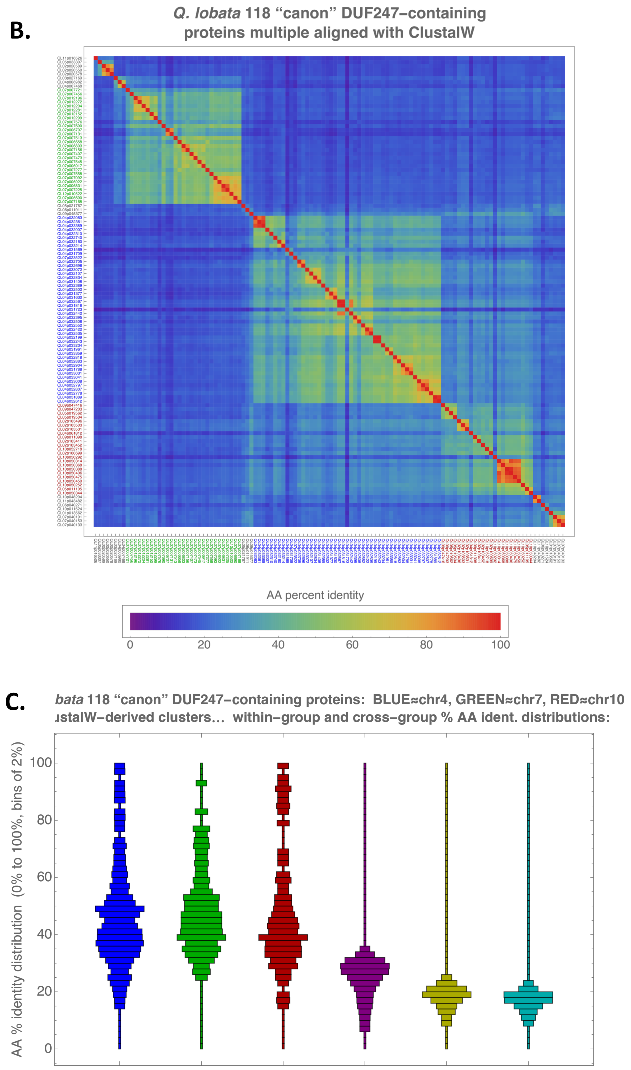

Supplementary Figure 11. DUF247 PCG amino acid diversity. Amino acid translations of the 161 PCG models with ≥ 1 DUF247 Pfam hit were multiply aligned with ClustalW [43], and 43 outliers with respect to similarity were removed (some were partial, some unusually long, ...), leaving 118 "canons". **(A)** ClustalW re-alignment of the 118, visualized with Geneious [44]. **(B)** A Jukes-Cantor tree with 100 bootstraps suggests three clusters, labeled in this % identity heatmap in blue (47 members ≈ the large DUF247 block of chr. 4), green (29 ≈ chr. 7 cluster on DUF247), and red (23 scattered across multiple chrs., but with ≈half on chr. 10). Striking is the diversity of similarities (even though all match Pfam's DUF247 HMM model), from near 100% down all the way to the 20% ©dentity "twilight zone" and below. (**C**) Within- (blue = blue–blue, green = green–green, red = red–red) and cross-cluster (magenta = blue–red, yellow = green–red, cyan = blue–green) histograms of % identity provide another view of DUF247 sequence diversity.

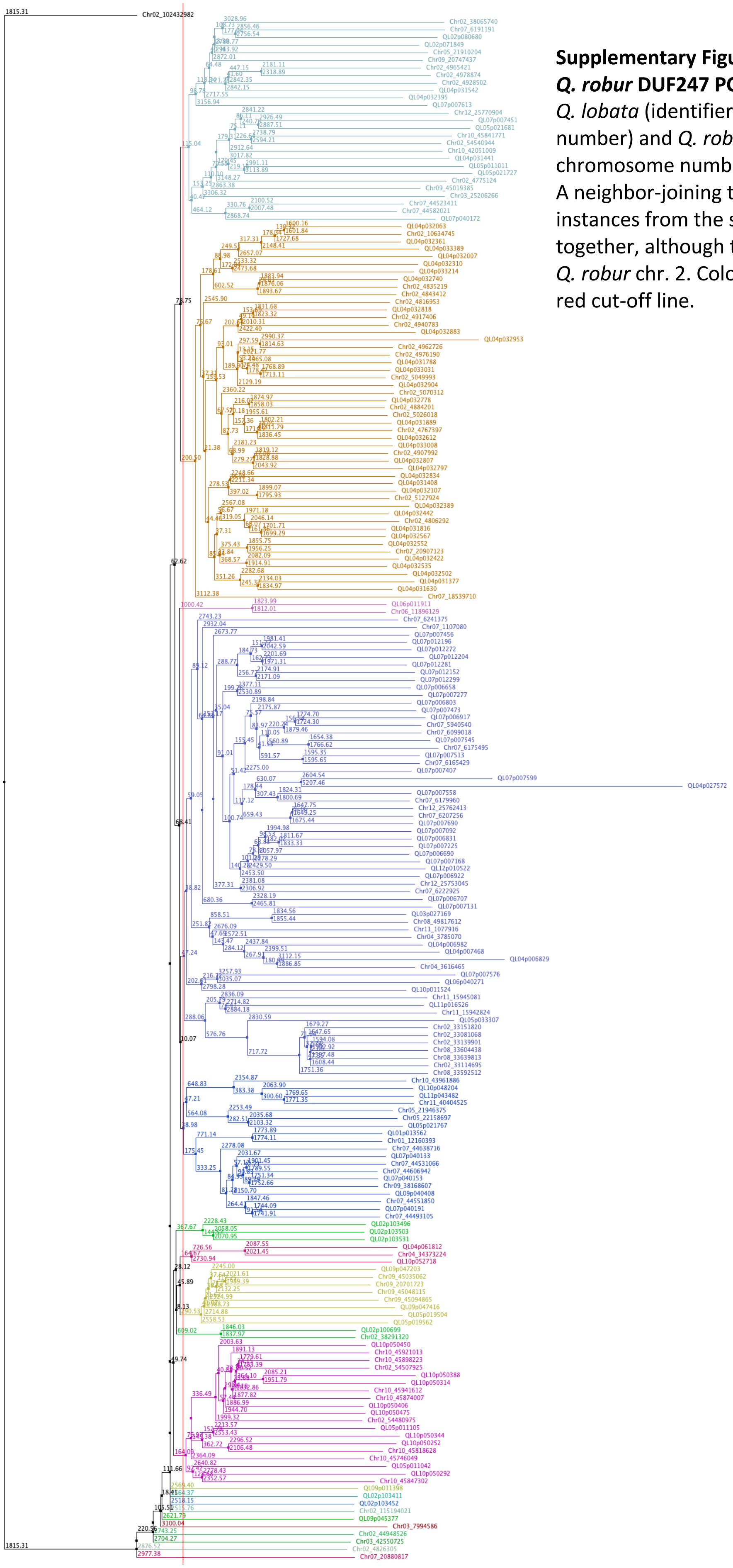

Supplementary Figure 12. Phylogeny of *Q. lobata* and *Q. robur* DUF247 PCGs. Translated gene models from *Q. lobata* (identifiers *QL##p…* where *##* = chromosome number) and *Q. robur* (identifiers *Chr##_…* where *##* = chromosome number) that contain DUF247 were identified. A neighbor-joining tree of the amino acid sequences shows instances from the same chromosome tend to cluster together, although the *Q. lobata* chr. 4 cluster is found on *Q. robur* chr. 2. Colors show clades defined by the vertical red cut-off line.

Supplementary Note 6. Repetitive sequences

Additional findings and methods

A database generated by RepeatModeler consists of repeat "families", each given by a consensus nucleotide sequence derived from a multiple alignment of some high-copy homologous regions of the genome, and many families are automatically placed into a particular major (e.g., "Long Terminal Repeat" — LTR) and minor class (e.g., "Gypsy") by a subcomponent (RepeatClassifier) aligning against known repeats (with variable accuracy; we did not revise by manual curation). The consensuses are not always full length for their class or irredundant by close sequence similarity; for *Q. lobata*, we applied PSI-CD-HIT 4.7 to cluster at 45% nucleotide identity (the level where, as the threshold is lowered, intracluster similarities stop falling in frequency and begin rising) and chose a canonical rotation/strand for tandem repeat units so as to cluster families into "superfamilies" (SFs), generally assigning to each SF the major/minor class of the longest member family that was not unknown (if any). Annotated intervals for a SF are the nucleotide-level union of all intervals for its member families, and SFs are given "s1RF####" accession numbers (roughly by descending mass of nucleotides masked). We also applied LTRharvest and LTRdigest from GenomeTools 1.5.9 to specifically target the prevalent LTRs, which identified 28k instances covering a total of 184 Mbp (only slightly more than the 179 Mbp RepeatClassifier declared as LTR).

Examination of instances of individual SFs identified s1RF1096 as the telomeric tandem repeat (the common plant unit $(AAACCCT)_n$ when at 5′ ends, and mostly restricted in occurrence to edges of assembled chromosomes), as well as 148 bp complex tandem unit s1RF0004 (GCTCATGGGC CCCCGACCCG AGTTAGAAAA TTCAAAAAAT AAATGCAAAA AAATTCTAAA AATTAAAAAA CATCATCCAG GCTTCATTTC AAGACGAAAA CGGGTCAGAG ACAGGCCGAA AAATAGAGAA CAAAAATTTC ATTCCTAA) which exists in relatively large total quantity (≈3 Mbp) and is essentially restricted to exactly one locus per chromosome, strongly suggesting this identifies centromeres, with s1RF0004 reminiscent of, e.g., CEN180 of *Arabidopsis* [45]. Over the project, further evidence (gene density profiles and DNA methylation patterns) accumulated additional support that this does indeed mark centromeres. Approximate intervals spanning centromeres are given in the table below. Clustering of chromosomal distributions of SFs (**Supplementary Figure 13**) indicated that the main chromosome-scale distributional features of repeats are associated with distance to centromeres. The distributions are well-summarized per SF by average distance of the SF members to the centromeres (**Supplementary Figure 14**), and were used to identify SFs with unusual preference for or avoidance of the centromeres (publication Figure 3C–D). The SFs so-identified have striking distributional concentrations that are nearly completely diluted away if only the distribution of all repeats taken together is examined (which is nearly uniform across chromosomes). These concentrations mainly fall to individual SFs and are not strongly associated with entire major repeat classes. Exonic density from protein-coding genes also shows a notable gradient, being lower near centromeres.

**Chromosome**	**From**	**To**
1	45,377,794	47,303,741
2	42,723,096	45,702,847
3	46,505,435	47,582,132
4	57,272,007	58,869,737
5	37,258,031	38,038,545
6	25,012,718	26,574,917
7	18,052,972	19,452,171
8	24,326,695	25,399,835
9	36,750,921	36,955,183
10	36,598,025	38,256,999
11	30,653,296	31,212,808
12	19,137,856	20,625,243

(1-based inclusive–inclusive intervals on '+' strands for approximate intervals that contain centromeres)

Identification of large arrays of rDNA was attempted. *In silico* isolation of a canonical rDNA tandem unit* was complicated by the unit's borders incorporating a complex multi-scale tandem repeat (with $(GGCCTT)_n$ as short bottom-level unit), with individual copies of the rDNA unit highly diverging there. Alignments of Illumina short reads to a 9.1 kbp consensus (that included full 18S+28S) vs. generic homo- and heterozygous regions of the

nuclear genome suggest a total of ≈8 Mbp of rDNA from ≈870x copies of the rDNA unit, similar to the ≈750x of *Arabidopsis* chromosomes 2+4 and the ≈840x of *Chlamydomonas* chromosomes 1+7+15 [46]. The clean final assembly, however, contains only ≈0.3 Mbp aligning to the 9.1 kbp consensus. Thus (as is common), the assembly highly under-represents rDNA. The highest concentration found is on chr. 1 (the only acrocentric chromosome) in the short right arm (where LG1 is constant in a large block, a phenomenon that occurs only once across all LGs).

**Best-guess canonical rDNA tandem unit* — starts with a 2,943 bp spacer, with first ≈1.6 kbp repetitive; NCBI web BLASTN finds *Quercus robur* EF208967.1 for this, and in fact a note for that says “derived from 8Kb rDNA unit”:

GGCCTTGGCTGCGTGCCTTGGCCTTGGCAGCATGCCTTGGCCTTGGCTGCCTTGGCCTTGGCTACGTGCCTTGGCCTTGGCAGCATGCCTTGGGGGCCTGCCTTGGCCTTGGCTGCATGCCTTGGCCTTGGCTGCATGGGCCTTGGCTGCGTGCCATGGGGGGCAGCCCC
TGGCCTTGGCAGCATGCATGCCATGGCCTTGGGCCTTGGCCGCGTGCCTTGGCCTTGGCTGCTGCCTTGCCCACAAATTTCGAGTGATTTCCCAATAAATGAGGGTTTTTTGGAAAAGGAGGTTATTTTCCCCCCAAAGAGGCAGGCGTTGGGCATGGCAGGGTGCCCAG
GGGCATGCCCGCATGCACGCCACGCCGCATCGCCCCGCATCGTCCCGCACTCCGGCAAAATTGGCTCGTGCCTTTTCCCCTTTTTGTGTTCCTAAATTCAGTCCATGATTTTAGAGGACGTTTCCAACAAGCGGTTCGGCGTTCCGAGCAGTTTTGAATTTTTTATGATT
TTTCCTATTTTCTGGATTCCGAAAATCATAAAAAATAAAATATGTTGAATCTGGCCACCAAATTTTGACAGGTTGAAGGTTAGATTTTTCTTAGCATGTGTACAAAAAATCAGGGCAAGACTCCAAGAATTGCTCCAAAAAAGACCCATTATTCCTCCTCAACAAATCAA
TGTTTCCTCGTGCCAGTGTGGATTTTTTCCTAAGCGCTCTTTAGGGGGGGAAGAGTTGGAGGTGTCCGAAGAGGCTATCTAGGCTTGGCAGCAGTAGCCCCCCCAAGTGCTGCCACGGCCTTGTAGGGGTCCCAAGGGCATGCCACGGCCTTGGCATCCCACCCACGGGG
CATGCCACGCCCTCGCCCACGGGGCATGCTAGGGCCTTGCTGCTGCCTGCGCCCAGGGGCATGCCAGCGTGCCCACCTGCCTGCACCCAGGGGCATGCCAGCGTGCCCACGCAAGCATGCCATGGCCTTGGCTGCGTGCCTTGGCCTTGGCAGCATGCACGCAAGCAAGC
ATGCATGCCTTGGCCTTCGCTGCCTGCCCATGGGGGCTGCCTTGGCCTTGGCTGCTGCATGCCCATAGGGGGCTGCCTTGGCCTTGGCTGCATGCCTTGGCCTTGGCCTTGGATGCATGCCTTGCCCATGGCCTTGGCCACATATTTGGAGTGATTTCCTAAAAATGAGG
GTTTTTTGGAAAATGAGGTTATTTCCCCACGAGCAGGCAGGCAGTGGGCATCCCAGGGGCATGCCCGCGGGCACGCCGTGCCGCATCGCCCCGCATCGTCCCGCACTCCGGAAAAATTGGCTCGTGCCTTTTTCCATTTTTGTGTCCCTAAATTCATTCCATGATTTTAG
AGGAGGATTCCAGCAAGCGGTTCGGCGTTCCGAGCGTTTTTGAATTTTTTATGATTTTTCCCATTTTCCGGCTTCCTAAAATCGTAAAAAATAAAATATGTTGAATCTGGCCTCCATATTTTGACAGGTTGAAGGTTGGATTTTTCTTAGCATGTGTGCAAAAAATCAGG
GCAAGACTCCAAGAATTGCTCCGAAAATGACCCATTATTCCTCCTCAACAAATCAATGTTTCCTCGTGCCAGAGCGGATTTTTTCCTAAGGGCTCTTTAGGGGGGAGGAGGTATTCGGCGATGCACAGGGGCGACCCCTCTTGAGCTTGGCCACGGGTAGGCATGCTCAT
GACGAGCCCTCAGGCACCCAACCCCGCGTGCTCCATGGCGCGAGGTGGGCCCTAAATGCATCGCCGGGGTGGACCCAGGTTGGCATTTCACGTGTACGTGGTATAGCTGCGGCGCGCTCTTGCAAGGCGGACGGTGTTAGTTTCACCCATTGCCACGCAGAGCAAGCCTA
AGGTGATGATCAAACGCATTGTGAAAGCTTTCTCTGGTGCCACTTTTTCCTCGAGGCCACACCCACCCGTGCCCAGTGGCGGGGGGTCGATGGTCTCAGTAGCCGCGTGGTGGGGGGATGCATGCATTCTTGGTTTAGCTTGGTCACGCTATCGCAACGCGGACGGTGTT
AGTTTCACCCATTGCTGCGCGAAGCAAGTTCCATGCGACTATCGGGCTTGTGTGTGTGTCTTTCTTACTACGCGGTTGCGTGGTAGCAGTGGCGGCGGCGGCGGCGGCGGCGACGGCGACGACGGCAACGACAGCAGTGTCCCTCGATGTCCCTCGATGTCCCTTGTAGTTC
CTGTGTGTGGTTGGTGAGCCATGCAAGCTTGGTATAGCTTGGGCACGCTCTCGCAAAGCGGACGGTGTTCGTTTCACCCATTGCTGCGCGAAGCAAGTCCCACGGCGACCATCGGGCCTCTGCGCGGCGACGACCCATCCTTGCAGGCACGTGGCTTAGTAGTGTTGTCC
TTCACGCCCAGCGGTGCGGACTCGGCGCCACTCGATGCTTCCTCATGCACGGCAGGCCTTGCGGCGTGTCGTTGTGGGGTACGTTTGGTGGTGTTGCGGTCTAGTGTACGTGATAGCGTGTGAGTGGTGGCAGGGTTGCATGGCTTGGCAGGCTCCGTGCTCGCGCATCG
AACTGTCCGGCGTGCTCCCAATCAGCGTTGTTCCTAGCGTCGCTCGGACGCAATTCGGGTCCCTGTGTTGCATACCTGCCTCTAAGGCACTCGTCCCTCTAGTTGATTCGTTCCTAGTCGACGCTCCTCACGGGGCGTCGGCAGGACCTCGAAGCCGTCCTCGTGTCCCA
CGCGTGCCTCGCGGCCTCCGCGTTGCCGATGTGGACCACGTGGGCGTGCTCGTGGCCTCGGATGCAGAACACCATGTGGGTTTGGGGCCTTCGGCCCCCTTTGCCAACGTACCTAGCGAGCGTCATCGCTCTGCCCCGCACGATCGCCGTGCTTGTCCGTGCCCTTCCTT
GCCCTCGGGCGAGCCAGGGCCTCCGGGCGGCGCCGGCATCGACGAGGAATGCT

Next is 1,808 bp 18S (per RNAmmer):

ACCTGGTTGATCCTGCCAGTAGTCATATGCTTGTCTCAAAGATTAAGCCATGCATGTGTAAGTATGAACTAATTCAGACTGTGAAACTGCGAATGGCTCATTAAATCAGTTATAGTTTGTTTGATGGTACCTGCTACTCGGATAACCGTAGTAATTCTAGAGCTAATACG
TGCAACAAACCCCGACTTCTGGAAGGGATGCATTTATTAGATAAAAGGCCGACGCGGGCTCTGCTCGCTGCTCTGCTGATTCATGATAACTCGACGGATCGCACGGCCATCGTGCCGGCGACGCATCATTCAAATTTCTGCCCTATCAACTTTCGATGGTAGGATAGTGG
CCTACTATGGTGGTGACGGGTGACGGAGAATTAGGGTTCGATTCCGGAGAGGGAGCCTGAGAAACGGCTACCACATCCAAGGAAGGCAGCAGGCGCGCAAATTACCCAATCCTGACACGGGGAGGTAGTGACAATAAATAACAATACCGGGCTCTCACGAGTCTGGTAAT
TGGAATGAGTACAATCTAAATCCCTTAACGAGGATCCATTGGAGGGCAAGTCTGGTGCCAGCAGCCGCGGTAATTCCAGCTCCAATAGCGTATATTTAAGTTGTTGCAGTTAAAAAGCTCGTAGTTGAACCTTGGGTTGGGCAGAGCGGTCCGCCCCTGGTGTGCACCGG
TCTGCTCGTCCCTTCTACCGGCGATGCGCTCCTGGCCTTAACTGGCCGGGTCGTGCCTCCGGTGCTGTTACTTTGAAGAAATTAGAGTGCTCAAAGCAAGCCTACGCTCTGGATACATTAGCATGGGATAACATCATAGGATTTCGGTCCTATTCTGTTGGCCTTCGGGA
TCGGAGTAATGATTAACAGGGACAGTCGGGGGCATTCGTATTTCATAGTCAGAGGTGAAATTCTTGGATTTATGAAAGACGAACAACTGCGAAAGCATTTGCCAAGGATGTTTTCATTAATCAAGAACGAAAGTTGGGGGCTCGAAGACGATCAGATACCGTCCTAGTCT
CAACCATAAACGATGCCGACCAGGGATCGGCGGATGTTACTTATAGGACTCCGCCGGCACCTTATGAGAAATCAAAGTCTTTGGGTTCCGGGGGGAGTATGGTCGCAAGGCTGAAACTTAAAGGAATTGACGGAAGGGCACCACCAGGAGTGGAGCCTGCGGCTTAATTT
GACTCAACACGGGGAAACTTACCAGGTCCAGACATAGTAAGGATTGACAGACTGAGAGCTCTTTCTTGATTCTATGGGTGGTGGTGCATGGCCGTTCTTAGTTGGTGGAGTGATTTGTCTGGTTAATTCCGTTAACGAACGAGACCTCAGCCTGCTAACTAGCTATGCGG
AGGTGACCCTCCGCGGCCAGCTTCTTAGAGGGACTATGGCCGCTTAGGCCAAGGAAGTTTGAGGCAATAACAGGTCTGTGATGCCCTTAGATGTTCTGGGCCGCACGCGCGCTACACTGATGTATTCAACGAGTTTATAGCCTTGGCCGACAGGCCCGGGTAATCTTTGA
AATTTCATCGTGATGGGGATAGATCATTGCAATTGTTGGTCTTCAACGAGGAATTCCTAGTAAGCGCGAGTCATCAGCTCGCGTTGACTACGTCCCTGCCCTTTGTACACACCGCCCGTCGCTCCTACCGATTGAATGGTCCGGTGAAGTGTTCGGATCGCGGCGACGTG
GGCGGTTCGCTGCCGGCGACGTCGCGAGAAGTCCACTGAACCTTATCATTTAGAGGAAGGAGAAGTCGTAACAAGGTTTCCGTAGGTGAACCTGCGGAAGGATCATTG

Next is 227 bp spacer between 18S and 28S:

TCGAAACCTGCACAGCAGAACGACCCGCGAATTGGTTACAACCGACGGGGGGCGGGGGGCGTTCGTCGCCCCCTCGCCCCCTCCTGCGGGCGGGGACCTCGTGTCTCCTGCCCGCAAACCGAACCCCGGCGCGGAACGCGCCAAGGAAATCTAACCAAGAGAGCCATGCC
GGAGGCCCCGGACACGGTGCGCCCCCGGCGTCGGCGTCTTATGAATTATTCAAAACG

Next is 4,129 bp 28S (per RNAmmer); tail ≈300 bp is repetitive and similar to 2,943 bp spacer head:

ACTCTCGGCAACGGATATCTAGGCTCTCGCATCGATGAAGAACGTAGCGAAATGCGATACTTGGTGTGAATTGCAGAATCCCGCGAATCATCGAGTTTTTGAACGCAAGTTGCGCCCGAAGCCATTCGGCCGAGGGCACGTCTGCCTGGGTGTCACGCATCGTTGCCCCC
CTCAAACTCCGGTTCGGGCGGGGCGGAAGTTGGCCTCCCGTGCGTGCCTGCACGCGCGGTTAGCCCAAAAGCGAGTCCTCGGCGACGAGCGCCACGACAATCGGTGGTTTTTTTACCCTCGTTCCTCGTCGTGCGTGCCCCGTCGCCCGAACGCGCTCCTCCGACCCTCA
CGCGTCGCCTCGGTGGCGCTCCCAACGCGACCCCAGGTCAGGCGGGACTACCCGCTGAGTTTAAGCATATCAATAAGCGGAGGAAAAGAAACTTACAAGGATTCCCCTAGTAACGGCGAGCGAACCGGGAACAGCCCAGCTTGAGAATCGGGCGCCCTCACGGGCGTCTC
CGAATTGTAGTCTGGAGAAGCGTCCTCAGCGGCGGACCGGGCCCAAGTCCCCTGGAAGGGGGCGCCGGAGAGGGTGAGAGCCCCGTCGTGCCCGGACCCTGTCGCACCACGAGGCGCTGTCGGCGAGTCGGGTTGTTTGGGAATGCAGCCCCAATCGGGCGGTAAATTCC
GTCCAAGGCTAAATACGGGCGAGAGACCGATAGCAAACAAGTACCGCGAGGGAAAGATGAAAAGGACTTTGAAAAGAGAGTCAAAGAGTGCTTGAAATTGTCGGGAGGGAAGCGGATGGGGGCCGGCGATGCGCCCCGGTCGGATGTGGAACGGCGACAGCCGGTCCGCC
GATCGACTCGGGGCGTGGACCGATGCGGATTGCGGCGGCGGCCCAAGCCCGGGCTGTAGTTATGCCCGTGGAGACGTCGTTGCCGCGATCGTGGTTGGCAGCGCGCGCCTCACGGCGTGCCTCGGCATCTGCGCGCTCCTGGCATCGGCCTGCGGGCTCCCCATTCGGCC
CGTCTTGAAACACGGACCAAGGAGTCTGACATGTGTGCGAGTCAACGGGCCAGTAAACCCGTAAGGCGCAAGGAAGCTGATTGGCGGGATCCCCTTGAGGGTTGCACCGCCGACCGACCTTGATCTTCTGAGAAGGGTTCGAGTGAGAGCATACCTGTCGGGACCCGAAA
GATGGTGAACTATGCCTGAGCGGGGCGAAGCCAGAGGAAACTCTGGTGGAGGCCCGCAGCGATACTGACGTGCAAATCGTTCGTCTGACTTGGGTATAGGGGCGAAAGACTAATCGAACCGTCTAGTAGCTGGTTCCCTCCGAAGTTTCCCTCAGGATAGCTGGAGCCCA
CGTGCGAGTTCTATCGGGTAAAGCCAATGATTAGAGGCATCGGGGGCGCAACGCCCTCGACCTATTCTCAAACTTTAAATAGGTAGGACGGCGCGGCTGCTTCGTTGAGCCGCGCCAAGGAATCGAGAGCTCCAAGTGGGCCATTTTTGGTAAGCAGAACTGGCGATGCG
GGATGAACCGGAAGCCGGGTTACGGTGCCCAACTGCGCGCTAACCTAGAACCCACAAAGGGTGTTGGTCGATTAAGACAGCAGGACGGTGGTCATGGAAGTCGAAATCCGCTAAGGAGTGTGTAACAACTCACCTGCCGAATCAACTAGCCCCGAAAATGGATGGCGCTG
AAGCGCGCGACCTATACCCGGCCGTCGGGGCAAGTTCTAGGCCCCGATGAGTAGGAGGGCGCGGCGGTCGCTGCAAAACCTGGGGCGCGAGCCCGGGCGGAGCGGCCGTCGGTGCAGATCTTGGTGGTAGTAGCAAATATTCAAATGAGAACTTTGAAGGCCGAAGAGGG
GAAAGGTTCCATGTGAACGGCACTTGCACATGGGTTAGTCGATCCTAAGAGACGGGGGAAGCCCGTCTGATAGCGTGCTAAGCGCGAGCTTCGAAAGGGAATCGGGTTAAAATTCCTGAACCGGGACGTGGCGGCTGACGGCAACGTTAGGGAGTCCGGAGACGTCGGCG
GGGGCCTCGGGAAGAGTTATCTTTTCTGTTTAACAGCCTGCCCACCCTGGAAACGGCTCAGCCGGAGGTAGGGTCCAGCGGCTGGAAGAGCACCGCACGTCGCGTGGTGTCCGGTGCGCCCCCGGCGGCCCTTGAAAATCCGGAGGACCGAGTGCCTCCCACGCCCGGTC
GTACTCATAACCGCATCAGGTCTCCAAGGTGAACAGCCTCTGGTCGATGGAACAATGTAGGCAAGGGAAGTCGGCAAAATGGATCCGTAACCTCGGGAAAAGGATTGGCTCTGAGGGCTGGGCACGGGGGTCCCAGTCCCGAACCCGTCGGCTGTCGGCGGACTGCTCGA
GCTGCTCCCGCGGCGAGAGCGGGTCGCCGCGTGCCGGCCGGGGGACGGACTGGGAACGACCGCTTCGGCGGTCTTCCCCGGGCGTCGAACAGTCGACTCAGAACTGGTACGGACAAGGGGAATCCGACTGTTTAATTAAAACAAAGCATTGCGATGGTCCCTGCGGATGC
TCACGCAATGTGATTTCTGCCCAGTGCTCTGAATGTCAAAGTGAAGAAATTCAACCAAGCGCGGGTAAACGGCGGGAGTAACTATGACTCTCTTAAGGTAGCCAAATGCCTCGTCATCTAATTAGTGACGCGCATGAATGGATTAACGAGATTCCCACTGTCCCTGTCTA
CTATCCAGCGAAACCACAGCCAAGGGAACGGGCTTGGCAGAATCAGCGGGGAAAGAAGACCCTGTTGAGCTTGACTCTAGTCCGACTTTGTGAAATGACTTGAGAGGTGTAGGATAAGTGGGAGCCGAAAGGCGAAAGTGAAATACCACTACTTTTAACGTTATTTTACT
TATTCCGTGAATCGGAAGCGGGGCTCTGCCCCTCTTTTTGGACCCAAGGCTCGCCTCGGCGGGCCGATCCGGGCGGAAGACATTGTCAGGTGGGGAGTTTGGCTGGGGCGGCACATCTGTTAAAAGATAACGCAGGTGTCCTAAGATGAGCTCAACGAGAACAGAAATCT
CGTGTGGAACAAAAGGGTAAAAGCTCGTTTGATTCTGATTTCCAGTACGAATACGAACCGTGAAAGCGTGGCCTATCGATCCTTTAGACCTTCGGAATTTGAAGCTAGAGGTGTCAGAAAAGTTACCACAGGGATAACTGGCTTGTGGCAGCCAAGCGTTCATAGCGACG
TTGCTTTTTGATCCTTCGATGTCGGCTCTTCCTATCATTGTGAAGCAGAATTCACCAAGTGTTGGATTGTTCACCCACCAATAGGGAACGTGAGCTGGGTTTAGACCGTCGTGAGACAGGTTAGTTTTACCCTACTGATGACAGTGTCGCAATAGTAATTCAACCTAGTA
CGAGAGGAACCGTTGATTCGCACAATTGGTCATCGCGCTTGGTTGAAAAGCCAGTGGCGCGAAGCTACCGTGCGCTGGATTATGACTGAACGCCTCTAAGTCAGAATCCGGGCTAGAAGCGACGCGTGCGCCCGCCGCCCGATTGCCGACCTGCAGTAGGGGCCCTCGGG
CCCCCAGAGGCACGTGTCTTTGGCCAAGCCCCGCGGCGGACGAGCCGCGTGGGCCGCCATGAAGTATAATTCCCACCGAGCGGCGGGTAGAATCCTTTGCAGACGACTTAAATACGCGACGGGGTATTGTAAGTGGCAGAGTGGCCTTGCTGCCACGATCCACTGAGAT
TCAGCCCTGTGTCGCTTCGATTCGTCCCTCCCCCCTCTCCTCTCCCAATCCCCCCCTCAAAAAAAAAATCAACTCTTTGCCCCGTGCCCTCCGGAGGCTAGCCCCCCGAGTGCCTTCGCTGCGTGCCCCTTGCCCATGACAGGCTGCCTTGGCCTTGGCCTTCGCTGCTT
GCCTATGGGGGGTGCCTTGGCCTTGGCTGCGTGCCCTTGCCTTGGCAGCATGCCCATGGGGGGCTGCTGCGTGCCCTTGCCCTTGGCTGCATGCCCGTGGGGGGCTGCTGCTGCCTTGGCCTTGGCTGCATGCCTTGGCCTTGGCCTTGGCTGCATGCCTTGGCCTTGGC
CTTGGCTGCGTGCCTTGGCCTTGGCCTTGGCTGCATGCCTTGGCCTTGG

Final 18 bp is not included by RNAmmer as part of 28S, but is part of repetitive tail approximately closing circle:

CTGCATGCCATGGCCTTG

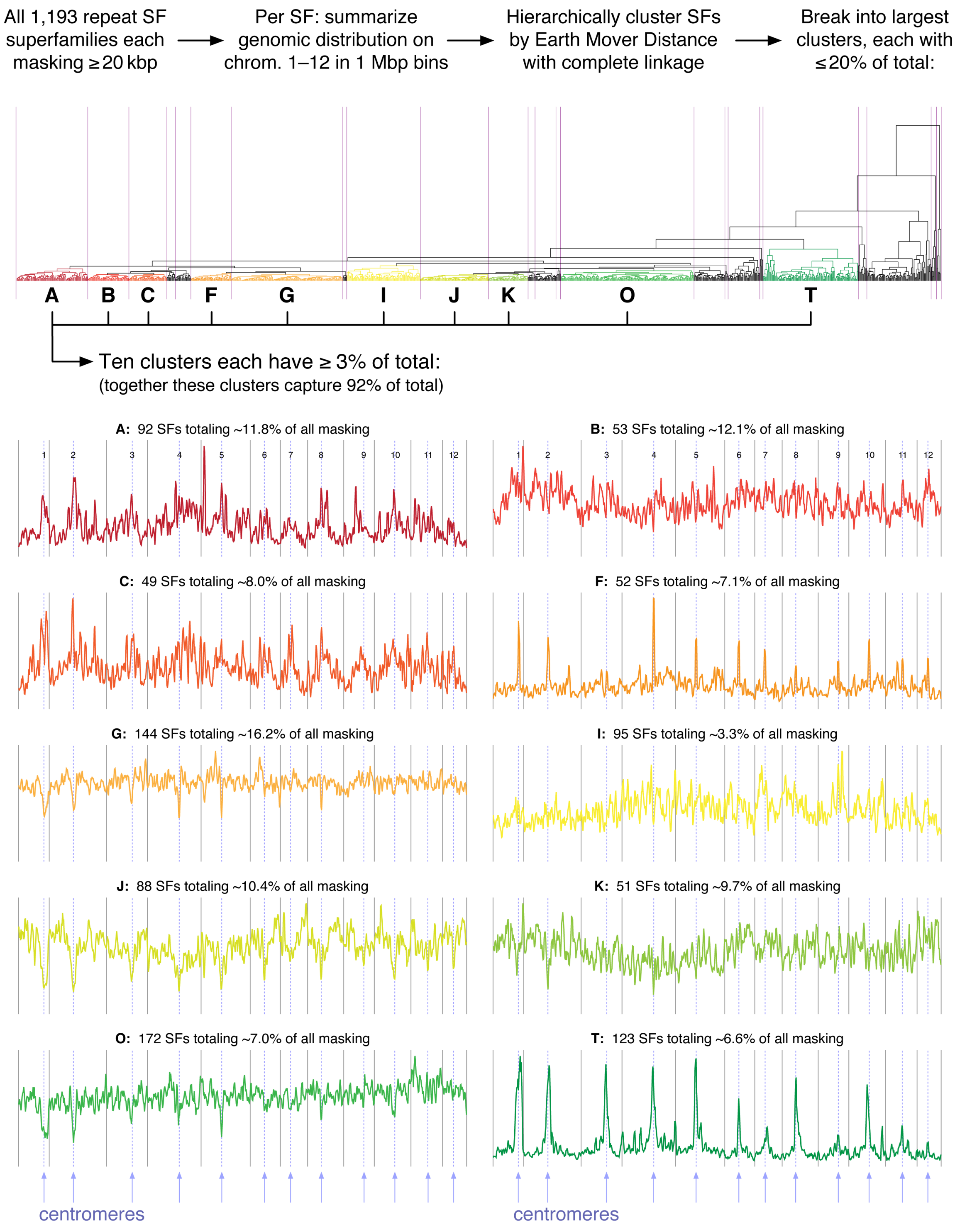

Supplementary Figure 13. Unsupervised clustering indicates that the dominant chromosome-scale distributional features of repeats in *Q. lobata* are correlated with distance to the centromeres.

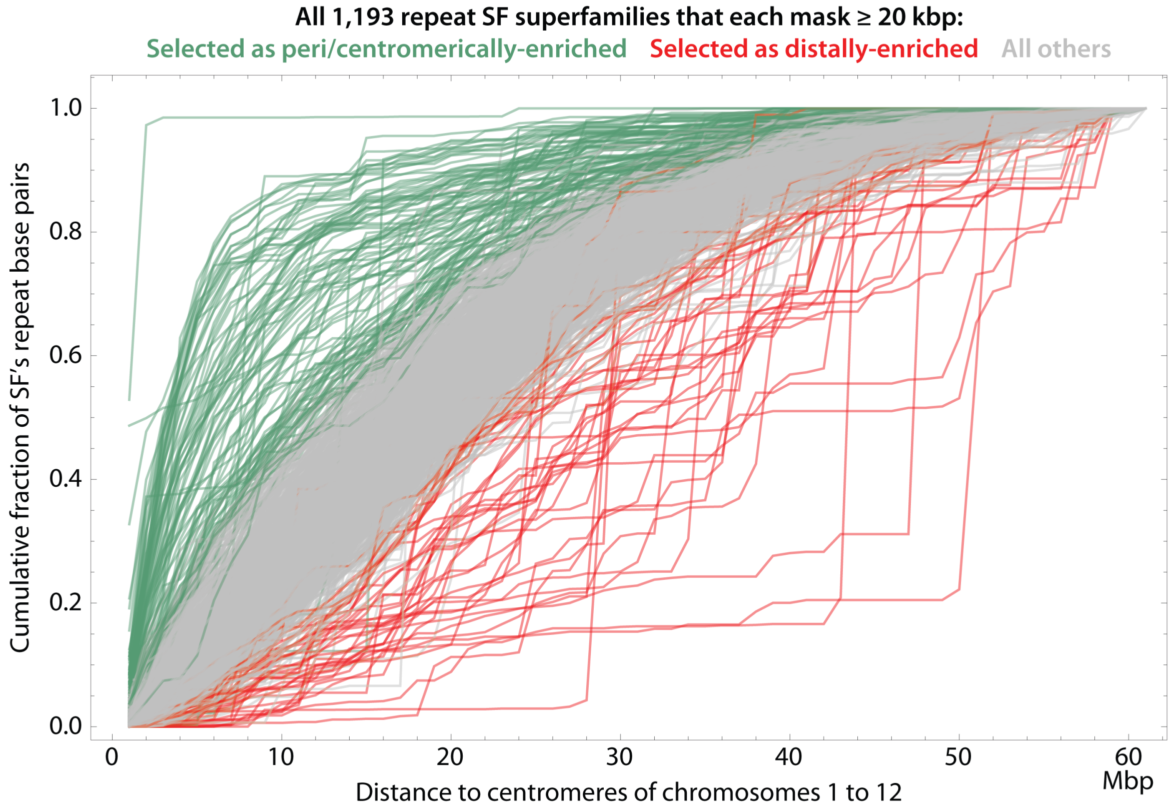

Supplementary Figure 14. Average centromeric distance summarizes repeat per-SF distribution of distances well. For each repeat superfamily (SF) of at least minimal size (≥ 20 kbp masked), the cumulative distribution function (CDF) of the distance of the repeat's base pairs to the centromeres is shown. Coloring by green, red, and gray is as in Figure 3C and shows that the average centromeric distance per SF summarizes the distributions well, with outliers at the distribution level essentially coinciding with outliers at the average level. Curve plotting order is randomized across all 1,193 SFs.

Supplementary Note 7. Gene model statistics and possible R-genes in *Q. lobata*, *Q. robur*, and *Q. suber*

Basic statistics for the three *Quercus* protein-coding gene (PCG) sets are given in **Supplementary Table 3**. (For *Q. suber*, 12% of PCG loci have multiple transcript models; a single longest isoform was chosen per locus.) *Q. robur* has many fewer models (26k), while *Q. suber* more (49k, but a more comparable 36k with at least one intron). Further, at least 11k *Q. suber* models are incomplete, and 1k actually interpolate CDS beyond the assembly. By models-per-Mbp-of-non-gap-assembly, *Q. robur* is low (33), with *Q. lobata* and *Q. suber* quite similar (47 and 53). *Q. robur* calls a total of only 30 Mbp of CDS (gene spans cover just 10% of its assembly) vs. 50 and 67 Mbp (25% and 20%) for *Q. lobata* and *Q. suber*. While every *Q. lobata* model has both UTR5 and UTR3 annotated (affecting many size-related quantities of **Supplementary Table 3**), only about half of *Q. robur* and *Q. suber* models have UTRs, with *Q. suber* tending short and *Q. robur* shorter when they do have UTRs. CDS lengths are fairly similar (and have similar depletion of repetitive sequence), although *Q. suber* and (to a lesser extent) *Q. robur* tend to have fewer exons, perhaps due to their higher assembly incontiguity. While no *Q. lobata* models have CDS that contain assembly gaps, 0.2% and 0.8% do so in *Q. suber* and *Q. robur*; for exons, this rises to 0.2% vs. 0.3% and 1.1%, and for gene spans to 0.6% vs. 6.5% and 6.9% (suggesting the other assemblies unsurprisingly have gaps concentrated in introns). Based on a HMMer search for GyDB domains, *Q. robur* is the most conservative, where only 0.1% of models have at least one domain strongly indicative of a transposon (rising to 0.5% for domains correlated with transposons); *Q. lobata* is somewhat higher (0.7% and 1.4%), but *Q. suber* is much higher (3.0% and 4.6%).

Supplementary Table 3. Statistics of protein-coding gene (PCG) models for *Q. lobata*, *Q. robur*, and *Q. suber*.

Statistic	*Q. lobata*	*Q. robur*	*Q. suber*
**# PCG (Protein-Coding Gene models)[*]**	**39,373**	**25,808**	**49,388**
# PCG^5 (PCGs with non-empty UTR5)	39,373	13,625	24,282
# PCG^3 (PCGs with non-empty UTR3)	39,373	14,132	24,348
# PCG^i (PCGs with at least one intron)	34,859	20,356	35,822
# PCGs with an ostensibly complete[†] CDS	39,373	25,808	38,499
# PCGs with CDS not entirely[‡] from the assembly	0	0	1,151
# PCGs with ≥1 H [H or M][§] transposon domain	288 [537]	21 [130]	1,462 [2,275]
Knt between adj. PCG spans: average [median]	15.6 [8.5]	27.7 [14.8]	13.1 [5.3]
Span kilobases per PCG: average [median]	5.4 [4.2]	3.1 [2.3]	3.9 [2.3]
Exonic kilobases per PCG: average [median]	2.3 [2.0]	1.3 [1.1]	1.6 [1.4]
CDS kilobases per PCG: average [median]	1.3 [1.0]	1.2 [0.9]	1.4 [1.1]
UTR5 kilobases per PCG^5: average [median]	0.4 [0.3]	0.2 [0.1]	0.2 [0.2]
UTR3 kilobases per PCG^3: average [median]	0.7 [0.5]	0.1 [0.1]	0.3 [0.2]
Intronic kilobases per PCG^i: average [median]	3.5 [2.4]	2.2 [1.5]	3.1 [1.5]
# exon intervals per PCG: average [median]	5.5 [4.0]	4.4 [3.0]	4.1 [3.0]
# CDS intervals per PCG: average [median]	4.8 [3.0]	4.3 [3.0]	3.9 [2.0]
# UTR5 intervals per PCG^5: average [median]	1.3 [1.0]	1.0 [1.0]	1.2 [1.0]
# UTR3 intervals per PCG^3: average [median]	1.4 [1.0]	1.0 [1.0]	1.1 [1.0]
Mbp in union of all PCG… exons [CDS]	92.2 [49.9]	34.8 [30.3]	78.6 [67.0]
Mbp in union of all PCG… UTR5 [UTR3]	15.7 [26.6]	2.4 [2.1]	4.8 [6.8]
Mbp in union of all PCG… introns [spans]	121.2 [213.5]	45.1 [79.9]	111.1 [189.3]
% of asm. in union PCG… exons [CDS]	10.9% [5.9%]	4.3% [3.7%]	8.2% [7.0%]
% of asm. in union PCG… UTR5 [UTR3]	1.9% [3.1%]	0.3% [0.3%]	0.5% [0.7%]
% of asm. in union PCG… introns [spans]	14.3% [25.2%]	5.5% [9.8%]	11.7% [19.9%]
% of non-gap assembly that is repetitive[ǀ]	54.4%	54.3%	51.6%
% repetitive[ǀ] in union PCG… exons [CDS]	16% [14%]	12% [13%]	13% [14%]
% repetitive[ǀ] in union PCG… UTR5 [UTR3]	18% [18%]	17% [6%]	12% [8%]
% repetitive[ǀ] in union PCG… introns [spans]	26% [22%]	18% [16%]	30% [23%]
% PCG w/ ≥1 asm. gap in… exons [CDS]	0.2% [0.0%]	1.1% [0.8%]	0.3% [0.2%]
% PCG w/ ≥1 asm. gap in… UTR5 [UTR3]	0.1% [0.1%]	0.2% [0.1%]	0.0% [0.0%]
% PCG w/ ≥1 asm. gap in… introns [span]	0.4% [0.6%]	6.0% [6.9%]	6.3% [6.5%]

[*] Explicit non-nuclear assembly components are removed, and only a single longest PCG isoform is kept per PCG-containing gene locus.

[†] *Ostensibly complete* means the CDS, as derived exclusively from the assembly, starts on a codon boundary with a start codon, ends on a codon boundary with a stop codon, and has no internal stop codons.

[‡] The NCBI genebuild pipeline (*Q. suber*) can make models that apply edits (e.g., additions of 100's of basepairs) to the reference assembly; generally all table data is based on the pure-assembly portions.

[§] GyDB 2019-03-21 HMMer 3.2.1 full-sequence hits of E-value ≤ 10^{-5}; 'H' (high) is ≥1 of GAG/GAGCOAT/RT/INT/galadriel/TAV, 'M' (medium) is ≥1 of AP/RNaseH/CHR/DUT/MOV/ENV.

[ǀ] All (non-gap) basepairs that are masked by a run of RepeatMasker-after-RepeatModeler (as in Figure 3A) for each assembly.

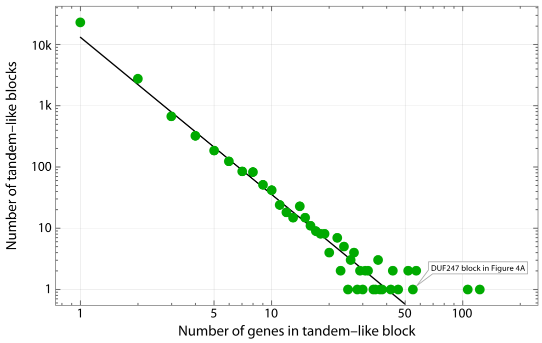

Supplementary Figure 15. Log–log size of tandem-like duplicated gene blocks versus frequency. Black line is fitted power decay rate (number ≈ 13,139 / size$^{2.567}$).

Methods for identification of possible R-genes

Gururani, et al. [47] provide an overview of the numerous types of plant disease resistance genes ("R-genes"), which allow plants to detect pathogen attacks from bacteria, viruses, nematodes, oomycetes, fungi, and insects, and facilitate counterattacks against them. In reviewing studies of R-genes, they propose eight classes of R-gene domain/motif architectures:

I.	NBS–LRR–TIR	Cytoplasmic proteins with a NBS (nucleotide-binding site) domain and LRR (leucine rich repeat), plus a TIR (Toll-Interleukin receptor) domain
II.	NBS–LRR–CC	NBS, LRR, and CC (coiled coil) at the N-terminus
III.	LRR–TrD	Extra cytoplasmic LRR (eLRR) attached to a transmembrane domain (TrD)
IV.	LRR–TrD–KIN	eLRR, TrD, and an intracellular KIN (serine-threonine kinase) domain
V.	TrD–CC	TrD fused to a CC
VI.	LRR–TrD–PEST–ECS	eLRRs and TrD, plus a PEST degradation domain and ECS short proteins motif
VII.	TIR–NBS–LRR–NLS–WRKY	*Arabidopsis* RRS1-R gene conferring resistance to *Ralstonia solanacearum*
VIII.	KIN / KIN–KIN / HM1	Enzymatic R-genes without NBS, LRR, or TIR.

A detailed study of R-genes would be its own project; we wish to computationally operationalize in a feasible way with limited effort that still has good sensitivity and selectivity. To this end, we decided thusly: (1) We ignore order and multiple copy number of domains/motifs, and focus on just the subset of distinct features present in a given gene. (2) We take patterns as not exact but as presence minimums; instances of additional domains/motifs are not disqualifying. (3) Class IV with KIN dropped is Class III, Class VI with PEST and ECS dropped is Class III, and Class VII with NLS and WRKY dropped is Class I, and so we consider these special cases and subsume them into Classes III and I (given that NBS, LRR, and TIR are fairly indicative). (4) Class VIII is difficult and poorly characterized (e.g., one cannot just accept all protein kinases). (5) We equate the following:

NBS with *N* := instances of Pfam NB-ARC (there are no NB-LRR in any of the three oak proteomes)
LRR with *L* := instances of Pfam LRR_1, LRR_2, LRR_3, LRR_4, LRR_5, LRR_6, LRR_8, LRR_9, and LRRNT_2
(there are no LRR19-TM, LRR_adjacent, LRRC37, LRRC37AB_C, LRRCT, LRRFIP, LRRNT, LRV, LRV_FeS, or TTSSLRR)
TIR with *T* := instances of Pfam TIR and TIR_2 (there are no TIR-like)
KIN with *P* := instances of Pfam Pkinase, Pkinase_C, and Pkinase_Tyr
CC with *C* := coiled coil regions as identified by Coils 2.2.1
TrD with *M* := transmembrane regions as identified by TMHMM 2.0c.

For a given gene, we summarize its status relative to *N*, *L*, *T*, *P*, *C*, and *M* with the six-character string `NLTPCM` where if the trigger as defined above for a letter is not met, then the letter is replaced with an underscore (_). Thus, simplified Gururani classes correspond to Class I = `NLT***`, Class II = `NL**C*`, Class III = `*L***M`, and Class V = `****CM`, where character asterisk (*) is interpreted as a wildcard. (Class IV = `*L*P*M`, Class VI = `*L***M` +PEST +ECS, Class VII = `NLT***` +NLS +WRKY, and Class VIII = `***P**` [or HM1] with difficult additional constraints.)

However, based on Panther/InterProScan-derived *Q. lobata* gene names (and literature searches on some genes), adopting these classes directly did not seem to empirically perform well in terms of low false negatives and low

false positives. Hence, decisions continued, leading to **Supplementary Table 4** below (and see main text that refers to the table): (6) Gururani classes are not to be used precisely, but only as suggestive of what domains and motifs (*N*, *T*, *L*, *P*, *C*, and *M*) are to be incorporated into the R-gene identification process. (7) *N* and *T* boost R-gene likelihood, but *L* to a lesser extent (preferring it to occur with *N* and/or *T*, and perhaps *P* or *M*). *P* is generally too weak on its own (being mostly just general protein kinases), and *C* and *M* are far too weak on their own (being mostly just general coiled coil or transmembrane proteins). (8) Each individual six-character pattern needs empirical investigation.

Supplementary Table 4. **R-gene domain/motif analysis partitioning all *Q. lobata*, *Q. robur*, and *Q. suber* PCGs.** Patterns are grouped by descending general strength of R-gene-associatedness per examination of Panther-derived *Q. lobata* gene names (and literature searches); sorting within groups by descending total count across the three proteomes. Main text adopts green shading as R-genes and blue shading as possible additionals.

Fraction of *Q. lobata* genes appearing to be R-genes	Domain/motif presence	and applicable Gururani classes	*Q. lobata* 39,373 PCGs	*Q. robur* 25,808 PCGs	*Q. suber* 49,388 PCGs
**Strongly high:** few unnamed genes, and named genes all or almost all suggest R-gene	**NT____**		180	128	157
	**NTL___**	I (or VII)	68	55	60
	**N_L_C_**	II	76	43	42
	**N_L___**		35	56	48
	**NT__C_**		19	9	32
	**N___CM**	V + *N*	13	13	7
	**NTL_C_**	I (or VII) + *C*, II + *T*	6	11	5
	**NT___M**		10	4	5
	**NTL__M**	I (or VII) + *M*, III (or VI) + *N T*	6	3	2
	**N_L_CM**	II + *M*, III (or VI) + *N C*, V + *N L*	5	1	3
	**NT__CM**	V + *N T*	4	0	1
	**_T__CM**	V + *T*	1	0	1
	**_TL__M**	III (or VI) + *T*	1	0	0
	Subtotal		*424*	*323*	*363*
**Highly enriched:** same as strongly high, except substantial or high fraction of genes are unnamed	**__L__M**	III (or VI)	302	295	330
	**__LPCM**	III (or VI) + *P C*, IV + *C*, V + *L P*, poss. VIII	14	9	22
	**N____M**		11	5	8
	Subtotal		*327*	*309*	*360*
**Enriched potential:** may have high fraction of unnamed genes, but at least about half of named genes have names that are suggestive of an R-gene	**___P_M**	poss. VIII (+ *M*)	754	466	663
	**__LP_M**	III (or VI) + *P*, IV, poss. VIII (+ *L M*)	382	234	342
	**N_____**		266	314	347
	**N___C_**		344	223	308
	**__L___**		241	228	356
	**_T____**		102	139	101
	**___PCM**	V + *P*, poss. VIII (+ *C M*)	65	28	39
	**__L_C_**		22	13	26
	Subtotal		*2,176*	*1,645*	*2,182*
**Few genes:** often high fraction unnamed, but lean toward potential R-genes due to domains involved (*N* or *T* or *L*+*M* or *L*+*C* or *L*)	**__LP__**	poss. VIII (+ *L*)	34	18	23
	**_T___M**		15	10	6
	**__L_CM**	III (or VI) + *C*, V + *L*	4	7	13
	**_T__C_**		3	8	4
	**__LPC_**	poss. VIII (+ *L C*)	2	1	4
	**N_L__M**	III (or VI) + *N*	0	4	1
	**N__P__**	poss. VIII (+ *N*)	2	1	0
	**N__P_M**	poss. VIII (+ *N M*)	0	1	0
	**_TL___**		0	0	1
	Subtotal		*60*	*50*	*52*
**Likely low:** half unnamed, rest likely generic kinases	**___PC_**	poss. VIII (+ *C*)	83	43	113
	Subtotal		*83*	*43*	*113*
**Low fraction:** ≈5% to 10% of named genes have names that are R-gene suggestive	**___P__**	poss. VIII	729	657	873
	**____CM**	V	725	405	925
	Subtotal		*1,454*	*1,062*	*1,798*
**Very low fraction**	**______**		23,699	15,017	29,966
	**_____M**		7,007	5,017	8,251
	**____C_**		4,143	2,342	6,303
	Subtotal		*34,849*	*22,376*	*44,520*

Supplementary Note 8. Methylomes and analysis of methylation patterns

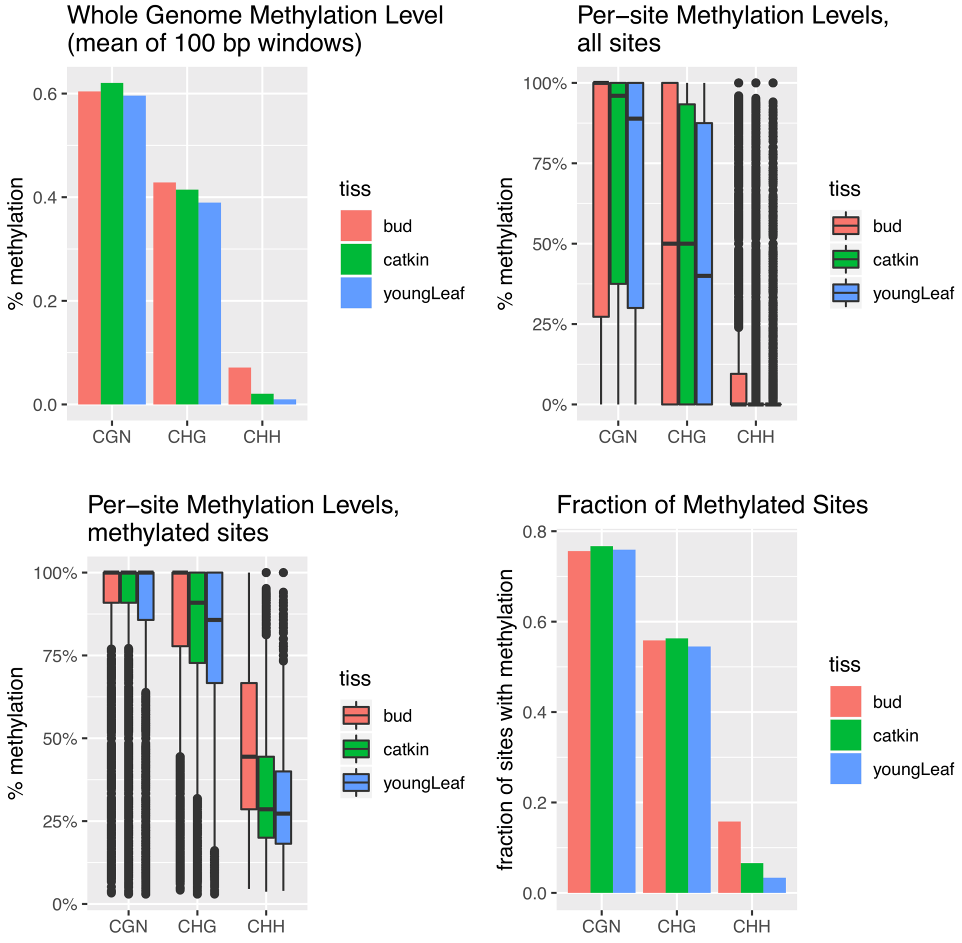

Supplementary Figure 16. Genome methylation levels for three tissues and three methylation contexts. Whole genome average methylation was calculated by averaging the methylation levels for 100 bp windows across chr. 1–12. Box plots (ggplot2 geom_boxplot defaults) show first / third quartiles and medians, with whiskers extending to 1.5 times interquartile ranges and points beyond plotted individually. Per-site methylation levels are for sites with a minimum strand-specific coverage of three reads, and is shown for all such sites and for sites considered methylated (by MethylDackel's binomial test for above background / non-conversion). Also shown are the fraction of sites that are considered methylated (minimum coverage of three reads). Methylation levels are consistent with a total absence (i.e., at bisulfite non-conversion estimated as ≈0.5%) at the majority of CHH sites (84%–97%), a large portion of C̲HG sites (43%–45%), and some C̲G sites (24%–25%), with methylation averages for the remaining sites much higher than the overall averages (mC̲HH 27%–45%, mC̲HG 86%–100%, mC̲G 100%).

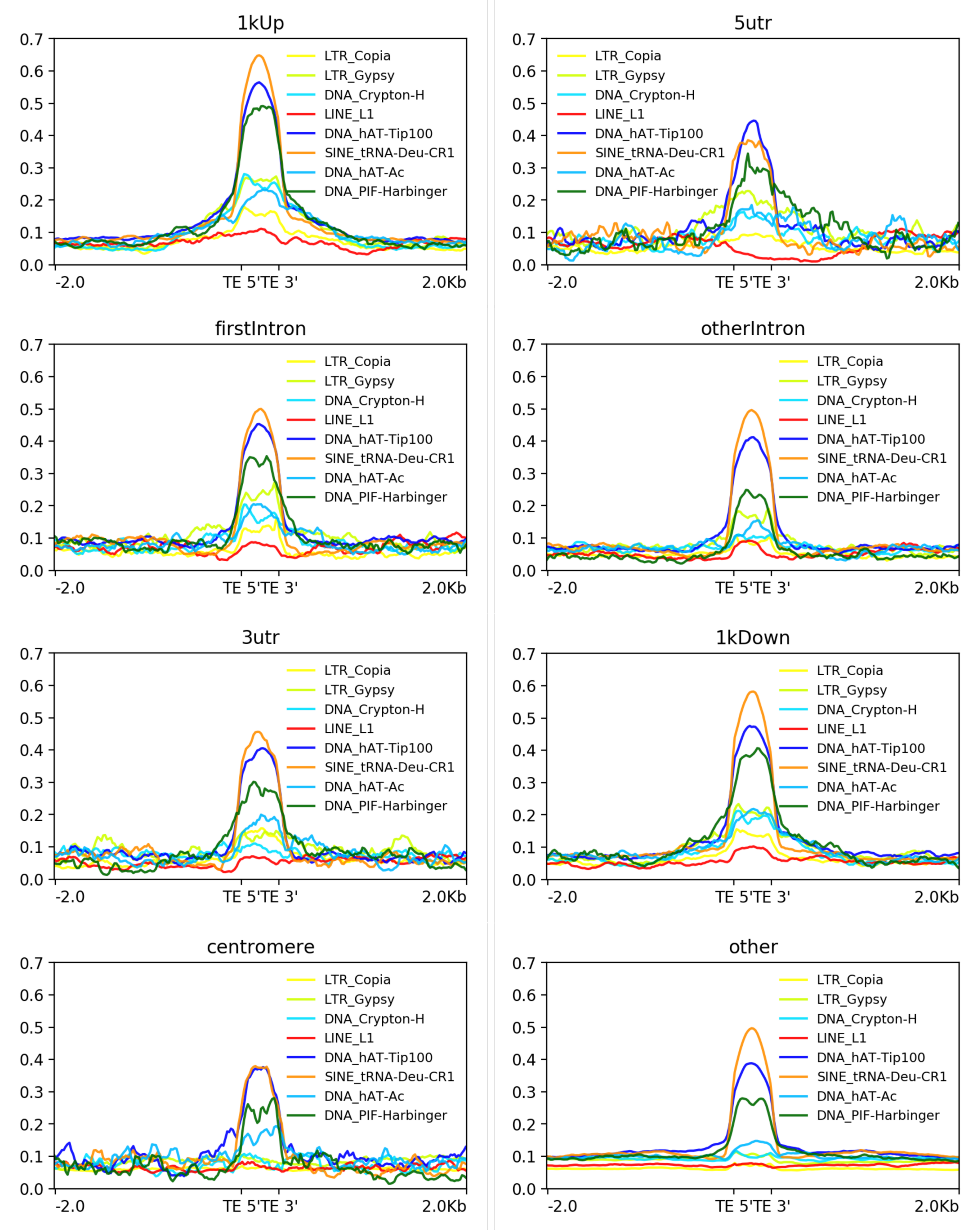

Supplementary Figure 17. CHH methylation levels in bud tissue across repeats. The “SINE_tRNA-Deu-CR1”, “DNA_hAT-Tip100”, and “DNA_PIF-Harbinger” show consistently high levels of mCHH across all regions. CDS regions are not shown due to small numbers of instances for some superfamilies. The “DNA_CMC-ENspm” and “DNA_MuLE-MuDR” were removed due to too few instance bases in several regions.

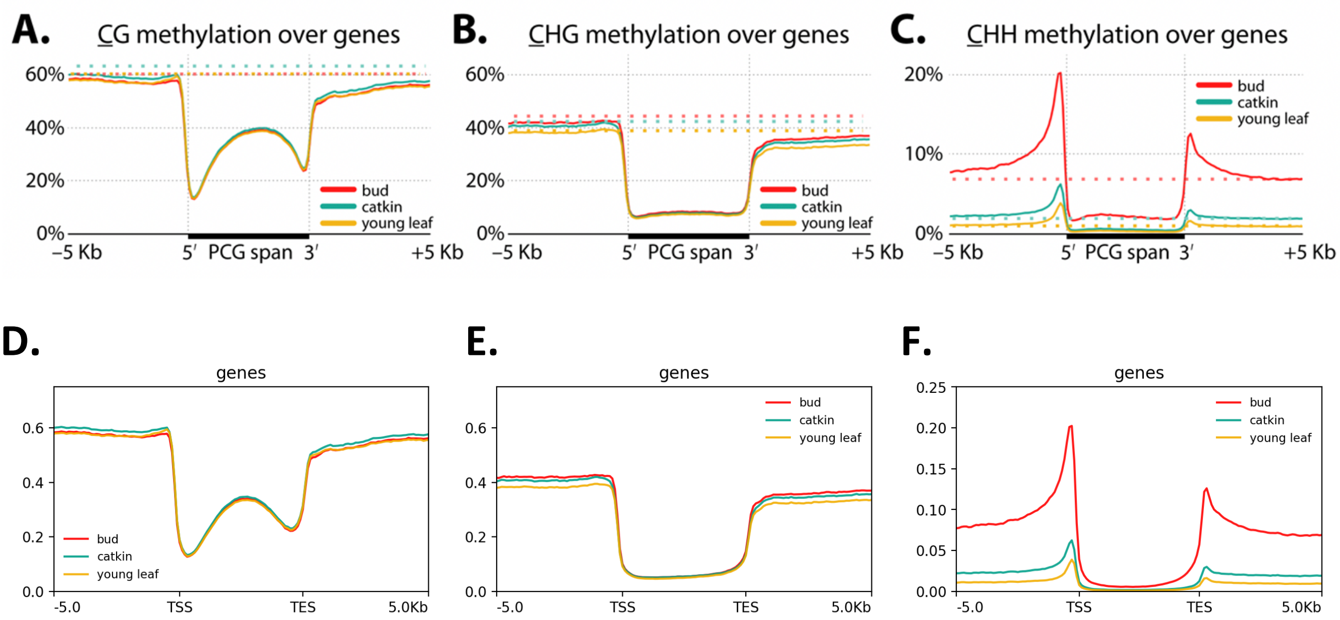

Supplementary Figure 18. Gene methylation metaplots, with and without introns.
(A)–(C) are identical to Figure 5A–C, and **(D)–(F)** are the same but with introns removed. Shown are average methylation levels (100 bp windows) with respect to PCGs (normalized to 5 kbp long) for the three sampled tissues (bud, catkin, and young leaf) by methylation context: **(A)/(D)** CG, **(B)/(E)** CHG, and **(C)/(F)** CHH. Dotted lines show genome-wide backgrounds, and TSS/TES = Transcription Start/End Site.

Supplementary Figure 19 (page 1 of 3). **Subcontext methylation for *Q. lobata* chromosomes 1 to 12 in 1 Mbp windows**. For each chromosome, **top** is number of protein coding genes, **middle** is mean mCHG by 3 nt subcontext, and **bottom** is mean mCHH by 3 nt subcontext.**.**

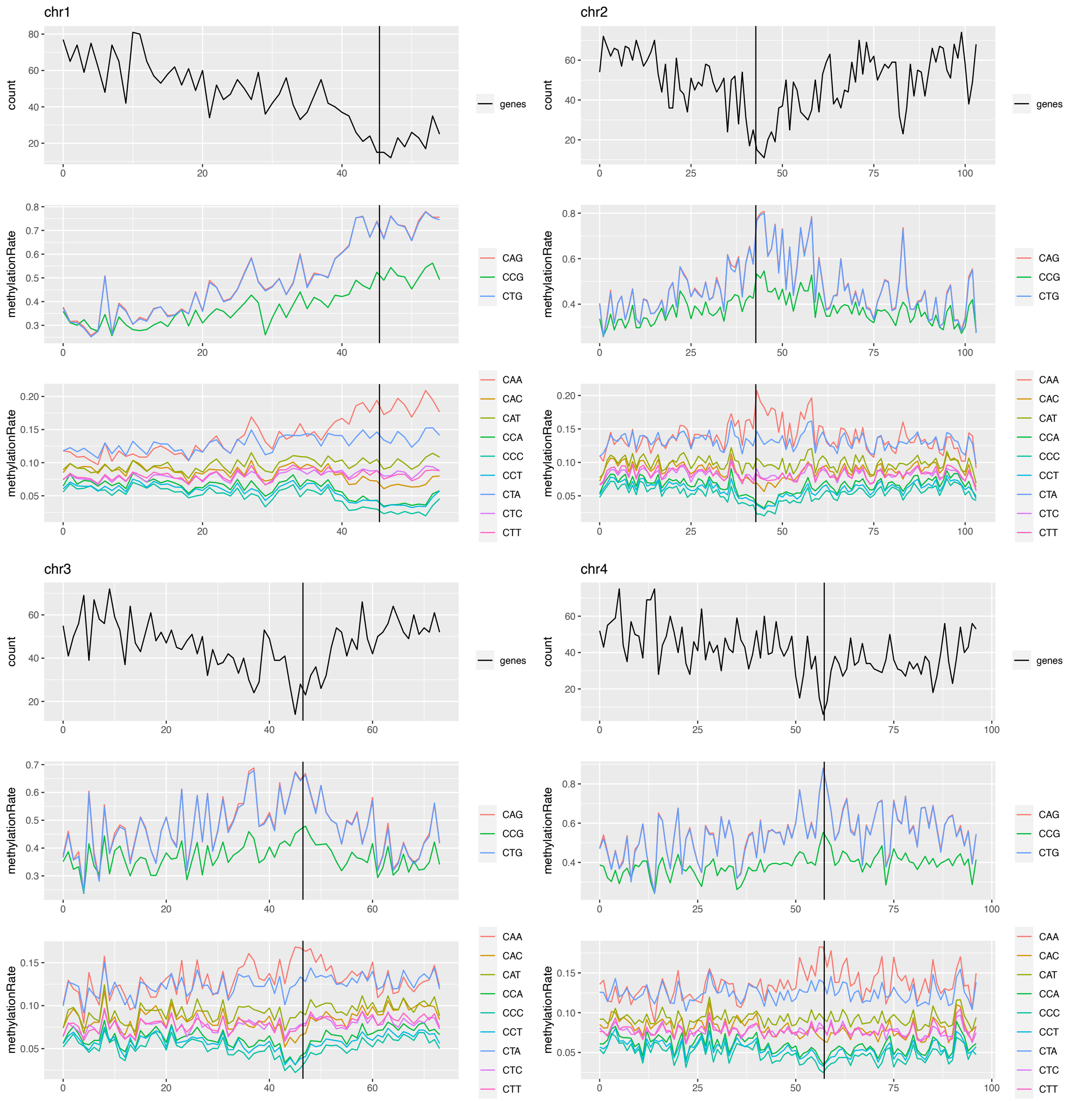

(continued Supplementary Figure 19, page 2 of 3**)**

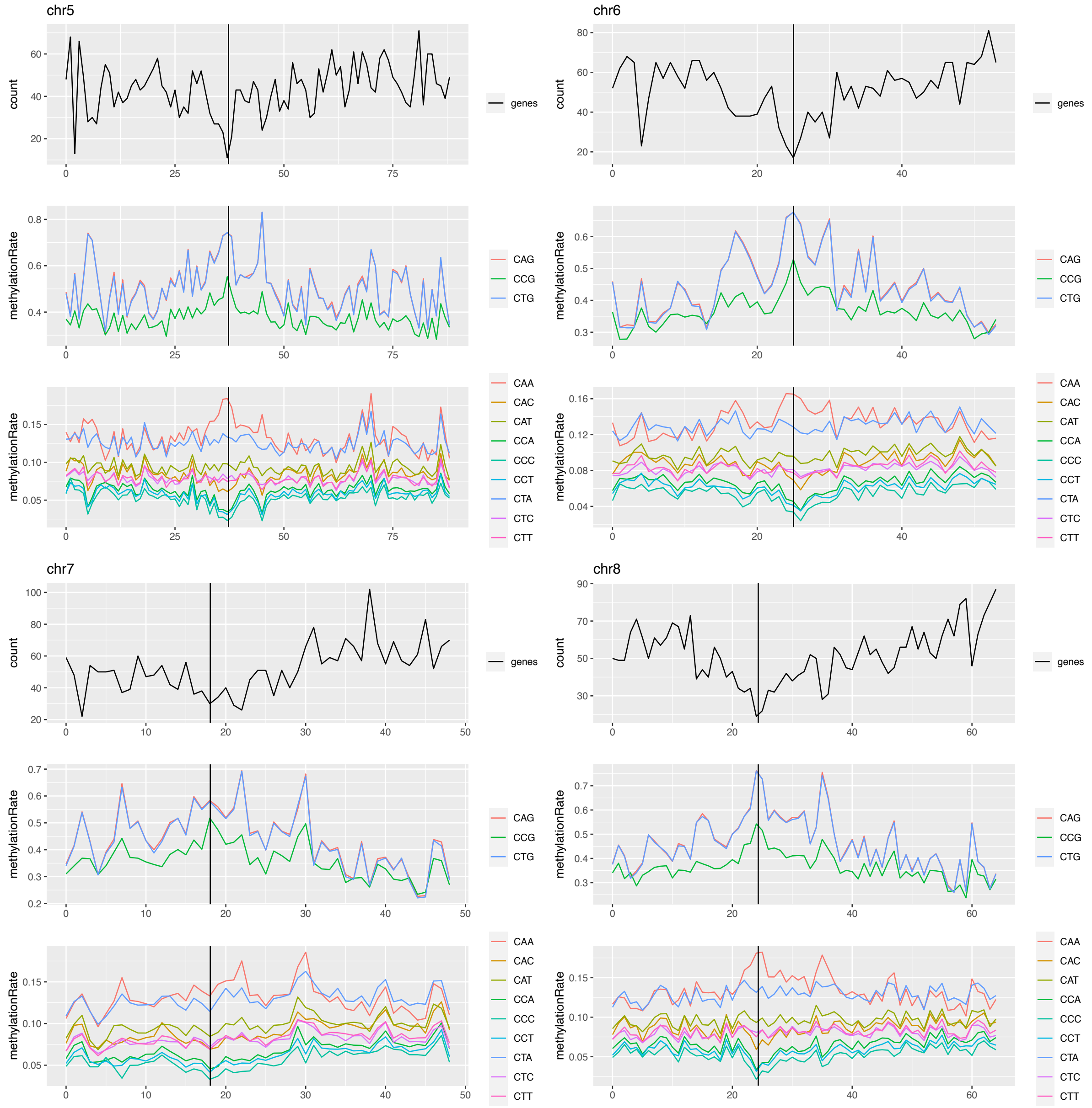

(continued Supplementary Figure 19, page 3 of 3**)**

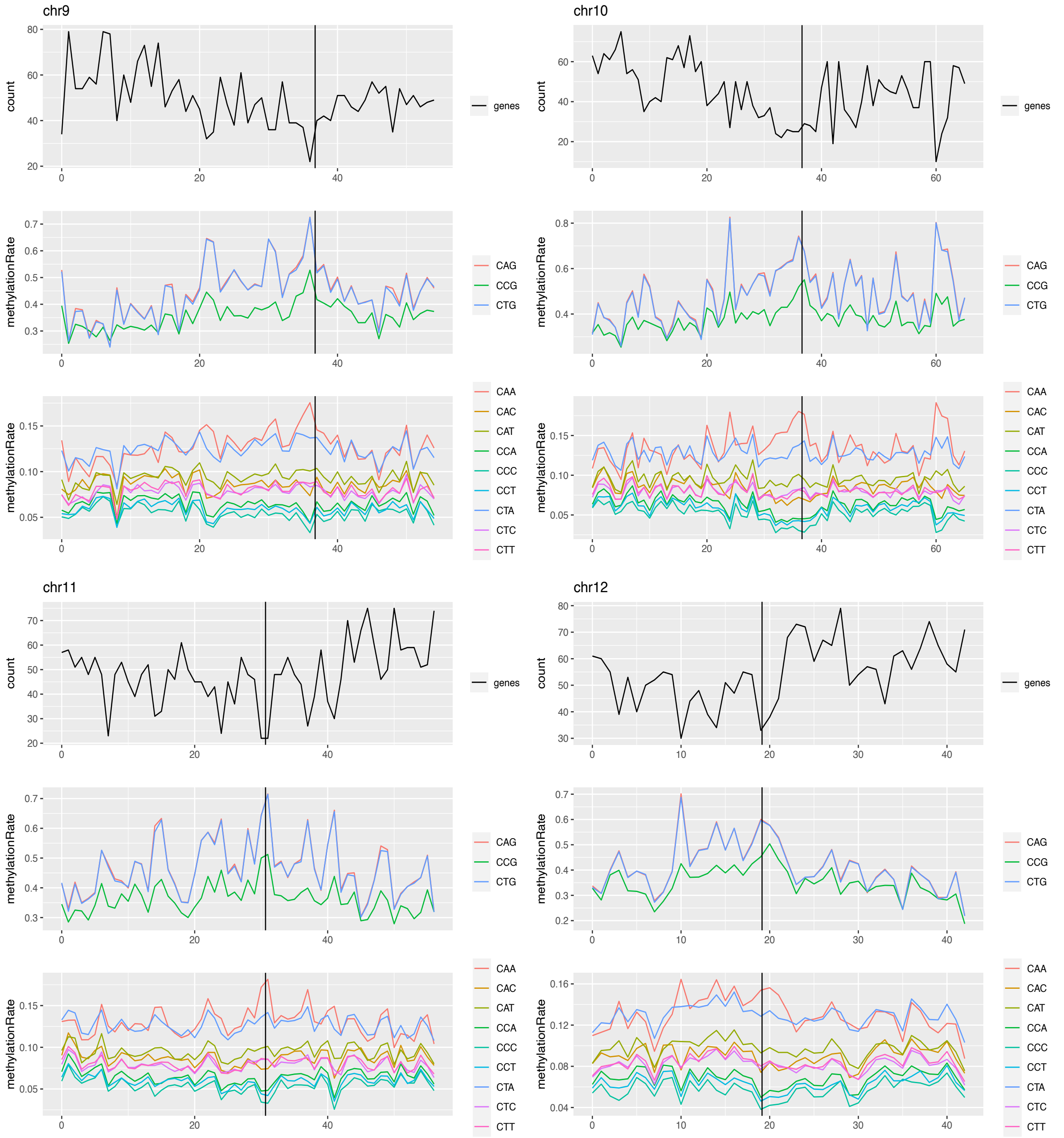

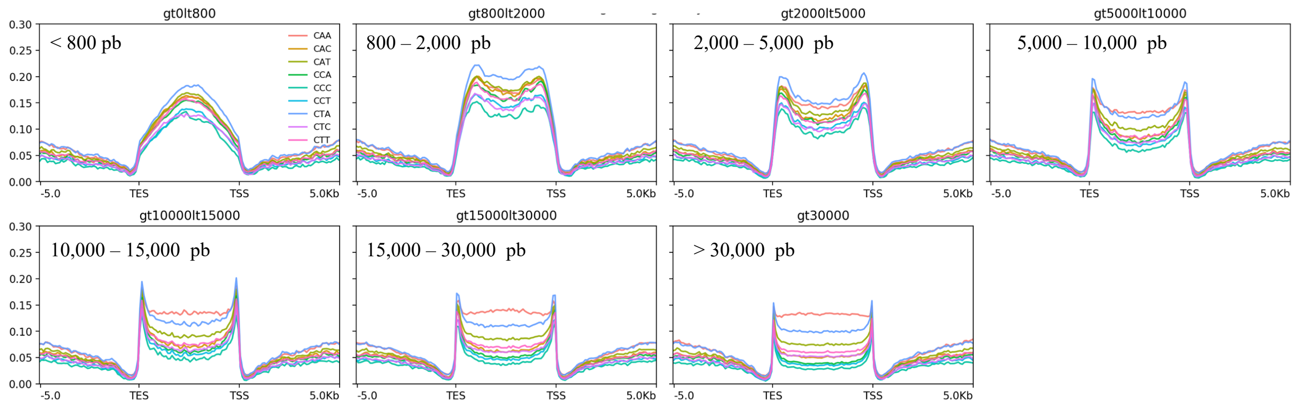

Supplementary Figure 20. Intergenic subcontext mCHH by size of region. Average bud tissue mCHH by 3 nt subcontext for intergenic regions, from a protein-coding gene's transcription end site (TES) to the next PCG's transcription start site (TSS), normalized to 5 kbp long and separated into six intergenic size ranges (one range per panel).

Supplementary Figure 21. Genic region methylation of oak in comparison with 34 angiosperms. Plots show mCG (blue), mCHG (green), and mCHH (maroon) upstream, across, and downstream averaged over genes, and are reprinted from Figure S18 from Niederhuth, Bewick [48], except with oak (*Q. lobata* young leaf and bud) added for comparison.

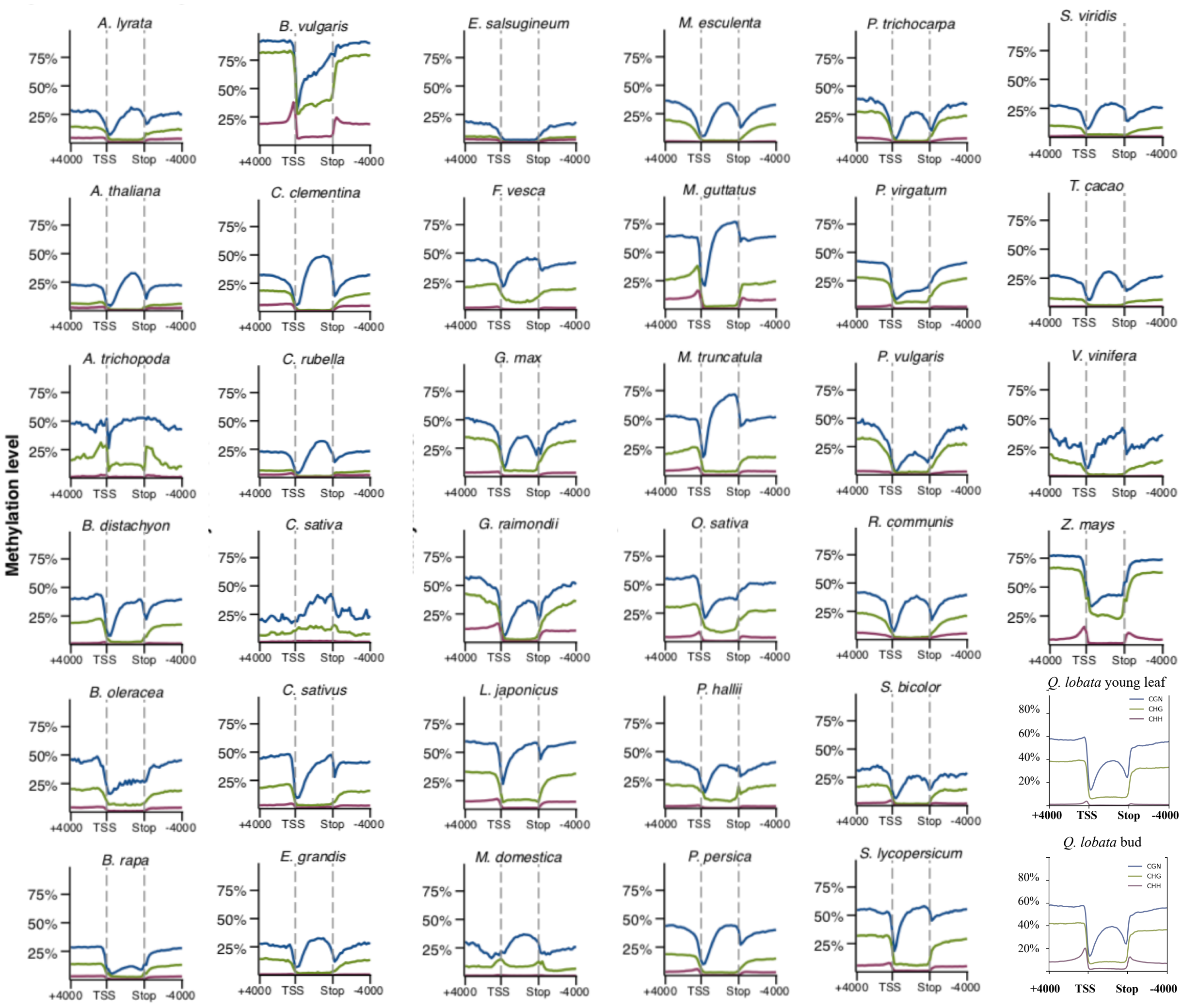

Supplementary Figure 22 (page 1 of 2)**. Chromosomal overviews of methylation and PCGs in oak compared with 24 angiosperms.** Plots are reprinted from Figure S10 in Niederhuth, Bewick [48], except limited to 24 taxa each having a chromosome-level assembly, and to which we add plots for *Q. lobata* with as similar methods as possible. **(A)** Subpanels are ordered column–to–column approximately by PCG density from heterogeneous to homogeneous. Subpanels show methylation levels and gene counts for100 kbp windows every 50 kbp across chr. 1 or the largest scaffold for each taxon. For *Q. lobata*, chr. 2 was used since is unusual (the lone acrocentric chromosome). mCG is shown in blue, mCHG in green, mCHH in maroon, and gene counts in red. Despite having a relatively high total PCG count (39,373), oaks are among the lowest for chromosome arm gene density. Methylation levels also usually correlate with prevalence of repeats, and show very distinct patterns in the initial columns vs. much more homogenous levels toward the later columns. Gene count *y*-axis upper limit is variable (determined by peak). (**B**) *Z. mays* and *Q. lobata* are placed side by side to show similarity, with *Q. lobata* gene count *y*-axis plotted matching that of *Z. mays*. (**C**) Methylation and PCG counts for all twelve *Q. lobata* chromosomes.

A.

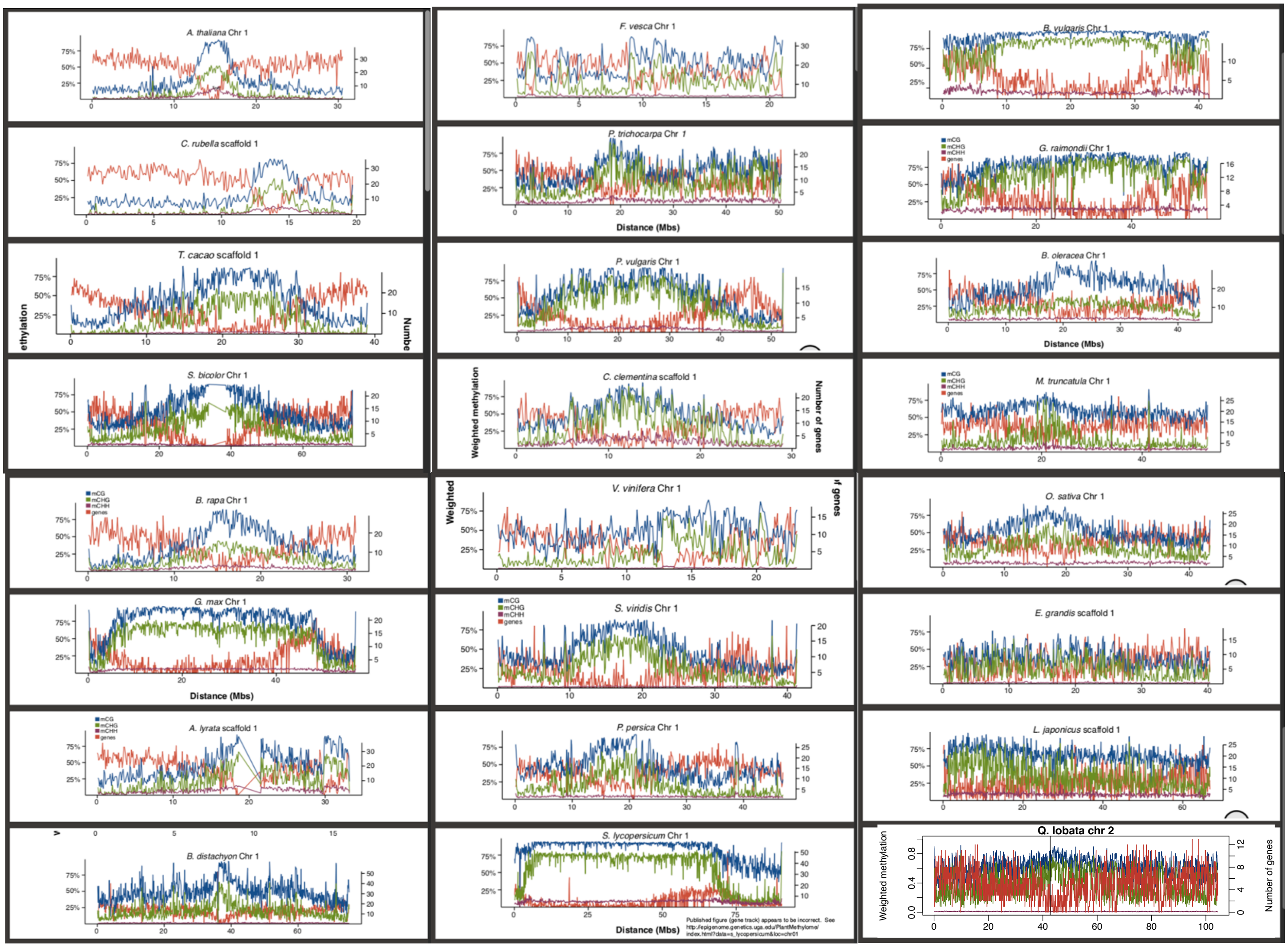

(continued Supplementary Figure 22, page 2 of 2**)**

B.

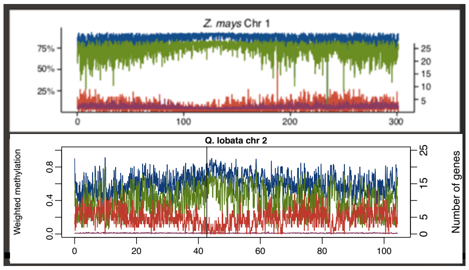

C.

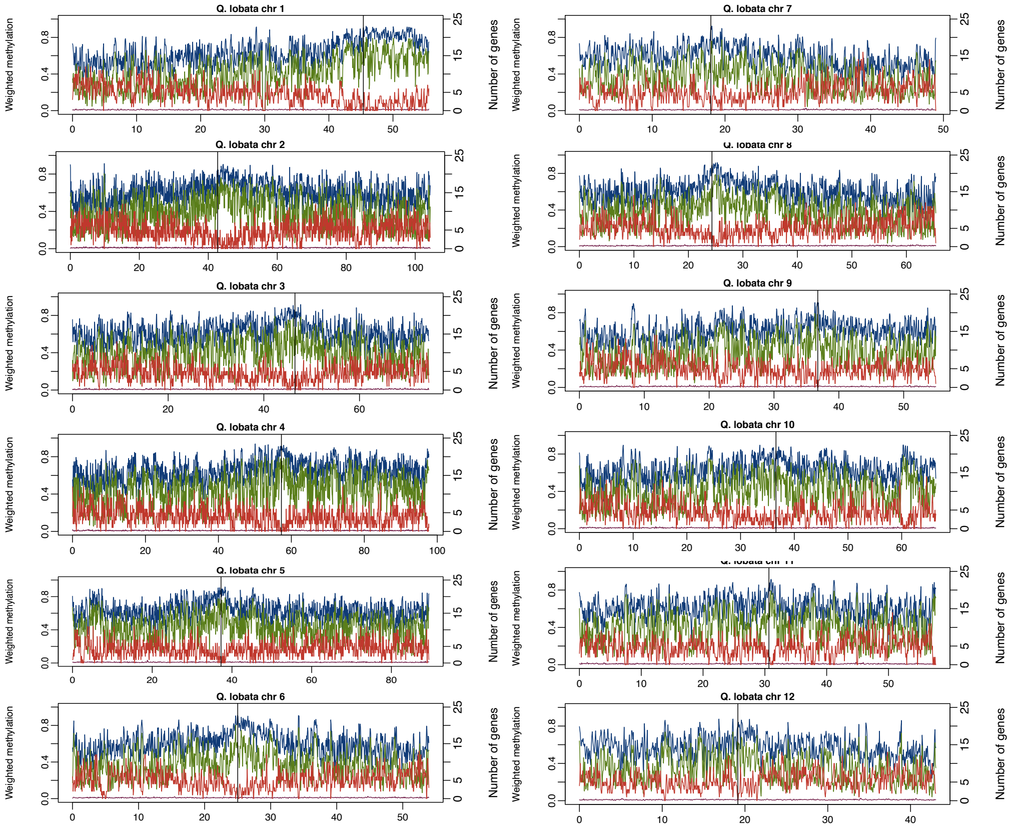

Supplementary Figure 23. Subcontext methylation for *Populus trichocarpa* chromosomes in 1 Mbp windows. Plots show mean mCHH by 3 nt subcontext in 1 Mbp windows every 1 Mbp. Methylation data is from tree 13.1 of Hofmeister *et al.* [49].

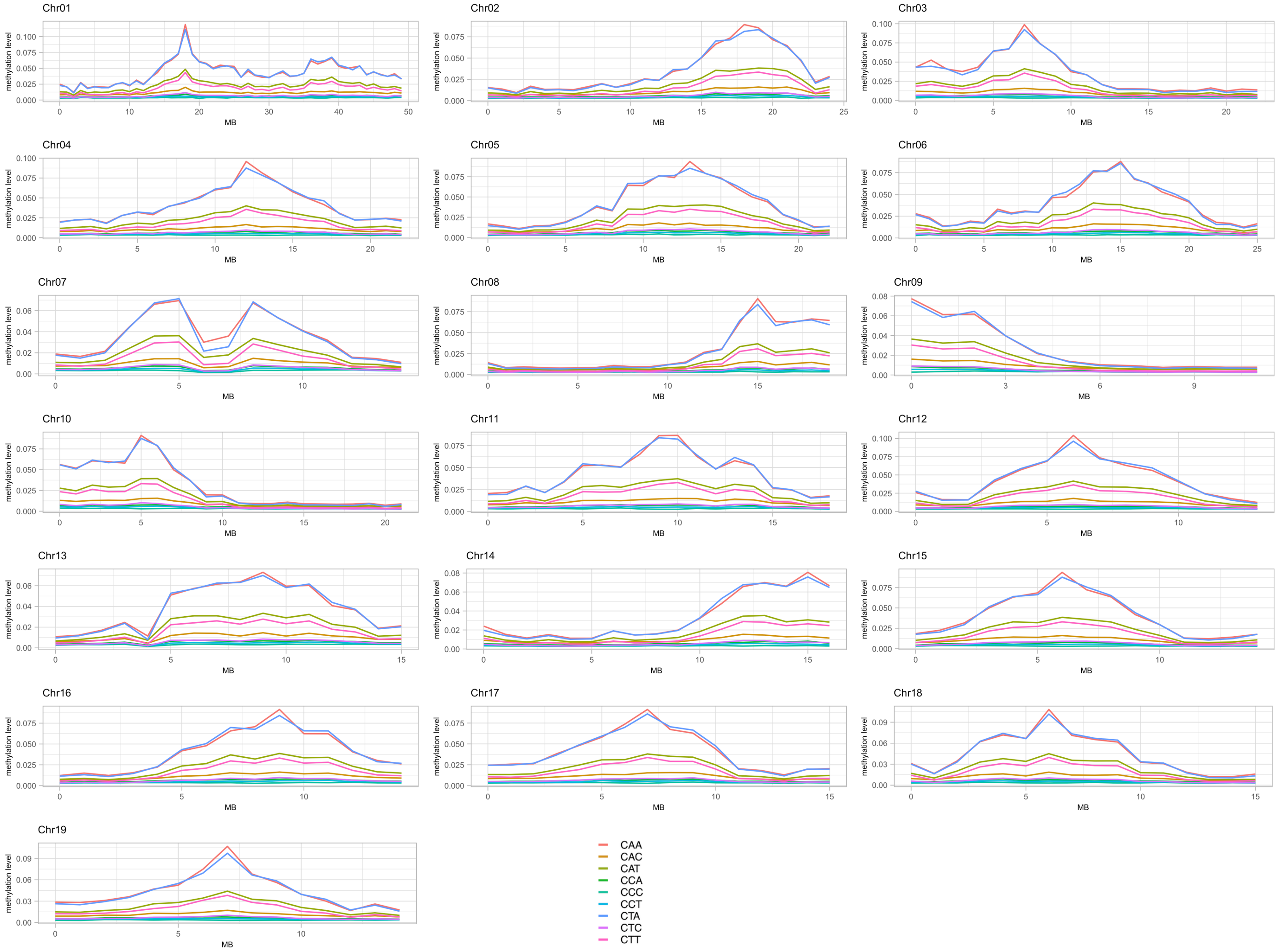

Supplementary Note 9. Additional Tables

Supplementary Table 5. **Top Pfam accessions enriched in the most heavily tandemly duplicated PCG families.** (Subsetted from **Supplementary Data 2**.) The 414 PCGs in the enrichment set are those participating in at least one tandem block of size 30 PCGs. Each list shows enrichment for Pfam domains with Benjamini-Hochberg FDR-adjusted *q*-value < 0.1, or (one-sided) hypergeometric *p*-value < 0.0002. Tandemness is defined via global amino acid identity ≥ 30%.

Pfam short name	*Hgeo. p-value*	*BH FDR q-value*	*Obs./ expect*	*Subset has…*	*in:*	*Bkgnd. has…*	*in:*	*Pfam accn.*	*Pfam type*	*Pfam long name*
**DUF247**	6.69E-87	2.78E-83	23.06	80	1,451	185	77,362	PF03140	Family	Plant protein of unknown function
**Stress-anti-fung**	5.41E-67	1.12E-63	13.54	82	1,451	323	77,362	PF01657	Family	Salt stress response/antifungal
**FBA_3**	1.14E-54	1.58E-51	14.15	65	1,451	245	77,362	PF08268	Domain	F-box associated domain
**NB-ARC**	8.58E-48	8.91E-45	5.39	113	1,451	1,118	77,362	PF00931	Domain	NB-ARC domain
**FBA_1**	1.05E-33	8.69E-31	11.79	44	1,451	199	77,362	PF07734	Family	F-box associated
**ADH_N_2**	5.01E-29	3.47E-26	34.99	21	1,451	32	77,362	PF16884	Family	N-terminal domain of oxidoreductase
**F-box**	1.29E-28	7.66E-26	5.64	64	1,451	605	77,362	PF00646	Domain	F-box domain
**Pkinase**	6.49E-28	3.37E-25	3.09	123	1,451	2,125	77,362	PF00069	Domain	Protein kinase domain
**Pkinase_Tyr**	1.19E-26	5.50E-24	2.99	123	1,451	2,196	77,362	PF07714	Domain	Protein tyrosine kinase
**ADH_zinc_N**	1.16E-21	4.84E-19	12.20	27	1,451	118	77,362	PF00107	Family	Zinc-binding dehydrogenase
**ADH_zinc_N_2**	1.74E-18	6.56E-16	18.46	18	1,451	52	77,362	PF13602	Domain	Zinc-binding dehydrogenase
**PPR_1**	4.05E-15	1.40E-12	2.48	92	1,451	1,980	77,362	PF12854	Repeat	PPR repeat
**S_locus_ glycop**	4.78E-15	1.53E-12	6.08	30	1,451	263	77,362	PF00954	Domain	S-locus glycoprotein domain
**PAN_2**	1.08E-14	3.20E-12	6.14	29	1,451	252	77,362	PF08276	Domain	PAN-like domain
**DUF3403**	2.72E-14	7.52E-12	9.61	20	1,451	111	77,362	PF11883	Family	Domain of unknown function (DUF3403)
**LRRNT_2**	4.71E-11	1.22E-08	3.25	42	1,451	689	77,362	PF08263	Family	Leucine rich repeat N-terminal domain
**LRR_1**	1.26E-10	3.09E-08	2.44	64	1,451	1,400	77,362	PF00560	Repeat	Leucine Rich Repeat
**B_lectin**	2.13E-10	4.92E-08	4.12	29	1,451	375	77,362	PF01453	Domain	D-mannose binding lectin
**F-box-like**	2.97E-10	6.50E-08	4.85	24	1,451	264	77,362	PF12937	Domain	F-box-like
**PPR_2**	1.28E-04	2.65E-02	1.51	85	1,451	2,994	77,362	PF13041	Repeat	PPR repeat family
**LRR_8**	2.79E-04	5.52E-02	1.68	51	1,451	1,616	77,362	PF13855	Repeat	Leucine rich repeat

Supplementary Table 6. Within 23,174 non-tandemly duplicated genes, top hypergeometrically-enriched accessions for those genes participating in at least two SSB-supporting gene pairs. (Subsetted from **Supplementary Data 2**.) The enrichment set contains 955 PCGs. Listed domains have Benjamini-Hochberg FDR-adjusted *q*-value < 0.05, or (one-sided) hypergeometric *p*-value < 0.0005. Tandemness is defined via global amino acid identity ≥ 30%.

Pfam short name	*Hgeo. p-value*	*BH FDR q-value*	*Obs./ expect*	*Subset has…*	*in:*	*Bkgnd. has…*	*in:*	*Pfam accn.*	*Pfam type*	*Pfam long name*	*Note*
**AP2**	6.78E-13	2.82E-09	5.52	26	1,920	103	42,020	PF00847	Domain	AP2 domain	Transcription factor
**WRKY**	1.90E-08	3.95E-05	5.17	17	1,920	72	42,020	PF03106	Domain	WRKY DNA-binding domain	Transcription factor
**ATP-synt_C**	2.34E-07	2.27E-04	16.41	6	1,920	8	42,020	PF00137	Family	ATP synthase subunit C	Enzyme
**Roc**	2.73E-07	2.27E-04	4.90	15	1,920	67	42,020	PF08477	Domain	Ras of Complex, Roc, domain of DAPkinase	Signal transduction
**DUF4050**	2.34E-07	2.27E-04	16.41	6	1,920	8	42,020	PF13259	Family	Protein of unknown function (DUF4050)	Unknown
**Ras**	7.37E-07	5.10E-04	4.56	15	1,920	72	42,020	PF00071	Domain	Ras family	Signal transduction
**Myb_DNA-binding**	2.14E-06	1.16E-03	2.46	33	1,920	294	42,020	PF00249	Domain	Myb-like DNA-binding domain	Transcription factor
**zf-Dof**	2.23E-06	1.16E-03	8.34	8	1,920	21	42,020	PF02701	Family	Dof domain, zinc finger	Transcription factor
**zf-C3HC4_2**	2.94E-06	1.35E-03	3.17	21	1,920	145	42,020	PF13923	Domain	Zinc finger, C3HC4 type (RING finger)	Transcription factor
**Hpt**	4.35E-06	1.80E-03	21.89	4	1,920	4	42,020	PF01627	Family	Hpt domain	Signal transduction
**RRM_5**	5.09E-06	1.92E-03	6.57	9	1,920	30	42,020	PF13893	Domain	RNA recognition motif (a.k.a. RRM/RBD/RNP domain)	RNA binding
**zf-C3HC4**	6.90E-06	2.00E-03	2.76	24	1,920	190	42,020	PF00097	Domain	Zinc finger, C3HC4 type (RING finger)	Transcription factor
**zf-RanBP**	7.14E-06	2.00E-03	7.30	8	1,920	24	42,020	PF00641	Domain	Zn-finger in Ran binding protein and others	Transcription factor
**Myb_DNA-bind_6**	7.69E-06	2.00E-03	2.68	25	1,920	204	42,020	PF13921	Domain	Myb-like DNA-binding domain	Transcription factor
**zf-C3HC4_3**	7.70E-06	2.00E-03	4.31	13	1,920	66	42,020	PF13920	Domain	Zinc finger, C3HC4 type (RING finger)	Transcription factor
**HCO3_cotransp**	6.58E-06	2.00E-03	10.94	6	1,920	12	42,020	PF00955	Family	HCO3– transporter family	Transporter
**Abhydrolase_2**	2.09E-05	5.11E-03	17.51	4	1,920	5	42,020	PF02230	Domain	Phospholipase/Carboxylesterase	Enzyme
**EamA**	2.31E-05	5.33E-03	4.97	10	1,920	44	42,020	PF00892	Family	EamA-like transporter family	Transporter
**Pkinase_Tyr**	5.11E-05	1.12E-02	1.68	63	1,920	822	42,020	PF07714	Domain	Protein tyrosine kinase	Signal transduction
**Pkinase**	5.41E-05	1.12E-02	1.69	61	1,920	790	42,020	PF00069	Domain	Protein kinase domain	Signal transduction
**DUF1218**	7.26E-05	1.31E-02	9.95	5	1,920	11	42,020	PF06749	Family	Protein of unknown function (DUF1218)	Cell wall
**SBP**	7.23E-05	1.31E-02	7.72	6	1,920	17	42,020	PF03110	Domain	SBP domain	Transcription factor
**Na_Ca_ex**	7.23E-05	1.31E-02	7.72	6	1,920	17	42,020	PF01699	Family	Sodium/calcium exchanger protein	Transporter
**Pec_lyase_N**	9.53E-05	1.52E-02	21.89	3	1,920	3	42,020	PF04431	Family	Pectate lyase, N-terminus	Cell wall
**GSDH**	9.53E-05	1.52E-02	21.89	3	1,920	3	42,020	PF07995	Domain	Glucose/Sorbosone dehydrogenase	Enzyme
**V-SNARE**	9.53E-05	1.52E-02	21.89	3	1,920	3	42,020	PF05008	Family	Vesicle transport v-SNARE protein N-terminus	Transporter
**Pec_lyase_C**	1.20E-04	1.79E-02	9.12	5	1,920	12	42,020	PF00544	Domain	Pectate lyase	Cell wall
**zf-C2H2_6**	1.21E-04	1.79E-02	3.82	11	1,920	63	42,020	PF13912	Domain	C2H2-type zinc finger	Transcription factor
**Gtr1_RagA**	1.63E-04	2.33E-02	5.67	7	1,920	27	42,020	PF04670	Domain	Gtr1/RagA G protein conserved region	Signal transduction
**DPBB_1**	2.01E-04	2.78E-02	6.57	6	1,920	20	42,020	PF03330	Domain	Lytic transglycolase	Enzyme
**Glyco_hydro_42**	2.62E-04	3.51E-02	10.94	4	1,920	8	42,020	PF02449	Domain	Beta-galactosidase	Enzyme
**Rer1**	3.68E-04	4.13E-02	16.41	3	1,920	4	42,020	PF03248	Family	Rer1 family	Membrane
**Remorin_N**	3.68E-04	4.13E-02	16.41	3	1,920	4	42,020	PF03766	Family	Remorin, N-terminal region	Membrane
**Bap31**	3.68E-04	4.13E-02	16.41	3	1,920	4	42,020	PF05529	Family	B-cell receptor-associated protein 31-like	Membrane
**Ribosom_S12_S23**	3.68E-04	4.13E-02	16.41	3	1,920	4	42,020	PF00164	Family	Ribosomal protein S12/S23	Ribosome
**PABP**	3.68E-04	4.13E-02	16.41	3	1,920	4	42,020	PF00658	Family	Poly-adenylate binding protein, unique domain	RNA binding
**Y_phosphatase2**	3.68E-04	4.13E-02	16.41	3	1,920	4	42,020	PF03162	Domain	Tyrosine phosphatase family	Signal transduction
**EF-hand_1**	4.24E-04	4.63E-02	2.61	16	1,920	134	42,020	PF00036	Domain	EF hand	Signal transduction
**PAE**	4.55E-04	4.85E-02	9.73	4	1,920	9	42,020	PF03283	Family	Pectinacetylesterase	Cell wall

Supplementary Table 7. Most abundant Pfam accessions in *Q. lobata*, and their frequency in selected other plant species. Counts are the number of protein sequences with one or more copy of the stated Pfam accession. **Red bold type** highlights largest value among the six tree species for each row. *Q. lobata* data are from our annotation via InterProScan 5.34-73.0 (https://www.ebi.ac.uk/interpro/about/interproscan/), and non-*Q. lobata* data is from https://pfam.xfam.org/ .

	Comparison tree group						Other species for comparison					
Species:	*Quercus lobata* (valley oak)	*Eucalyptus grandis* (Flooded gum)	*Juglans regia* (English walnut)	*Populus trichocarpa* (Western balsam poplar)	*Prunus persica* (Peach)	*Theobroma cacao* (Cacao)	*Amborella trichopoda*	*Arabidopsis thaliana* (Mouse-ear cress)	*Solanum lycopersicum* (Tomato)	*Oryza sativa* subsp. *indica* (Rice)	*Vitis vinifera* (Grape)	*Zea mays* (Maize)
Number of protein sequences:	39,373	44,149	45,533	53,333	38,726	40,614	27,369	39,359	34,634	37,383	29,903	99,234
Pfam accession:												
**PF00069** Protein kinase domain	1,287	**1,743**	1,396	1,501	1,043	964	446	1,001	717	953	824	2,813
**PF00931** NB-ARC domain	**1,031**	795	421	681	472	294	119	318	238	481	347	257
**PF13855** Leucine rich repeat	851	**1,003**	654	903	534	530	223	358	311	405	438	568
**PF07714** Protein tyrosine kinase	790	1,001	815	**1,073**	621	596	215	630	363	460	487	1,140
**PF08263** Leucine rich repeat N-terminal domain	**679**	614	473	535	362	379	136	282	266	344	233	466
**PF13041** PPR repeat family	**674**	534	562	632	561	538	549	449	423	405	505	607
**PF01535** PPR repeat	**669**	499	512	546	517	493	493	450	391	408	476	600
**PF00646** F-box domain	**541**	210	171	221	278	213	103	654	209	375	99	187
**PF00067** Cytochrome P450	507	**614**	408	447	328	345	234	326	309	383	385	413
**PF18052** Rx N-terminal domain	**489**	144	125	197	183	156	33	24	63	388	152	190
**PF00560** Leucine Rich Repeat	**448**	414	262	316	149	199	69	149	123	155	154	118
**PF01582** TIR domain	415	**426**	246	264	183	25	25	250	39	0	77	3
**PF13966** zinc-binding in reverse transcriptase	**410**	2	187	2	29	96	1	25	21	70	12	41
**PF14111** DUF4283	**408**	45	187	96	30	98	18	23	39	33	8	9
**PF01453** D-mannose binding lectin	**355**	321	191	276	144	130	39	98	79	128	102	96
**PF13456** Reverse transcriptase-like	323	21	**446**	21	92	299	10	67	43	85	7	12
**PF00201** UDP-glucoronosyl and UDP-glucosyl transferase	300	**376**	190	241	198	170	124	131	153	186	224	182
**PF00249** Myb-like DNA-binding domain	275	291	**454**	445	289	277	123	324	246	230	230	512
**PF00076** RNA recog. motif (a.k.a. RRM, RBD, or RNP domain)	260	315	**512**	478	405	415	179	382	250	254	212	1,166
**PF00954** S-locus glycoprotein domain	251	**287**	148	235	104	109	26	86	53	108	93	84

Supplementary References

1. Delfino Mix A, Wright JW, Gugger PF, Liang C, Sork VL. Establishing a range-wide provenance test in valley oak (*Quercus lobata* Née) at two California sites. In: *Proceedings of the seventh California oak symposium: managing oak woodlands in a dynamic world.* (eds Standiford RB, Purcell KL). U.S. Department of Agriculture, Forest Service, Pacific Southwest Research Station (2015).
2. Browne L, Wright JW, Fitz-Gibbon S, Gugger PF, Sork VL. Adaptational lag to temperature in valley oak (*Quercus lobata*) can be mitigated by genome-informed assisted gene flow. *Proceedings of the National Academy of Sciences* **50**, 25179-25185 (2019).
3. Gugger PF, Cokus SJ, Pellegrini M, Sork VL. Association of transcriptome-wide sequence variation with climate gradients in valley oak (*Quercus lobata*). *Tree Genetics & Genomes* **12**, 1-14 (2016).
4. Gugger PF, Fitz-Gibbon S, Pellegrini M, Sork VL. Species-wide patterns of DNA methylation variation in *Quercus lobata* and their association with climate gradients. *Molecular Ecology* **25**, 1665- 1680 (2016).
5. Kim BY*, et al.* RADseq data reveal ancient, but not pervasive, introgression between Californian tree and scrub oak species (*Quercus* sect. *Quercus*: Fagaceae). *Molecular ecology* **22**, 4556-4571 (2018).
6. Sork VL*, et al.* First draft assembly and annotation of the genome of a California endemic oak *Quercus lobata* Née (Fagaceae). *G3-Genes Genomes Genetics* **6**, 3485-3495 (2016).
7. Gordon SP*, et al.* Widespread polycistronic transcripts in fungi revealed by single-molecule mRNA sequencing. *PLOS ONE* **10**, e0132628 (2015).
8. Lieberman-Aiden E*, et al.* Comprehensive mapping of long-range interactions reveals folding principles of the human genome. *Science* **326**, 289-293 (2009).
9. Li JT, Yang J, Chen DC, Zhang XL, Tang ZS. An optimized mini-preparation method to obtain high-quality genomic DNA from mature leaves of sunflower. *Genetic and Molecular Research* **6**, 1064-1071 (2007).
10. Doyle JJ, Doyle JL. A rapid DNA isolation procedure for small quantities of fresh leaf tissue. *Phytochemical Bulletin* **19**, 11-15 (1987).
11. Martin M. Cutadapt removes adapter sequences from high-throughput sequencing reads (2011).
12. Li H, Durbin R. Fast and accurate short read alignment with Burrows-Wheeler transform. *Bioinformatics* **25**, 1754-1760 (2009).
13. Li H. Minimap2: pairwise alignment for nucleotide sequences. *Bioinformatics* **34**, 3094-3100 (2018).
14. Krueger F. Trim Galore v0.4.4. Preprint at http://www.bioinformatics.babraham.ac.uk/projects/trim_galore/. (2017).
15. Andrews S. FastQC v0.11.2.) (2014).
16. Schultz MD*, et al.* Human body epigenome maps reveal noncanonical DNA methylation variation. *Nature* **523**, 212-216 (2015).
17. Langmead B, Salzberg SL. Fast gapped-read alignment with Bowtie 2. *Nature Methods* **9**, 357-359 (2012).
18. Ryan DP. MethylDackel: A (mostly) universal methylation extractor for BS-Seq Experiments,. (2019).
19. Ramírez F*, et al.* deepTools2: a next generation web server for deep-sequencing data analysis. *Nucleic Acids Research* **44**, W160-W165 (2016).
20. Bodénès C, Chancerel E, Ehrenmann F, Kremer A, Plomion C. High-density linkage mapping and distribution of segregation distortion regions in the oak genome. *DNA Research* **23**, 115-124 (2016).
21. Hipp AL*, et al.* Sympatric parallel diversification of major oak clades in the Americas and the origins of Mexican species diversity. *New Phytologist* **217**, 439-452 (2018).
22. Lepoittevin C*, et al.* Single-nucleotide polymorphism discovery and validation in high-density SNP array for genetic analysis in European white oaks. *Molecular Ecology Resources* **15**, 1446-1459 (2015).
23. Plomion C*, et al.* Oak genome reveals facets of long lifespan. *Nature Plants* **4**, 440-452 (2018).
24. Tajima F. Evolutionary relationship of DNA sequences in finite populations. *Genetics* **105**, 437-460 (1983).
25. Miles A, Ralph P, Rae S, Pisupati R. cggh/scikit-allel: v1.2.1. In: *Zenodo*) (2019, June 4).
26. Schiffels S, Durbin R. Inferring human population size and separation history from multiple genome sequences. *Nature Genetics* **46**, 919-925 (2014).
27. Bosse M*, et al.* Untangling the hybrid nature of modern pig genomes: a mosaic derived from biogeographically distinct and highly divergent *Sus scrofa* populations. *Molecular Ecology* **23**, 4089-4102 (2014).
28. Fitak RR, Mohandesan E, Corander J, Burger PA. The *de novo* genome assembly and annotation of a female domestic dromedary of North African origin. *Molecular Ecology Resources* **16**, 314–324 (2016).

29. Wallberg A, Fan Han, Gustaf Wellhagen, Bjørn Dahle, Masakado Kawata, Nizar Haddad, Zilá Luz Paulino Simões, et al. A worldwide survey of genome sequence variation provides insight into the evolutionary history of the honeybee *Apis mellifera*. *Nature Genetic* **46**, 1081-1088 (2014).
30. Amborella Genome Project. The Amborella genome and the evolution of flowering plants. *Science* **342**, 1241089–1241089 (2013).
31. Holliday JA, Zhou L, Bawa R, Zhang M, Oubida RW. Evidence for extensive parallelism but divergent genomic architecture of adaptation along altitudinal and latitudinal gradients in *Populus trichocarpa*. *New Phytologist* **209**, 1240–1251 (2016).
32. Ibarra-Laclette E*, et al.* Architecture and evolution of a minute plant genome. *Nature* **498**, 94-98 (2013).
33. Chaw S-M*, et al.* Stout camphor tree genome fills gaps in understanding of flowering plant genome evolution. *Nature Plants* **5**, 63-73 (2019).
34. Bai W-N, Yan P-C, Zhang B-W, E.Woeste K, Lin K, Zhang D-Y. Demographically idiosyncratic responses to climate change and rapid Pleistocene iversification of the walnut genus *Juglans* (Juglandaceae) revealed by whole-genome sequences. *New Phytologist* **217**, 1726–1736 (2018).
35. Nadachowska-Brzyska K, Burri R, Olason PI, Kawakami T, Smeds La, Ellegren H. Demographic divergence history of pied flycatcher and collared flycatcher inferred from whole-genome re-sequencing data (2013).
36. Brown RW, Davis FW. Historical mortality of valley oak (*Quercus lobata*, Nee) in the Santa Ynez Valley, Santa Barbara County, 1938-1989. In: *Proceedings of the Symposium on Oak Woodlands and Hardwood Rangeland Management, October 31-November 2, 1990* (ed Standiford RB). U.S. Dept. of Agriculture, Pacific Southwest Research Station (1991).
37. Gillespie JH, Langley CH. Are evolutionary rates really variable? *Journal of Molecular Evolution* **13**, 27-34 (1979).
38. Marçais G, Delcher AL, Phillippy AM, Coston R, Salzberg SL, Zimin A. MUMmer4: A fast and versatile genome alignment system. *PLOS Computational Biology* **14**, e1005944 (2018).
39. Bobiwash K, Schultz ST, Schoen DJ. Somatic deleterious mutation rate in a woody plant: estimation from phenotypic data. *Heredity* **111**, 338-344 (2013).
40. Bromham L, Penny D. The modern molecular clock. *Nature Reviews Genetics* **4**, 216-224 (2003).
41. Garcia J, Zhen Y, Lohmueller K. Demographic history analysis scripts for *Quercus lobata* reference genome (v1.0.2). *Zenodo*, (2022).
42. Beichman AC, Phung TN, Lohmueller KE. Comparison of single genome and allele frequency data reveals discordant demographic histories. *G3: Genes|Genomes|Genetics* **7**, 3605-3620 (2017).
43. Larkin MA*, et al.* Clustal W and Clustal X version 2.0. *Bioinformatics* **23**, 2947-2948 (2007).
44. Kearse M*, et al.* Geneious Basic: an integrated and extendable desktop software platform for the organization and analysis of sequence data. *Bioinformatics* **28**, 1647-1649 (2012).
45. Round EK, Flowers SK, Richards EJ. *Arabidopsis thaliana* centromere regions: genetic map positions and repetitive DNA structure. *Genome Research* **7**, 1045-1053 (1997).
46. Roth MS*, et al.* Chromosome-level genome assembly and transcriptome of the green alga *Chromochloris zofingiensis* illuminates astaxanthin production. *Proceedings of the National Academy of Sciences* **114**, E4296-E4305 (2017).
47. Gururani MA, Venkatesh J, Upadhyaya CP, Nookaraju A, Pandey SK, Park SW. Plant disease resistance genes: current status and future directions. *Physiological and molecular plant pathology* **78**, 51-65 (2012).
48. Niederhuth CE*, et al.* Widespread natural variation of DNA methylation within angiosperms. *Genome biology* **17**, 194 (2016).
49. Hofmeister BT*, et al.* A genome assembly and the somatic genetic and epigenetic mutation rate in a wild long-lived perennial *Populus trichocarpa*. *Genome Biology* **21**, 259 (2020).
