# Supplementary material for: High-quality genome and methylomes illustrate features underlying evolutionary success of oaks
Source: Nat Commun. 2022 Apr 19;13:2047. doi: 10.1038/s41467-022-29584-y (PMC9018854; doi:10.1038/s41467-022-29584-y)
Supplement: Supplementary file 2 — Description of Additional Supplementary Files [file 41467_2022_29584_MOESM2_ESM.pdf]

Description of Additional Supplementary Files

File name**: Supplementary Data 1**

Description: Excel spread sheet showing methylation trends. Adapted from the paper *Widespread natural variation of DNA methylation within angiosperms* (Niederhuth, C. E. et al. 2016. Genome biology **17**, 194. (https://genomebiology.biomedcentral.com/articles/10.1186/s13059-016-1059-0). This file provides data used in Supplementary Table 2, giving basic methylation statistics for 34 angiosperms, to which we have added data for oak, plus additional columns derived from the original data: (1) rank of correlation of mCHH vs. gene content; (2) estimate of chromosome arm gene density; and (3) strength of mCHH islands with and without respect to background mCHH level.

File name**: Supplementary Data 2**

Description: Excel spreadsheet showing hypergeometric enrichment of Pfam domains and (separately) words found in gene names, for subsets of protein coding genes (PCGs) defined variously by degree of tandem duplication, membership in self-syntenic blocks (SSBs), intron size, expression level, protein length, and local repeat density.
