# Supplementary material for: High-quality genome and methylomes illustrate features underlying evolutionary success of oaks
Source: Nat Commun. 2022 Apr 19;13:2047. doi: 10.1038/s41467-022-29584-y (PMC9018854; doi:10.1038/s41467-022-29584-y)
Supplement: Supplementary file 5 — Reporting Summary [file 41467_2022_29584_MOESM5_ESM.pdf]

nature research

Corresponding author(s): Victoria L. Sork

Last updated by author(s): Mar 17, 2021

Reporting Summary

Nature Research wishes to improve the reproducibility of the work that we publish. This form provides structure for consistency and transparency in reporting. For further information on Nature Research policies, see our Editorial Policies and the Editorial Policy Checklist.

Statistics

For all statistical analyses, confirm that the following items are present in the figure legend, table legend, main text, or Methods section.

n/a	Confirmed	
☐	☒	The exact sample size (n) for each experimental group/condition, given as a discrete number and unit of measurement
☒	☐	A statement on whether measurements were taken from distinct samples or whether the same sample was measured repeatedly
☒	☐	The statistical test(s) used AND whether they are one- or two-sided<br>*Only common tests should be described solely by name; describe more complex techniques in the Methods section.*
☒	☐	A description of all covariates tested
☒	☐	A description of any assumptions or corrections, such as tests of normality and adjustment for multiple comparisons
☒	☐	A full description of the statistical parameters including central tendency (e.g. means) or other basic estimates (e.g. regression coefficient) AND variation (e.g. standard deviation) or associated estimates of uncertainty (e.g. confidence intervals)
☒	☐	For null hypothesis testing, the test statistic (e.g. F, t, r) with confidence intervals, effect sizes, degrees of freedom and P value noted<br>*Give P values as exact values whenever suitable.*
☒	☐	For Bayesian analysis, information on the choice of priors and Markov chain Monte Carlo settings
☒	☐	For hierarchical and complex designs, identification of the appropriate level for tests and full reporting of outcomes
☒	☐	Estimates of effect sizes (e.g. Cohen's d, Pearson's r), indicating how they were calculated

Our web collection on statistics for biologists contains articles on many of the points above.

Software and code

Policy information about availability of computer code

Data collection

Provide a description of all commercial, open source and custom code used to collect the data in this study, specifying the version used OR state that no software was used.

Data analysis

For demographic inference, we used MSMC 1.1.0 (https://github.com/stschiff/msmc). Coalescent simulations were run using msprime 0.7.4 (https://github.com/tskit-dev/msprime). The code for PSMC files and masks is available at https://doi.org/10.5281/zenodo.5899535. For computation of mutation rates, we used MUMMER 4.4.0 for genome alignments. For genome assembly, comparisons and annotation we used the following software as described in the methods: MaSuRCA 3.2.1, Dovetail HiRise re-scaffolder, Modified SNAP, BLASTN+ 2.2.30, LASTZ, Diamond, and Parasail, C++, Mathematica, Perl, ImageMagick, Adobe Photoshop, InterProScan, HMMer, needle' from EMBOSS v6.6.0.0, pal2nal.pl v.14, codeml from PAML v.4.9i, RepeatModeler/Classifier open-1.0.8, PSI-CD-HIT 4.7, LTRharvest and LTRdigest from GenomeTools 1.5.9, Minimap2 v. 2.17-r941, Diamond v0.9.22.123, Exonerate v2.4.0, Augustus, STAR v2.5.3a, StringTie v1.3.4d, Trinity v2.6.6, GMAP v2017-11-15, Interproscan v5.34-73.0, OMA, BUSCO. For methylome and resequenced genomes analysis we used the following software as described in the methods: Methylpy v. 1.4.6, DeepTools v.3.1.2, R v3.6.0, R ggplot2 library v3.3.2, bed12toAnnotation.awk, MethylDackel v0.4.0, TrimGalore! v.0.4.4, bwa mem v.0.7.12-r1039, Picard tools v.2.13.2-SNAPSHOT, GATK v3.7-0-gcfedb67, BLASTN 2.2.30+

For manuscripts utilizing custom algorithms or software that are central to the research but not yet described in published literature, software must be made available to editors and reviewers. We strongly encourage code deposition in a community repository (e.g. GitHub). See the Nature Research guidelines for submitting code & software for further information.

Data

Policy information about availability of data

All manuscripts must include a data availability statement. This statement should provide the following information, where applicable:

- Accession codes, unique identifiers, or web links for publicly available datasets
- A list of figures that have associated raw data
- A description of any restrictions on data availability

All data other than variants are accessible under NCBI Bioproject ID: PRJNA781973, which includes Q.lobata v3.0 genome assembly (NCBI: PRJNA308314). DNA sequencing reads for this project are available from SRA under accession numbers SRX10889646 through SRX10889720 (75 files) and SRX10972769 throughSRX10972854 (86 files). Data can also be found through the project website (https://valleyoak.ucla.edu/), and the project UCSC genome browser (http://genomes.mcdb.ucla.edu/cgi-bin/hgTracks?db=queLob3). For the 19 resequenced trees, fastq files available at NCBI: PRJNA729978. Data are also accessible through the Valley Oak website (valleyoak.ucla.edu) under Genomic Resources.

Field-specific reporting

Please select the one below that is the best fit for your research. If you are not sure, read the appropriate sections before making your selection.

☒ Life sciences ☐ Behavioural & social sciences ☐ Ecological, evolutionary & environmental sciences

For a reference copy of the document with all sections, see nature.com/documents/nr-reporting-summary-flat.pdf

Life sciences study design

All studies must disclose on these points even when the disclosure is negative.

Sample size	No power calculations were performed. For demographic inference, studies have shown that PSMC' performs better when analyzing a single genome at a time, rather than multiple genomes (Beichman et al. 2017; Adrion et al. 2020). Further, we validated our demographic inference approach using simulations, as described in the manuscript.
Data exclusions	One resequenced genome was not included in the set of 19 genomes analyzed for demography because it had exceptionally high heterozygosity, suggesting it was a recent hybrid and/or contaminated.
Replication	We replicated the PSMC' demographic inference from our Q. lobata reference genome using 19 additional resequenced genomes.
Randomization	No randomization was necessary as samples were not assigned to experimental groups.
Blinding	No blinding was necessary as samples were not assigned to experimental groups.

Reporting for specific materials, systems and methods

We require information from authors about some types of materials, experimental systems and methods used in many studies. Here, indicate whether each material, system or method listed is relevant to your study. If you are not sure if a list item applies to your research, read the appropriate section before selecting a response.

Materials & experimental systems

n/a	Involved in the study	
☒	☐	Antibodies
☒	☐	Eukaryotic cell lines
☒	☐	Palaeontology and archaeology
☒	☐	Animals and other organisms
☒	☐	Human research participants
☒	☐	Clinical data
☒	☐	Dual use research of concern

Methods

n/a	Involved in the study	
☒	☐	ChIP-seq
☒	☐	Flow cytometry
☒	☐	MRI-based neuroimaging
